# The Genetic Diversity of Influenza A Viruses in Wild Birds in Peru

**DOI:** 10.1371/journal.pone.0146059

**Published:** 2016-01-19

**Authors:** Martha I. Nelson, Simon Pollett, Bruno Ghersi, Maria Silva, Mark P. Simons, Eliana Icochea, Armando E. Gonzalez, Karen Segovia, Matthew R. Kasper, Joel M. Montgomery, Daniel G. Bausch

**Affiliations:** 1 Fogarty International Center, National Institutes of Health, Bethesda, Maryland, United States of America; 2 Virology and Emerging Infections Department, United States Naval Medical Research Unit No. 6, Callao, Peru; 3 Marie Bashir Institute for Infectious Diseases and Biosecurity, University of Sydney, New South Wales, Australia; 4 Department of Epidemiology and Biostatistics, University of California San Francisco, San Francisco, California, United States of America; 5 Universidad Nacional Mayor de San Marcos, School of Veterinary Medicine, San Borja, Lima, Peru; 6 Tulane School of Public Health and Tropical Medicine, New Orleans, Louisiana, United States of America; Linneaus University, SWEDEN

## Abstract

Our understanding of the global ecology of avian influenza A viruses (AIVs) is impeded by historically low levels of viral surveillance in Latin America. Through sampling and whole-genome sequencing of 31 AIVs from wild birds in Peru, we identified 10 HA subtypes (H1-H4, H6-H7, H10-H13) and 8 NA subtypes (N1-N3, N5-N9). The majority of Peruvian AIVs were closely related to AIVs found in North America. However, unusual reassortants, including a H13 virus containing a PA segment related to extremely divergent Argentinian viruses, suggest that substantial AIV diversity circulates undetected throughout South America.

## Introduction

Wild aquatic birds are natural reservoirs for genetically diverse avian influenza A viruses (AIVs), which occasionally transmit to domestic poultry, horses, swine, canines, seals, and humans, sometimes causing major outbreaks and/or severe disease. There have been extensive efforts to understand the ecology and evolution of AIVs in wild birds, including how flyways spread viral diversity geographically, in order to understand the evolution of highly pathogenic viruses (HPAI) that threaten humans and poultry [1–4]. In general, AIVs are spatially separated into North American and Eurasian lineages, although sporadic intercontinental incursions occur, including the most notable spread of H5 viruses from Asia into North America [5]. However, surveillance of AIV is currently focused on North America, Europe, and Asia, and the recent identification of an AIV lineage in Argentinian wild birds that is highly divergent from the North American and Eurasian lineages [6] highlighted the great imbalance in our knowledge of AIV evolution in the Southern hemisphere [7].

To gain a greater understanding of AIV diversity in South America, we collected AIV positive specimens from wild birds in Peru during 2006–2011 and sequenced the viral genomes. Through phylogenetic analysis, we determined that the majority of viruses were closely related to AIVs found in North American birds, reflecting the fact that migration flyways often span the two continents, facilitating gene flow. However, we also identified a number of reassortant Peruvian viruses with segments positioned within many diverse phylogenetic lineages, including the divergent Argentinian lineage. These findings suggest that viruses not related to those found in North America and Eurasia may circulate extensively throughout South America, co-circulating and reassorting with North American and Eurasian viruses to generate unique viruses

## Materials and Methods

### AIV collection and sequencing

Environmental fecal samples were collected from 34 wild water bird species (13 families) from both the *Anseriformes* and *Charadriiformes* orders, including long and intermediate migratory waterfowl and local resident shorebirds (S1 Table) at 5 different wetland sites (Medio Mundo [Lat. -10.922 Long. -77.668], Puerto Viejo [Lat. -12.562 Long. -76.712], Paraiso [Lat. -11.212 Long -.77.595], Laguna de Chancay [Lat. -11.593 Long. -77.269], Pantanos de Villa [Lat.-12.212 Long. -76.987]) along the Peruvian coast (Lima province, S1 Fig). Details of collection are reported by Ghersi *et al* in full elsewhere [8,9]. Permission was obtained for environmental sampling at the Pantanos de Villa protected wetland site by the Instituto Nacional de Recursos Naturales, with no permission required at the remaining sites as the environmental sampling occurred on public lands which were not part of natural reserves. This study was determined as a no-animal handling study by IACUC protocol NMRCD-9-05. Fecal samples were processed and tested for the presence of influenza viruses as previously described [8,9]. Avian fecal samples were initially screened using isolation in embryonated chicken eggs and the allantoic fluid tested by direct hemagglutination assay. The influenza subtype of positive fluids was determined using HA and NA RT-PCR and universal HA and NA primers [10] and underwent whole genome sequencing using the using primers listed in S2 Table. Amplicons were sequenced by the Sanger-method using the Applied Biosystems 3730 system, and amplicons with poor sequence data were re-amplified and re-purified as necessary to get complete sequences.

### Phylogenetic analysis

Sequence alignments for each of the six internal gene segments, as well as for each of the HA and NA subtypes were generated using MUSCLE v3.8.31 [11]. These alignments included sequences from 31 AIVs collected in wild birds in Peru, as well as background AIV sequences available from the Influenza Virus Resource Database available at GenBank [12], including all AIV sequence data available from wild birds in other South American countries since 2000 as well as whole-genome sequences from 2,011 North American AIVs collected between 2006–2011, 155 Eurasian AIVs, and eight AIVs from Australian wild birds (accession numbers for these viruses are available in S3 Table). In order to include viruses for which only part of the segment’s coding region was successfully sequenced, alignments were trimmed to partial coding regions as needed for each segment: PB2 (1143 nt), PB1 (1941 nt), PA (1677 nt), NP (1494 nt), MP (981 nt), and NS (837 nt). Separate alignments of at least 800 nt were generated for each of the nine HA and eight NA subtypes, this length was restricted by the available study sequences. Phylogenetic relationships were inferred separately for each alignment using the maximum likelihood methods available in RAxML v7.2.6 [13], incorporating a general time-reversible (GTR) model of nucleotide substitution with a gamma-distributed (Γ) rate variation among sites. To assess the robustness of each node, a bootstrap resampling process was performed (500 replicates). To further understand the evolution of the H13 gull lineage, an additional phylogeny was inferred including all 85 H13 sequences available on GenBank (S4 Table). A neighbor-joining tree was inferred for the limited number of Peruvian N2 segments available (5 H13N2 viruses, 1 H3N2 virus, and 1 H10N2 virus), as well as for 221 N2 viruses collected from the Americas using the PAUP* software package [14].

## Results

**Fig 1:**
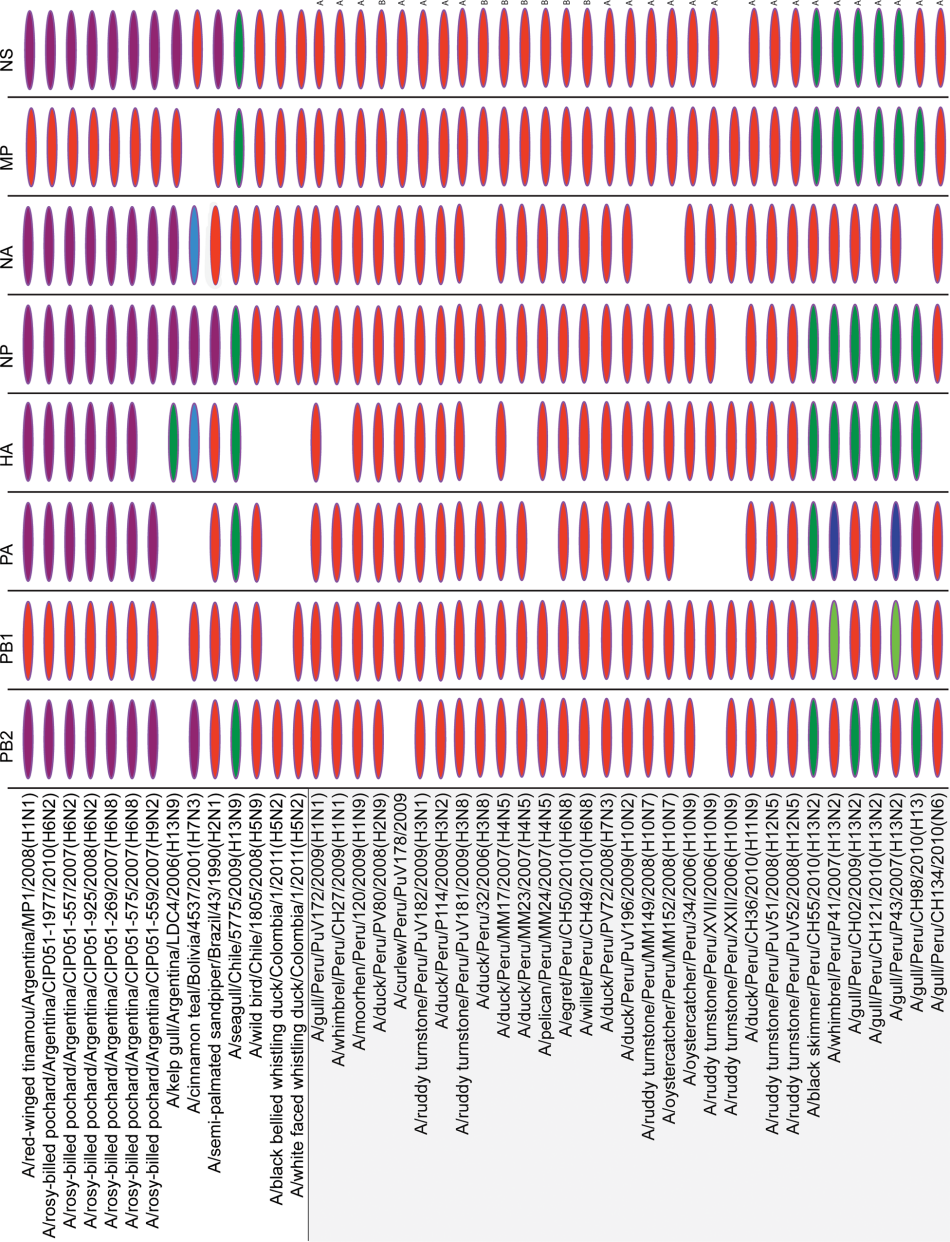
Genetic diversity of avian influenza viruses in South America. Phylogenetic position of 45 AIVs collected in wild birds in six countries in South America: Argentina, Bolivia, Brazil, Chile, Colombia, and Peru, for each of the eight genome segments: PB2, PB1, PA, HA, NP, NA, MP, and NS. Alleles for the Peruvian NS segments are indicated by ‘A’ or ‘B’. Shading corresponds to the phylogenetic position, as depicted in Fig 2 and S2–S14 Figs: Argentinian AIV lineage = purple; North American AIV lineage = red; global AIV lineage = green; Eurasian AIV lineage = blue; minor Peruvian AIV lineage (PB1 phylogeny, S3 Fig) = light green; and minor Bolivian lineage = light blue. Segments for which sequence data was not available are shaded white.

**Fig 2:**
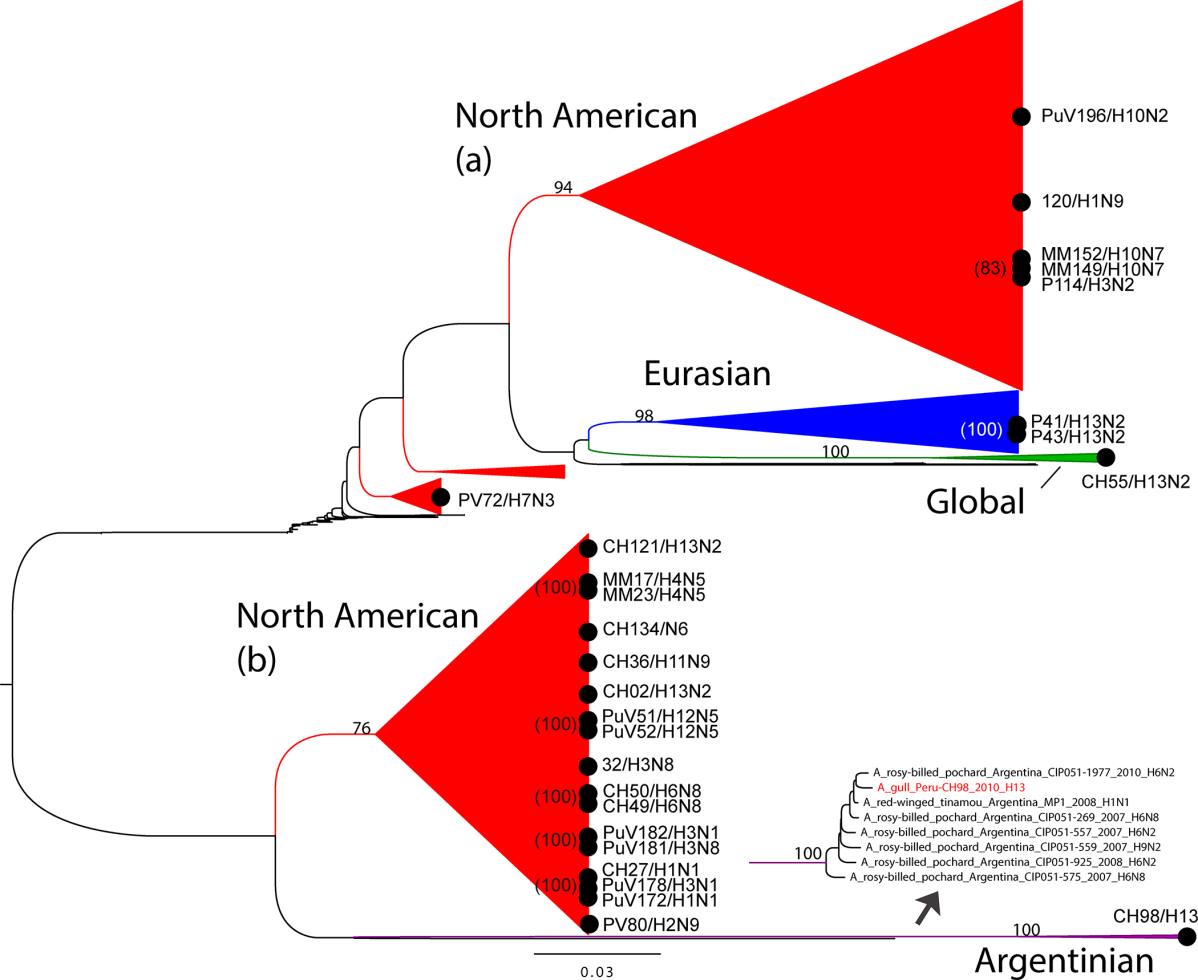
Phylogenetic relationships of the PA segment of AIVs in Peru. Maximum likelihood phylogeny inferred for 2,184 PA segments of AIVs collected globally, including 27 that were collected from wild birds in Peru. For clarity, major AIV lineages have been simplified as cartoon triangles, although the branches for the Argentinian lineage are depicted as an inset. Major AIV clades are shaded accordingly to lineage, corresponding to Fig 1: Argentinian AIV lineage = purple; North American AIV lineages = red; global AIV lineage = green; Eurasian AIV lineage = blue. Bootstrap values > 70 are provided for key nodes, the tree is midpoint rooted for clarity, and all branch lengths are drawn to scale. Scale bar indicates number of nucleotide substitutions per site. The phylogenetic position of the 27 viruses from Peru is indicated with a solid black circle, along with the identifier and subtype for each virus. Viruses that cluster together are indicated by solid black circles that overlap, with bootstrap values provided in parentheses. Each cluster of overlapping black circles or individual black circle thus represents a putative viral introduction into Peru.

The summary non-genomic results of surveillance have been presented in full detail elsewhere [8,9]. From 6887 collected fecal samples, 31 environmental specimens positive for AIV were successfully sequenced, although not all genome segments were able to be successfully amplified during the PCR process (see Fig 1). Fifteen distinct subtypes, all low pathogenic AIV, were detected from a wide range of hosts, including long-distance migratory shorebirds (e.g. Ruddy Turnstones (*Arenaria interpres*)) and more local residential species (e.g. Common Moorhens (*Gallinula chloropus*)) (Fig 1). Ten HA subtypes (H1, H2, H3, H4, H6, H7, H10, H11, H12, and H13) and eight NA subtypes (N1, N2, N3, N5, N6, N7, N8, and N9) were identified, representing substantial antigenic diversity. In general, most AIVs found in Peru (24/31) were closely related to North American AIVs in all gene segments (Figs 1 and 2, S2–S14 Figs). Peruvian AIVs of the same subtype often clustered together phylogenetically, consistent with local AIV transmission among sampled birds. Notably, Peruvian viruses spanned nearly the entire diversity of North American AIVs, indicating multiple putative introductions of genetically distinct North American AIVs into Peru (Fig 2, S2–S14 Figs). In fact, multiple putative viral introductions were observed during each year from 2007–2010 (S5 Table). In 2010, six independent viral introductions were identified on the PA phylogeny: four separate introductions of North American viruses, an introduction of a highly divergent Argentinian AIV lineage[6], and a Eurasian-like lineage that contains viruses from both the Western and Eastern hemispheres (labeled as ‘Global’, Fig 2). Considering evidence taken from all segments of the viral genome, there was no evidence for viral persistence across multiple years, indicating that new viral diversity is introduced into these Peruvian bird populations each year.

The introduction and co-circulation of multiple genetically distinct viral lineages each year affords the opportunity for reassortment and the generation of novel viruses. The most diverse and unusual reassortant viruses collected in our study were of the H13 subtype (Fig 1). In contrast, the H1 to H12 Peruvian segments studied all fell into a North American lineage, although reassortment events within this large North American lineage were also observed. Most notably, a number of Peruvian AIVs contained at least one segment that is positioned within phylogenetic clades not found in North America. The PA segment of a H13 virus (A/gull/Peru/CH98/2010(H13)) is phylogenetically positioned within a highly divergent clade of Argentinian AIVs of the H6 subtype (Fig 1, Fig 2 and S9 Fig). Viruses from wild birds in other South American countries also have segments that cluster within this divergent Argentinian lineage, including the NP and NS segments from the A/semi-palmated sandpiper/Brazil/43/1990(H2N1) strain (S10 and S12 Figs) and the PB2, NP, and NS segments of A/cinnamon teal/Bolivia/4537/2001(H7N3) (S7, S10, and S12 Figs). On the PB1 phylogeny (S3 and S8 Figs), two Peruvian H13N2 viruses (A/whimbrel/Peru/P41/2007(H13N2) and A/gull/Peru/P43/2007(H13N2)) are positioned basal to the main North American lineage, indicating possible independent evolution of an undersampled North American ‘sister’ lineage. These two viruses also contain segments that are more closely related to Eurasian AIVs (Fig 1). All six Peruvian H13 viruses also contained segments related to the spatially-mixed ‘global’ clade containing viruses from both Westerns and Eastern hemispheres (Fig 1).

AIV sequences from the Central American country of Guatemala also were included in the study. These Guatemala sequences were positioned within the North American lineages (e.g S12 Fig) and were not closely related to Peruvian strains.

## Discussion

Our surveillance efforts identified substantial genetic and antigenic diversity of AIVs circulating in Peruvian wild birds, including viral segments related to (a) North American viruses, (b) a clade of extremely divergent AIVs recently identified in Argentina [6], (c) a small number of viruses not closely related to any previously described AIV lineages and (d) a clade of viruses from North America and Eurasia that belong to a less spatially defined lineage found in gulls and shorebirds. These findings are consistent with regular migration of AIVs between North and South America, and annual viral re-introduction consistent with migration and the long-distance flyways of certain gulls and shorebirds that span North and South America, such as the Ruddy Turnstone.

Peru is one of the most climatically diverse regions of the world, with many different biomes in relative close proximity, and further understanding of the local dynamics of AIV among Peru’s migratory and resident bird populations is also needed. The high genetic diversity of AIV even in our relatively small sampling (31 sequenced viruses) suggests that Peru could be a mixing ground with many diverse viruses to co-circulate and reassort.

Most critically, our findings support a global ecological model of AIV in which gene flow occurs regularly between North America and South America, a finding which is supported by the recent detection of H13 AIV in Chilean wild birds that is closely related to North American AIV [15].There was no strong evidence of viral AIV traffic through Central American en route to South America. However, surveillance in Central America is limited to Guatemala at this time, and further sampling throughout Central and South America could perhaps clarify intercontinental pathways of AIV diffusion.

Our findings also suggest that highly divergent lineages may evolve independently in South America and are not found in North America. In addition to the previously described H13 Argentinian lineage [16], the newly identified Peruvian H13 virus contains an internal gene belonging to a South American lineage raising questions about how extensively this lineage circulates in Latin America. Our demonstration of some Peruvian AIV segments falling into ‘global lineages’ which are defined by host (migratory gulls and shorebirds) rather than geographic region is consistent with extensive geographic mosaicism in gull AIV seen in North American studies [17,18] and highlights the important of such hosts in the evolution of AIV diversity in the Americas.

Why certain South American viruses do not appear among the intensively sampled wild bird populations of North America remains a mystery, given the frequency of North-South bird migration corridors between these continents each year and the fact that this lineage be widespread in South America, based on its identification in multiple countries. These major outstanding questions highlight the short-sightedness of studying AIV ecology in North America without knowledge of the full ecological context of AIV throughout the Americas. The recent incursion of HPAI H5 viruses into North America has made knowledge of the spatial dynamics of AIVs in the Americas all the more important [5]. Although our analysis and prior studies have identified many diverse AIV lineages of North American and potentially Eurasian origin in South American wild birds, the frequency of viral introduction and the risk of an incursion of H5 into South America remain difficult to assess, owing to the relative lack of data from the region.

## Supporting Information

S1 Fig Locations used for avian fecal sampling, AIV detection and AIV whole genome sequencing. (PDF)

S2 Fig Phylogenetic relationships of the PB2 segment of AIVs in Peru. Maximum likelihood phylogeny inferred for 2,190 PB2 segments of AIVs collected globally, including those collected from wild birds in Peru. Labels and shading are similar to Figs 1 and 2, including individual black circles or clusters of overlapping black circles for putative viral introduction events into Peru. Numbers in brackets indicate bootstrap support for each clade of viruses representing a single introduction into Peru. Scale bar indicates number of nucleotide substitutions per site.
(PDF)

S3 Fig Phylogenetic relationships of the PB1 segment of AIVs in South America. Maximum likelihood phylogeny inferred for 2,137 PB1 segments of AIVs collected globally, including those collected from wild birds in Peru. Labels and shading are similar to Figs 1 and 2, including individual black circles or clusters of overlapping black circles for putative viral introduction events into Peru. Numbers in brackets indicate bootstrap support for each clade of viruses representing a single introduction into Peru. Scale bar indicates number of nucleotide substitutions per site.
(PDF)

S4 Fig Phylogenetic relationships of the NP segment of AIVs in South America. Maximum likelihood phylogeny inferred for 2,107 NP segments of AIVs collected globally, including those collected from wild birds in Peru. Labels and shading are similar to Figs 1 and 2, including individual black circles or clusters of overlapping black circles for putative viral introduction events into Peru. Numbers in brackets indicate bootstrap support for each clade of viruses representing a single introduction into Peru. Scale bar indicates number of nucleotide substitutions per site.
(PDF)

S5 Fig Phylogenetic relationships of the MP segment of AIVs in South America. Maximum likelihood phylogeny inferred for 2,111 MP segments of AIVs collected globally, including those collected from wild birds in Peru. Labels and shading are similar to Figs 1 and 2, including individual black circles or clusters of overlapping black circles for putative viral introduction events into Peru. Numbers in brackets indicate bootstrap support for each clade of viruses representing a single introduction into Peru. Scale bar indicates number of nucleotide substitutions per site.
(PDF)

S6 Fig Phylogenetic relationships of the NS segment of AIVs in South America. Maximum likelihood phylogeny inferred for 2,099 NS segments of AIVs collected globally, including those collected from wild birds in Peru. Labels and shading are similar to Figs 1 and 2, including individual black circles or clusters of overlapping black circles for putative viral introduction events into Peru. Numbers in brackets indicate bootstrap support for each clade of viruses representing a single introduction into Peru. Alleles A and B are indicated. Scale bar indicates number of nucleotide substitutions per site.
(PDF)

S7 Fig Phylogenetic relationships of the PB2 segment of AIVs in Peru. Identical tree as S2 Fig, only tip labels have been indicated and clades are expanded in full. Lineages are colored as per Figs 1 and 2, S2–S6 Figs. Peruvian sequence tips are indicated in red. Viral introduction events into Peru are indicated with a blue circle. All bootstrap values are shown. Scale bar indicates number of nucleotide substitutions per site.
(PDF)

S8 Fig Phylogenetic relationships of the PB1 segment of AIVs in Peru. Identical tree as S3 Fig, only tip labels have been indicated and clades are expanded in full. Lineages are colored as per Figs 1 and 2, S2–S6 Figs. Peruvian sequence tips are indicated in red. Viral introduction events into Peru are indicated with a blue circle. All bootstrap values are shown. Scale bar indicates number of nucleotide substitutions per site.
(PDF)

S9 Fig Phylogenetic relationships of the PA segment of AIVs in Peru. Identical tree as Fig 2, only tip labels have been indicated and clades are expanded in full. Lineages are colored as per Figs 1 and 2, S2–S6 Figs. Peruvian sequence tips are indicated in red. Viral introduction events into Peru are indicated with a blue circle. All bootstrap values are shown. Scale bar indicates number of nucleotide substitutions per site.
(PDF)

S10 Fig Phylogenetic relationships of the NP segment of AIVs in Peru. Identical tree as S4 Fig, only tip labels have been indicated and clades are expanded in full. Lineages are colored as per Figs 1 and 2, S2–S6 Figs. Peruvian sequence tips are indicated in red. Viral introduction events into Peru are indicated with a blue circle. All bootstrap values are shown. Scale bar indicates number of nucleotide substitutions per site.
(PDF)

S11 Fig Phylogenetic relationships of the MP segment of AIVs in Peru. Identical tree as S5 Fig, only tip labels have been indicated and clades are expanded in full. Lineages are colored as per Figs 1 and 2, S2–S6 Figs. Peruvian sequence tips are indicated in red. Viral introduction events into Peru are indicated with a blue circle. All bootstrap values are shown. Scale bar indicates number of nucleotide substitutions per site.
(PDF)

S12 Fig Phylogenetic relationships of the NS segment of AIVs in Peru. Identical tree as S6 Fig, only tip labels have been indicated and clades are expanded in full. Lineages are colored as per Figs 1 and 2, S2–S6 Figs. Peruvian sequence tips are indicated in red. Viral introduction events into Peru are indicated with a blue circle. All bootstrap values are shown. Scale bar indicates number of nucleotide substitutions per site.
(PDF)

S13 Fig Phylogeny of the HA segment of all global H13 AIVs. Maximum likelihood phylogeny inferred for the HA segment of 6 H13 viruses collected for this study from Peru, as well as all global H13 viruses available from GenBank (n = 85). Tip labels are displayed, and Peruvian strains are shaded red. The tree is mid-point rooted for clarity, and all branch lengths are drawn to scale. Peruvian sequences are indicated in red. All bootstrap values are shown. Scale bar indicates number of nucleotide substitutions per site.
(PDF)

S14 Fig Phylogeny of the N2 segment. Neighbor-joining tree inferred for the N2 segment of seven Peruvian viruses (5 H13N2 viruses, 1 H3N2 virus, and 1 H10N2 virus), as well as for 221 N2 viruses collected from the Americas, including the Argentinian H6N2 and H9N2 viruses. Tip labels are displayed, and Peruvian strains are shaded red. The tree is mid-point rooted for clarity, and all branch lengths are drawn to scale. Peruvian sequence tips are indicated in red. All bootstrap values are shown. Scale bar indicates number of nucleotide substitutions per site.
(PDF)

S1 Table AIVs from Peru sequenced for this study. (DOCX)

S2 Table Primers used in RT-PCR amplification of influenza A genome segments. (DOCX)

S3 Table Reference AIV sequences from GenBank used in this study. (DOCX)

S4 Table Reference H13 sequences from GenBank used in this study. (DOCX)

S5 Table Viral introductions observed during each year from 2006–2010. (DOCX)
