# Supplementary figures and images for: The Genetic Diversity of Influenza A Viruses in Wild Birds in Peru
Source: PLoS One. 2016 Jan 19;11(1):e0146059. doi: 10.1371/journal.pone.0146059 (PMC4718589; doi:10.1371/journal.pone.0146059)

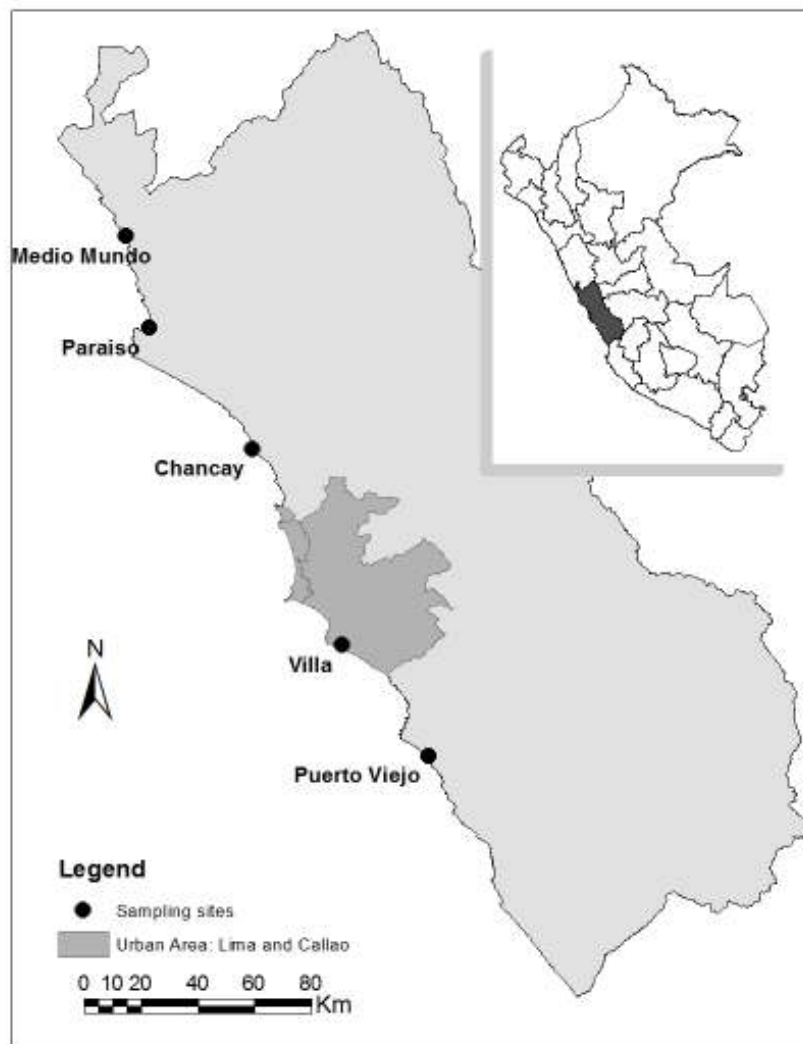

Supplement: S1 Fig — (PDF) [file pone.0146059.s001.pdf]

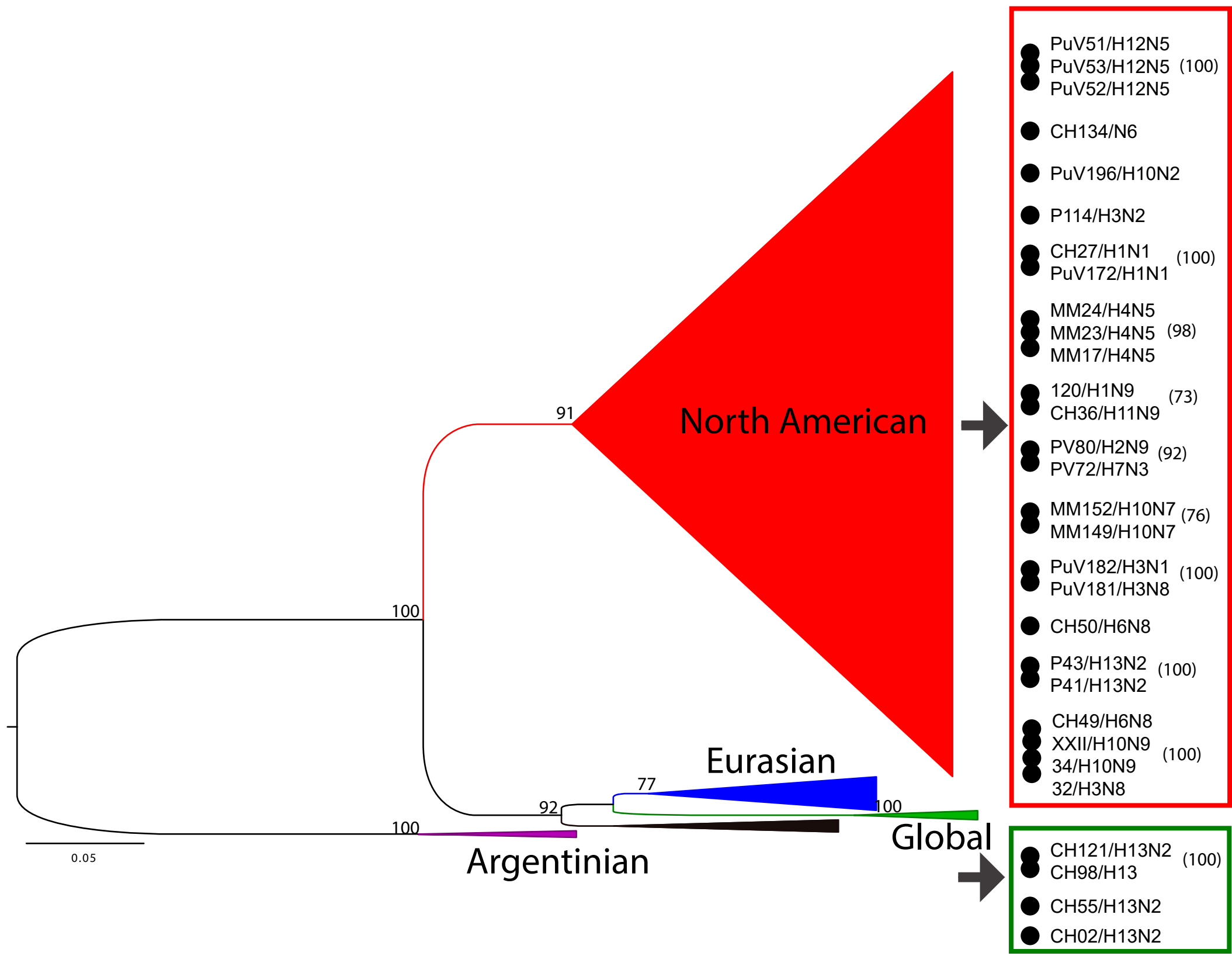

Supplement: S2 Fig — Maximum likelihood phylogeny inferred for 2,190 PB2 segments of AIVs collected globally, including those collected from wild birds in Peru. Labels and shading are similar to Figs 1 and 2, including individual black circles or clusters of overlapping black circles for putative viral introduction events into Peru. Numbers in brackets indicate bootstrap support for each clade of viruses representing a single introduction into Peru. Scale bar indicates number of nucleotide substitutions per site. (PDF) [file pone.0146059.s002.pdf]

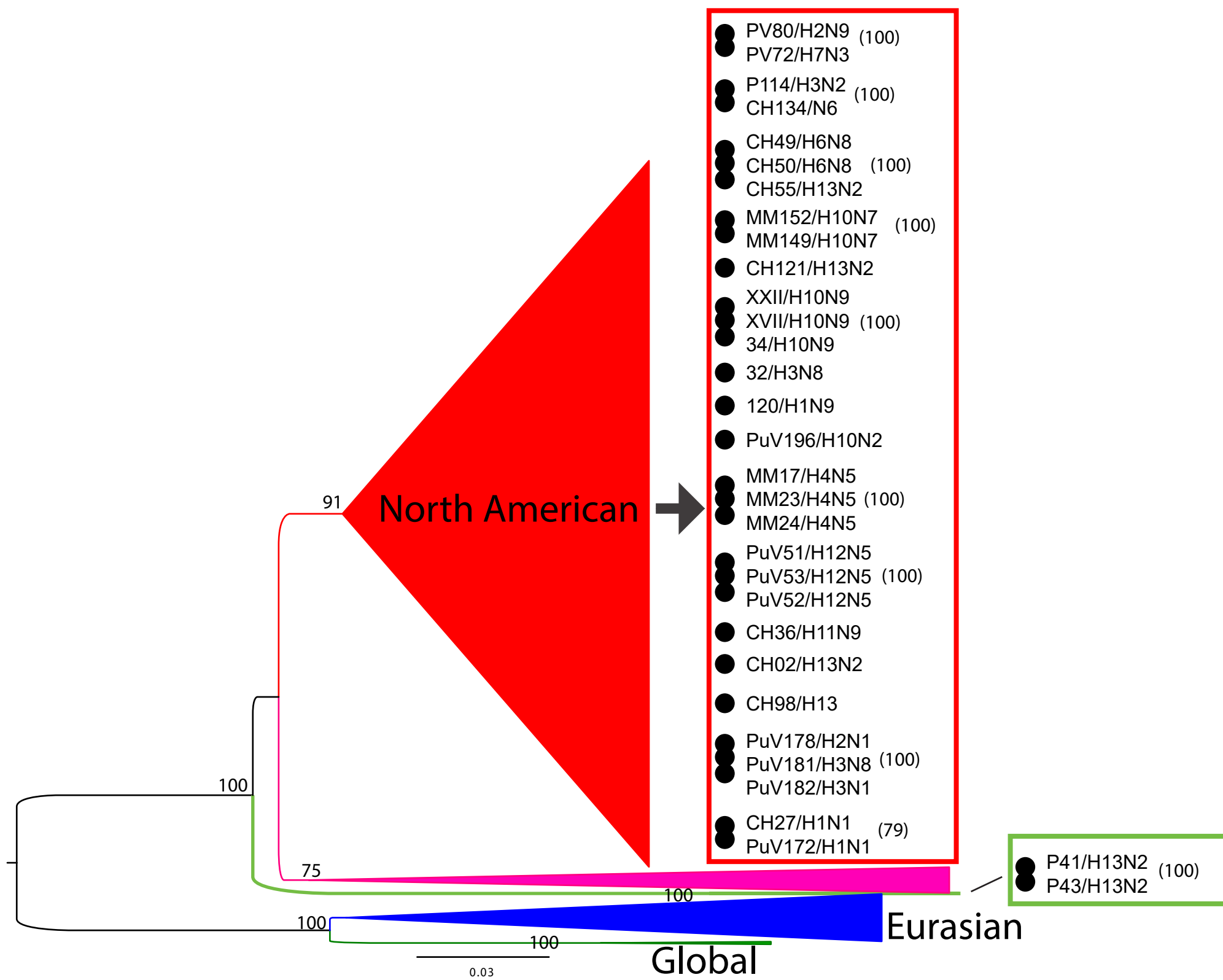

Supplement: S3 Fig — Maximum likelihood phylogeny inferred for 2,137 PB1 segments of AIVs collected globally, including those collected from wild birds in Peru. Labels and shading are similar to Figs 1 and 2, including individual black circles or clusters of overlapping black circles for putative viral introduction events into Peru. Numbers in brackets indicate bootstrap support for each clade of viruses representing a single introduction into Peru. Scale bar indicates number of nucleotide substitutions per site. (PDF) [file pone.0146059.s003.pdf]

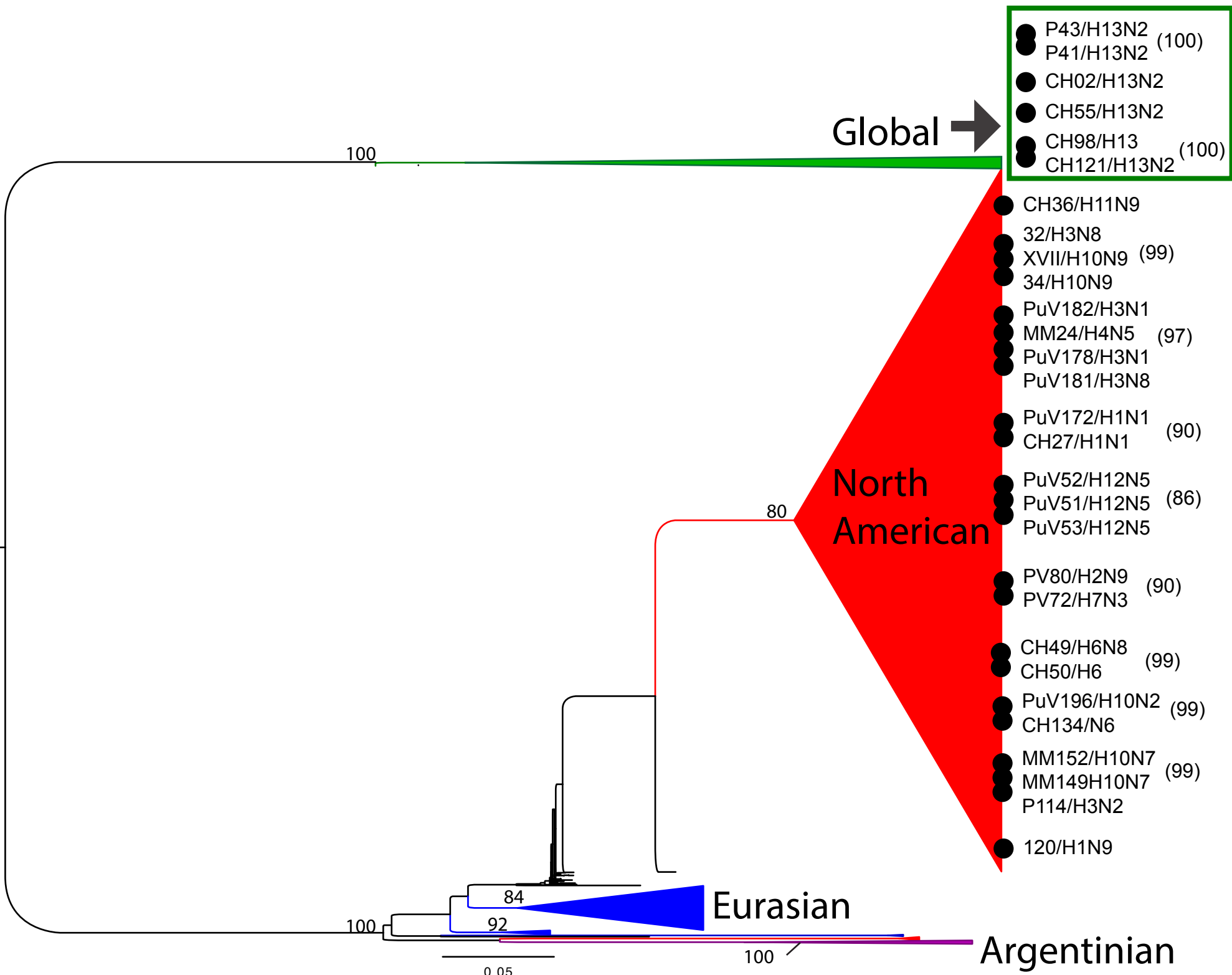

Supplement: S4 Fig — Maximum likelihood phylogeny inferred for 2,107 NP segments of AIVs collected globally, including those collected from wild birds in Peru. Labels and shading are similar to Figs 1 and 2, including individual black circles or clusters of overlapping black circles for putative viral introduction events into Peru. Numbers in brackets indicate bootstrap support for each clade of viruses representing a single introduction into Peru. Scale bar indicates number of nucleotide substitutions per site. (PDF) [file pone.0146059.s004.pdf]

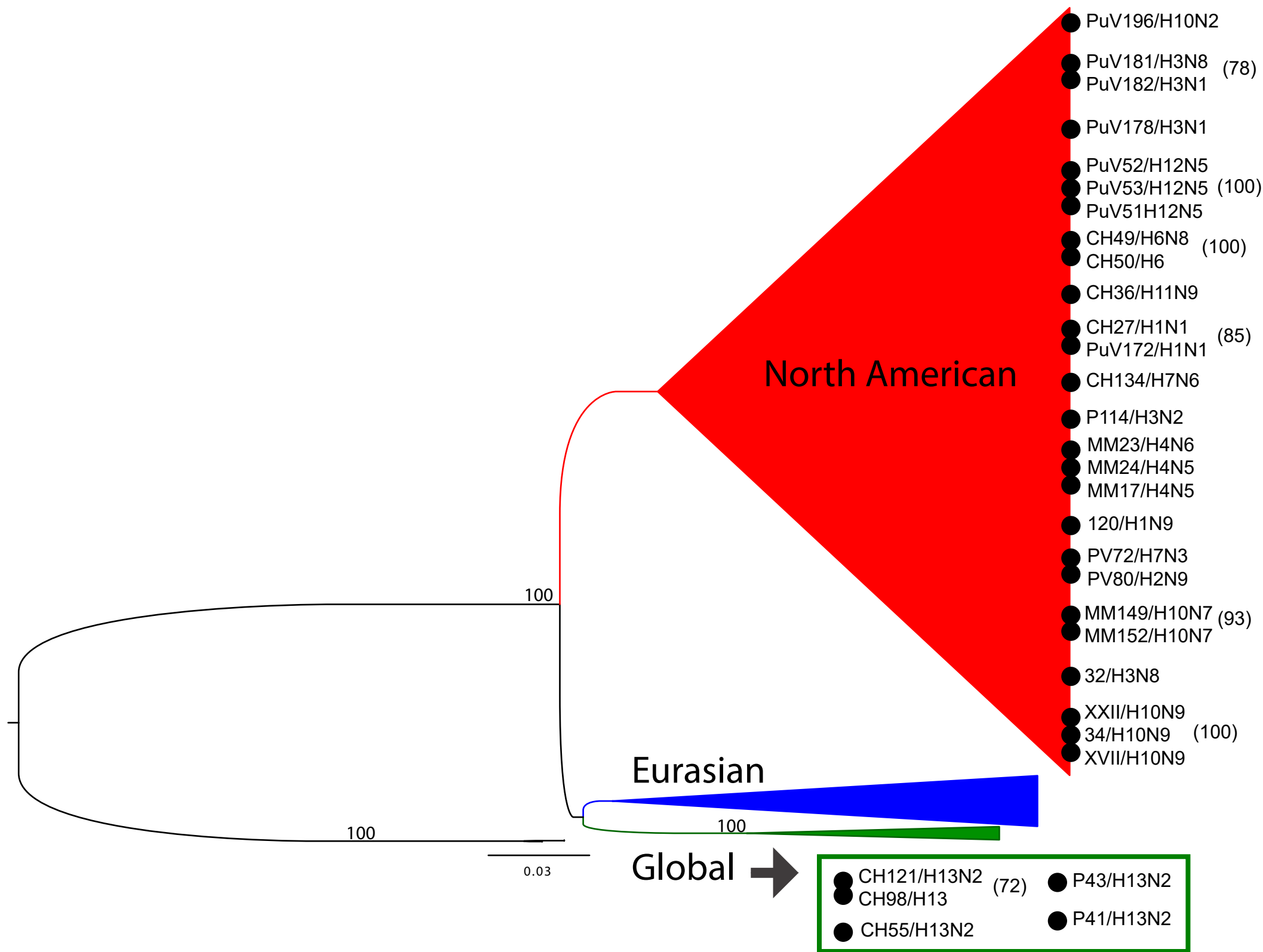

Supplement: S5 Fig — Maximum likelihood phylogeny inferred for 2,111 MP segments of AIVs collected globally, including those collected from wild birds in Peru. Labels and shading are similar to Figs 1 and 2, including individual black circles or clusters of overlapping black circles for putative viral introduction events into Peru. Numbers in brackets indicate bootstrap support for each clade of viruses representing a single introduction into Peru. Scale bar indicates number of nucleotide substitutions per site. (PDF) [file pone.0146059.s005.pdf]

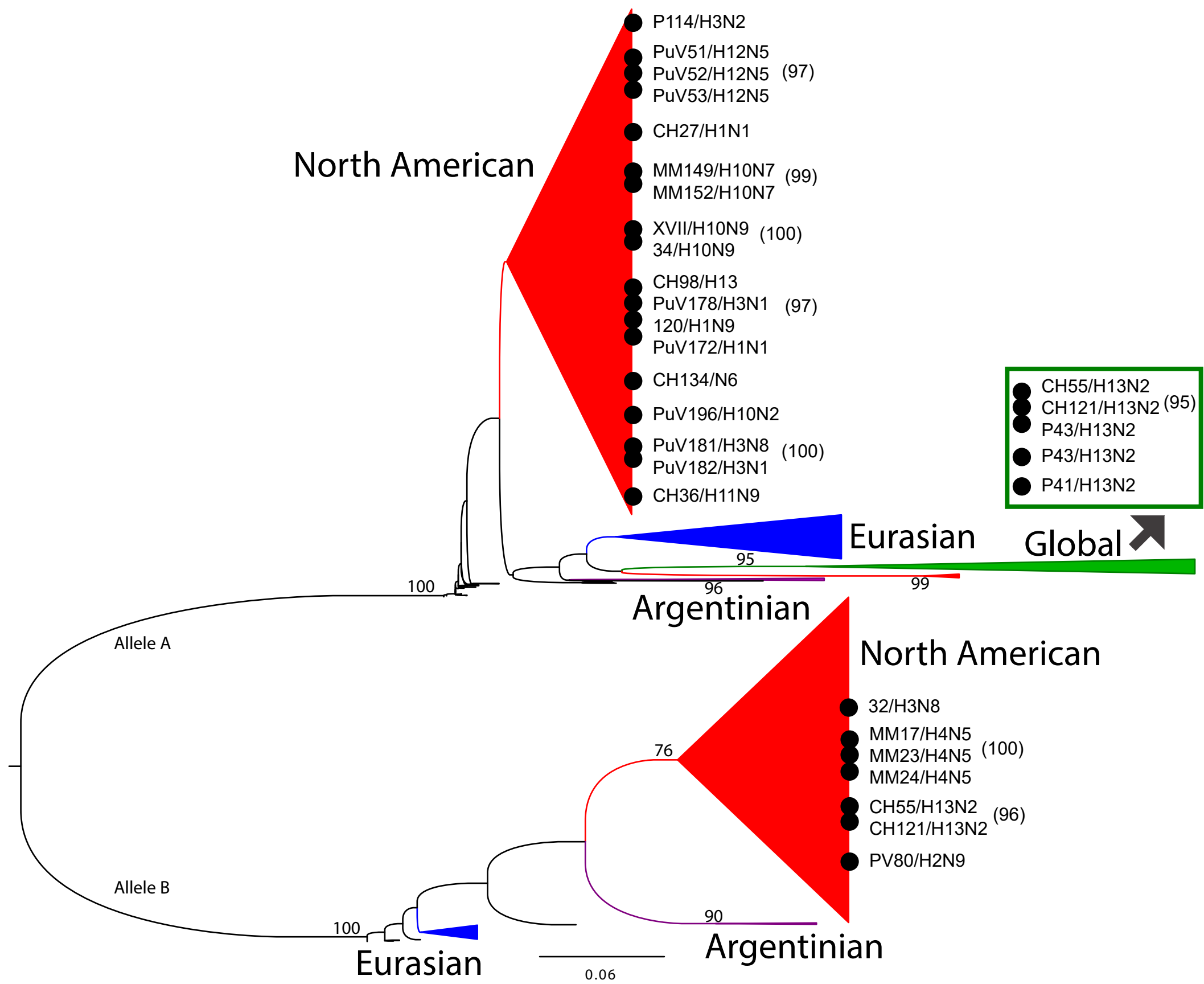

Supplement: S6 Fig — Maximum likelihood phylogeny inferred for 2,099 NS segments of AIVs collected globally, including those collected from wild birds in Peru. Labels and shading are similar to Figs 1 and 2, including individual black circles or clusters of overlapping black circles for putative viral introduction events into Peru. Numbers in brackets indicate bootstrap support for each clade of viruses representing a single introduction into Peru. Alleles A and B are indicated. Scale bar indicates number of nucleotide substitutions per site. (PDF) [file pone.0146059.s006.pdf]

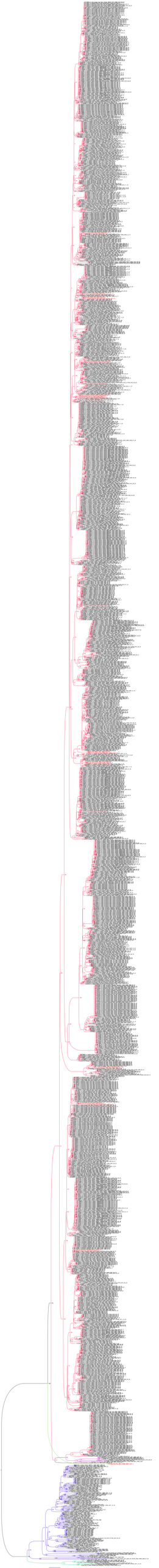

Supplement: S8 Fig — Identical tree as S3 Fig, only tip labels have been indicated and clades are expanded in full. Lineages are colored as per Figs 1 and 2, S2–S6 Figs. Peruvian sequence tips are indicated in red. Viral introduction events into Peru are indicated with a blue circle. All bootstrap values are shown. Scale bar indicates number of nucleotide substitutions per site. (PDF) [file pone.0146059.s008.pdf]

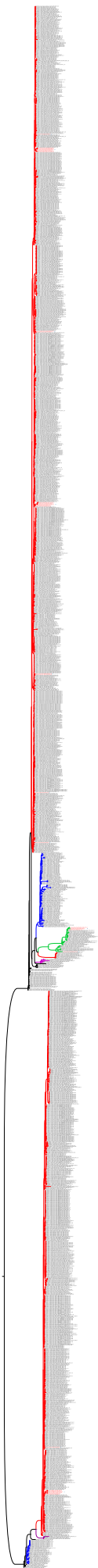

Supplement: S12 Fig — Identical tree as S6 Fig, only tip labels have been indicated and clades are expanded in full. Lineages are colored as per Figs 1 and 2, S2–S6 Figs. Peruvian sequence tips are indicated in red. Viral introduction events into Peru are indicated with a blue circle. All bootstrap values are shown. Scale bar indicates number of nucleotide substitutions per site. (PDF) [file pone.0146059.s012.pdf]

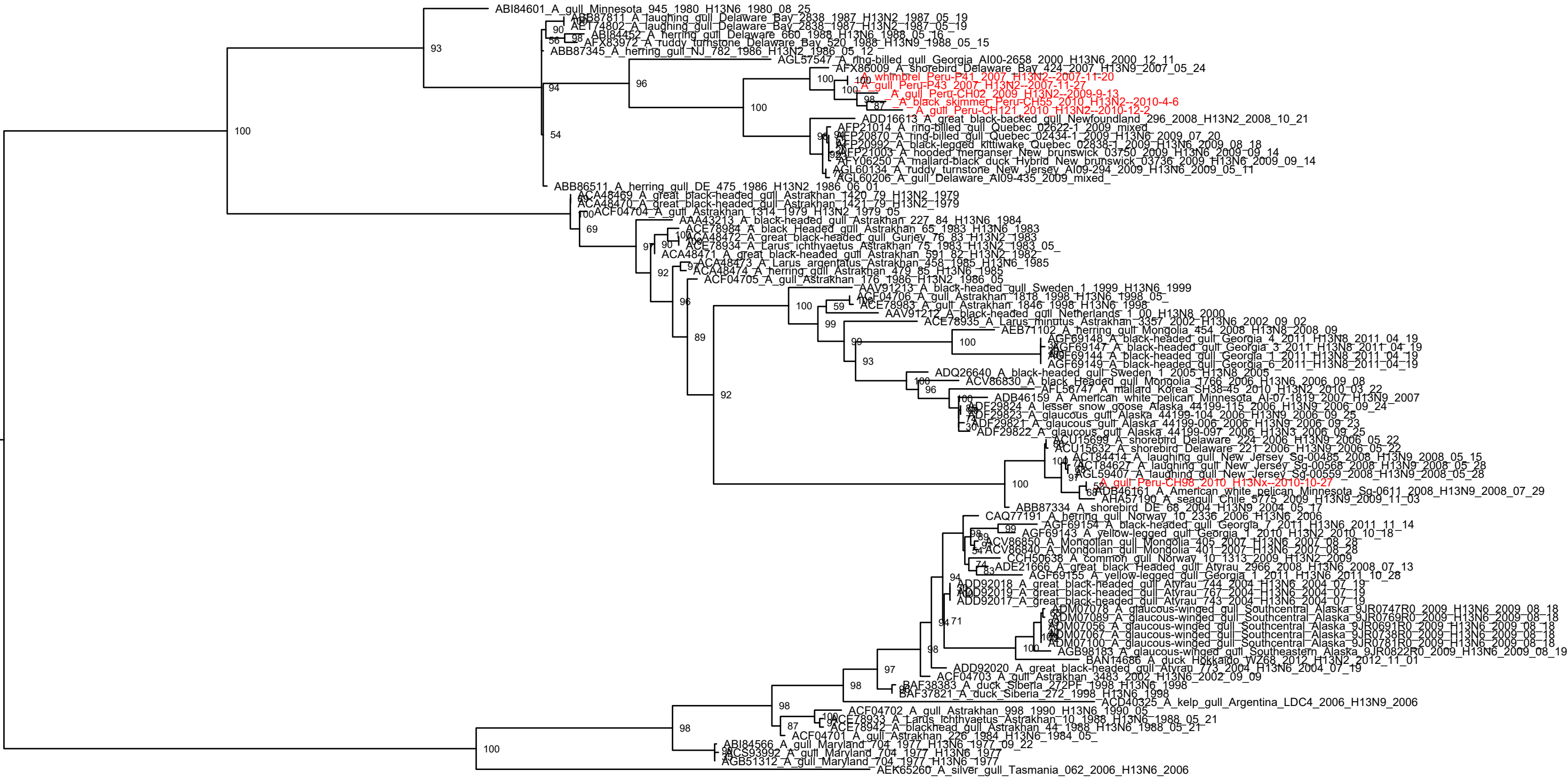

0.04

Supplement: S13 Fig — Maximum likelihood phylogeny inferred for the HA segment of 6 H13 viruses collected for this study from Peru, as well as all global H13 viruses available from GenBank (n = 85). Tip labels are displayed, and Peruvian strains are shaded red. The tree is mid-point rooted for clarity, and all branch lengths are drawn to scale. Peruvian sequences are indicated in red. All bootstrap values are shown. Scale bar indicates number of nucleotide substitutions per site. (PDF) [file pone.0146059.s013.pdf]

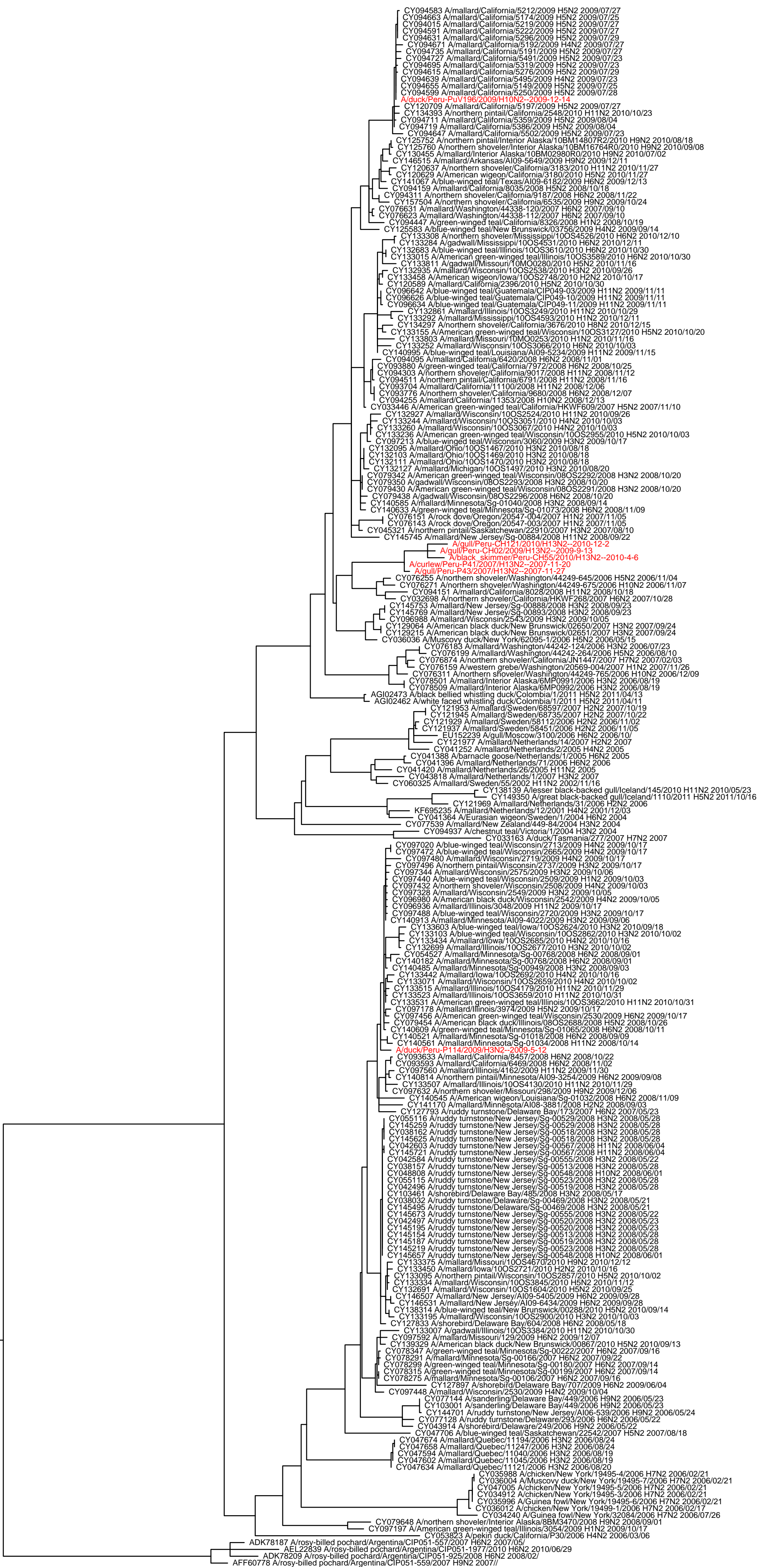

Supplement: S14 Fig — Neighbor-joining tree inferred for the N2 segment of seven Peruvian viruses (5 H13N2 viruses, 1 H3N2 virus, and 1 H10N2 virus), as well as for 221 N2 viruses collected from the Americas, including the Argentinian H6N2 and H9N2 viruses. Tip labels are displayed, and Peruvian strains are shaded red. The tree is mid-point rooted for clarity, and all branch lengths are drawn to scale. Peruvian sequence tips are indicated in red. All bootstrap values are shown. Scale bar indicates number of nucleotide substitutions per site. (PDF) [file pone.0146059.s014.pdf]
