# Supplementary material for: The Genetic Diversity of Influenza A Viruses in Wild Birds in Peru
Source: PLoS One. 2016 Jan 19;11(1):e0146059. doi: 10.1371/journal.pone.0146059 (PMC4718589; doi:10.1371/journal.pone.0146059)
Supplement: S1 Table — (DOCX) [file pone.0146059.s015.docx]

| **Table S1. AIVs from Peru sequenced for this study** | | | | | | |
| --- | --- | --- | --- | --- | --- | --- |
| **Virus name** | **Location** | **Collection date** | **Host** | **Serotype** | **Segment** | **Accession Number** |
| Influenza A virus (A/whimbrel /Peru/CH27/2009(H1N1)) | Peru: Chancay | 18-Nov-2009 | Whimbrel | H1N1 | 7 | KR824536 |
| Influenza A virus (A/whimbrel /Peru/CH27/2009(H1N1)) | Peru: Chancay | 18-Nov-2009 | Whimbrel | H1N1 | 6 | KR824537 |
| Influenza A virus (A/whimbrel /Peru/CH27/2009(H1N1)) | Peru: Chancay | 18-Nov-2009 | Whimbrel | H1N1 | 5 | KR824538 |
| Influenza A virus (A/whimbrel /Peru/CH27/2009(H1N1)) | Peru: Chancay | 18-Nov-2009 | Whimbrel | H1N1 | 8 | KR824539 |
| Influenza A virus (A/whimbrel /Peru/CH27/2009(H1N1)) | Peru: Chancay | 18-Nov-2009 | Whimbrel | H1N1 | 3 | KR824540 |
| Influenza A virus (A/whimbrel /Peru/CH27/2009(H1N1)) | Peru: Chancay | 18-Nov-2009 | Whimbrel | H1N1 | 2 | KR824541 |
| Influenza A virus (A/whimbrel /Peru/CH27/2009(H1N1)) | Peru: Chancay | 18-Nov-2009 | Whimbrel | H1N1 | 1 | KR824542 |
| Influenza A virus (A/moorhen/Peru/120/2009(H1N9)) | Peru: Paraiso | 19-May-2009 | Moorhen | H1N9 | 4 | KR824543 |
| Influenza A virus (A/moorhen/Peru/120/2009(H1N9)) | Peru: Paraiso | 19-May-2009 | Moorhen | H1N9 | 7 | KR824544 |
| Influenza A virus (A/moorhen/Peru/120/2009(H1N9)) | Peru: Paraiso | 19-May-2009 | Moorhen | H1N9 | 6 | KR824545 |
| Influenza A virus (A/moorhen/Peru/120/2009(H1N9)) | Peru: Paraiso | 19-May-2009 | Moorhen | H1N9 | 5 | KR824546 |
| Influenza A virus (A/moorhen/Peru/120/2009(H1N9)) | Peru: Paraiso | 19-May-2009 | Moorhen | H1N9 | 8 | KR824547 |
| Influenza A virus (A/moorhen/Peru/120/2009(H1N9)) | Peru: Paraiso | 19-May-2009 | Moorhen | H1N9 | 3 | KR824548 |
| Influenza A virus (A/moorhen/Peru/120/2009(H1N9)) | Peru: Paraiso | 19-May-2009 | Moorhen | H1N9 | 2 | KR824549 |
| Influenza A virus (A/moorhen/Peru/120/2009(H1N9)) | Peru: Paraiso | 19-May-2009 | Moorhen | H1N9 | 1 | KR824550 |
| Influenza A virus (A/oystercatcher/Peru/34/2006 (H10N9)) | Peru: Puerto Viejo | 07-Nov-2006 | Oyster catcher | H10N9 | 4 | KR824551 |
| Influenza A virus (A/oystercatcher/Peru/34/2006 (H10N9)) | Peru: Puerto Viejo | 07-Nov-2006 | Oyster catcher | H10N9 | 7 | KR824552 |
| Influenza A virus (A/oystercatcher/Peru/34/2006 (H10N9)) | Peru: Puerto Viejo | 07-Nov-2006 | Oyster catcher | H10N9 | 6 | KR824553 |
| Influenza A virus (A/oystercatcher/Peru/34/2006 (H10N9)) | Peru: Puerto Viejo | 07-Nov-2006 | Oyster catcher | H10N9 | 5 | KR824554 |
| Influenza A virus (A/oystercatcher/Peru/34/2006 (H10N9)) | Peru: Puerto Viejo | 07-Nov-2006 | Oyster catcher | H10N9 | 8 | KR824555 |
| Influenza A virus (A/oystercatcher/Peru/34/2006 (H10N9)) | Peru: Puerto Viejo | 07-Nov-2006 | Oyster catcher | H10N9 | 2 | KR824556 |
| Influenza A virus (A/oystercatcher/Peru/34/2006 (H10N9)) | Peru: Puerto Viejo | 07-Nov-2006 | Oyster catcher | H10N9 | 1 | KR824557 |
| Influenza A virus (A/gull/Peru/CH02/2009 (H13N2)) | Peru: Chancay | 13-Sept-2009 | Gull | H13N2 | 4 | KR824558 |
| Influenza A virus (A/gull/Peru/CH02/2009 (H13N2)) | Peru: Chancay | 13-Sept-2009 | Gull | H13N2 | 7 | KR824559 |
| Influenza A virus (A/gull/Peru/CH02/2009 (H13N2)) | Peru: Chancay | 13-Sept-2009 | Gull | H13N2 | 6 | KR824560 |
| Influenza A virus (A/gull/Peru/CH02/2009 (H13N2)) | Peru: Chancay | 13-Sept-2009 | Gull | H13N2 | 5 | KR824561 |
| Influenza A virus (A/gull/Peru/CH02/2009 (H13N2)) | Peru: Chancay | 13-Sept-2009 | Gull | H13N2 | 8 | KR824562 |
| Influenza A virus (A/gull/Peru/CH02/2009 (H13N2)) | Peru: Chancay | 13-Sept-2009 | Gull | H13N2 | 3 | KR824563 |
| Influenza A virus (A/gull/Peru/CH02/2009 (H13N2)) | Peru: Chancay | 13-Sept-2009 | Gull | H13N2 | 2 | KR824564 |
| Influenza A virus (A/gull/Peru/CH02/2009 (H13N2)) | Peru: Chancay | 13-Sep-2009 | Gull | H13N2 | 1 | KR824565 |
| Influenza A virus (A/gull/Peru/CH121/2010 (H13N2)) | Peru: Chancay | 02-Dec-2010 | Gull | H13N2 | 4 | KR824566 |
| Influenza A virus (A/gull/Peru/CH121/2010 (H13N2)) | Peru: Chancay | 02-Dec-2010 | Gull | H13N2 | 7 | KR824567 |
| Influenza A virus (A/gull/Peru/CH121/2010 (H13N2)) | Peru: Chancay | 02-Dec-2010 | Gull | H13N2 | 6 | KR824568 |
| Influenza A virus (A/gull/Peru/CH121/2010 (H13N2)) | Peru: Chancay | 02-Dec-2010 | Gull | H13N2 | 5 | KR824569 |
| Influenza A virus (A/gull/Peru/CH121/2010 (H13N2)) | Peru: Chancay | 02-Dec-2010 | Gull | H13N2 | 8 | KR824570 |
| Influenza A virus (A/gull/Peru/CH121/2010 (H13N2)) | Peru: Chancay | 02-Dec-2010 | Gull | H13N2 | 3 | KR824571 |
| Influenza A virus (A/gull/Peru/CH121/2010 (H13N2)) | Peru: Chancay | 02-Dec-2010 | Gull | H13N2 | 2 | KR824572 |
| Influenza A virus (A/gull/Peru/CH121/2010 (H13N2)) | Peru: Chancay | 02-Dec-2010 | Gull | H13N2 | 1 | KR824573 |
| Influenza A virus (A/gull/Peru/CH134/2010 (H?N6)) | Peru: Chancay | 27-Nov-2010 | Gull | H?N6 | 7 | KR824574 |
| Influenza A virus (A/gull/Peru/CH134/2010 (H?N6)) | Peru: Chancay | 27-Nov-2010 | Gull | H?N6 | 6 | KR824575 |
| Influenza A virus (A/gull/Peru/CH134/2010 (H?N6)) | Peru: Chancay | 27-Nov-2010 | Gull | H?N6 | 5 | KR824576 |
| Influenza A virus (A/gull/Peru/CH134/2010 (H?N6)) | Peru: Chancay | 27-Nov-2010 | Gull | H?N6 | 8 | KR824577 |
| Influenza A virus (A/gull/Peru/CH134/2010 (H?N6)) | Peru: Chancay | 27-Nov-2010 | Gull | H?N6 | 4 | KR824578 |
| Influenza A virus (A/gull/Peru/CH134/2010 (H?N6)) | Peru: Chancay | 27-Nov-2010 | Gull | H?N6 | 2 | KR824579 |
| Influenza A virus (A/gull/Peru/CH134/2010 (H?N6)) | Peru: Chancay | 27-Nov-2010 | Gull | H?N6 | 1 | KR824580 |
| Influenza A virus (A/duck/Peru/CH36/2010 (H11N9)) | Peru: Chancay | 09-Mar-2010 | Duck | H11N9 | 4 | KR824581 |
| Influenza A virus (A/duck/Peru/CH36/2010 (H11N9)) | Peru: Chancay | 09-Mar-2010 | Duck | H11N9 | 7 | KR824582 |
| Influenza A virus (A/duck/Peru/CH36/2010 (H11N9)) | Peru: Chancay | 09-Mar-2010 | Duck | H11N9 | 5 | KR824583 |
| Influenza A virus (A/duck/Peru/CH36/2010 (H11N9)) | Peru: Chancay | 09-Mar-2010 | Duck | H11N9 | 8 | KR824584 |
| Influenza A virus (A/duck/Peru/CH36/2010 (H11N9)) | Peru: Chancay | 09-Mar-2010 | Duck | H11N9 | 3 | KR824585 |
| Influenza A virus (A/duck/Peru/CH36/2010 (H11N9)) | Peru: Chancay | 09-Mar-2010 | Duck | H11N9 | 2 | KR824586 |
| Influenza A virus (A/duck/Peru/CH36/2010 (H11N9)) | Peru: Chancay | 09-Mar-2010 | Duck | H11N9 | 1 | KR824587 |
| Influenza A virus (A/willet/Peru-CH49/2010 (H6N8)) | Peru: Chancay | 2010 | Willet | H6N8 | 4 | KR824588 |
| Influenza A virus (A/willet/Peru-CH49/2010 (H6N8)) | Peru: Chancay | 2010 | Willet | H6N8 | 7 | KR824589 |
| Influenza A virus (A/willet/Peru-CH49/2010 (H6N8)) | Peru: Chancay | 2010 | Willet | H6N8 | 6 | KR824590 |
| Influenza A virus (A/willet/Peru-CH49/2010 (H6N8)) | Peru: Chancay | 2010 | Willet | H6N8 | 5 | KR824591 |
| Influenza A virus (A/willet/Peru-CH49/2010 (H6N8)) | Peru: Chancay | 2010 | Willet | H6N8 | 8 | KR824592 |
| Influenza A virus (A/willet/Peru-CH49/2010 (H6N8)) | Peru: Chancay | 2010 | Willet | H6N8 | 3 | KR824593 |
| Influenza A virus (A/willet/Peru-CH49/2010 (H6N8)) | Peru: Chancay | 2010 | Willet | H6N8 | 2 | KR824594 |
| Influenza A virus (A/willet/Peru-CH49/2010 (H6N8)) | Peru: Chancay | 2010 | Willet | H6N8 | 1 | KR824595 |
| Influenza A virus (A/egret/Peru-CH50/2010 (H6N8)) | Peru: Chancay | 25-Mar-2010 | Egret | H6N8 | 4 | KR824596 |
| Influenza A virus (A/egret/Peru-CH50/2010 (H6N8)) | Peru: Chancay | 25-Mar-2010 | Egret | H6N8 | 7 | KR824597 |
| Influenza A virus (A/egret/Peru-CH50/2010 (H6N8)) | Peru: Chancay | 25-Mar-2010 | Egret | H6N8 | 6 | KR824598 |
| Influenza A virus (A/egret/Peru-CH50/2010 (H6N8)) | Peru: Chancay | 25-Mar-2010 | Egret | H6N8 | 5 | KR824599 |
| Influenza A virus (A/egret/Peru-CH50/2010 (H6N8)) | Peru: Chancay | 25-Mar-2010 | Egret | H6N8 | 8 | KR824600 |
| Influenza A virus (A/egret/Peru-CH50/2010 (H6N8)) | Peru: Chancay | 25-Mar-2010 | Egret | H6N8 | 3 | KR824601 |
| Influenza A virus (A/egret/Peru-CH50/2010 (H6N8)) | Peru: Chancay | 25-Mar-2010 | Egret | H6N8 | 2 | KR824602 |
| Influenza A virus (A/egret/Peru-CH50/2010 (H6N8)) | Peru: Chancay | 25-Mar-2010 | Egret | H6N8 | 1 | KR824603 |
| Influenza A virus (A/black skimmer/Peru/CH55/2010 (H13N2)) | Peru: Chancay | 06-Apr-2010 | Black skimmer | H13N2 | 4 | KR824604 |
| Influenza A virus (A/black skimmer/Peru/CH55/2010 (H13N2)) | Peru: Chancay | 06-Apr-2010 | Black skimmer | H13N2 | 7 | KR824605 |
| Influenza A virus (A/black skimmer/Peru/CH55/2010 (H13N2)) | Peru: Chancay | 06-Apr-2010 | Black skimmer | H13N2 | 6 | KR824606 |
| Influenza A virus (A/black skimmer/Peru/CH55/2010 (H13N2)) | Peru: Chancay | 06-Apr-2010 | Black skimmer | H13N2 | 5 | KR824607 |
| Influenza A virus (A/black skimmer/Peru/CH55/2010 (H13N2)) | Peru: Chancay | 06-Apr-2010 | Black skimmer | H13N2 | 8 | KR824608 |
| Influenza A virus (A/black skimmer/Peru/CH55/2010 (H13N2)) | Peru: Chancay | 06-Apr-2010 | Black skimmer | H13N2 | 3 | KR824609 |
| Influenza A virus (A/black skimmer/Peru/CH55/2010 (H13N2)) | Peru: Chancay | 06-Apr-2010 | Black skimmer | H13N2 | 2 | KR824610 |
| Influenza A virus (A/black skimmer/Peru/CH55/2010 (H13N2)) | Peru: Chancay | 06-Apr-2010 | Black skimmer | H13N2 | 1 | KR824611 |
| Influenza A virus (A/gull/Peru/CH98/2010 (H13N?)) | Peru: Chancay | 27-Nov-2010 | Gull | H13N? | 4 | KR824612 |
| Influenza A virus (A/gull/Peru/CH98/2010 (H13N?)) | Peru: Chancay | 27-Nov-2010 | Gull | H13N? | 7 | KR824613 |
| Influenza A virus (A/gull/Peru/CH98/2010 (H13N?)) | Peru: Chancay | 27-Nov-2010 | Gull | H13N? | 5 | KR824614 |
| Influenza A virus (A/gull/Peru/CH98/2010 (H13N?)) | Peru: Chancay | 27-Nov-2010 | Gull | H13N? | 8 | KR824615 |
| Influenza A virus (A/gull/Peru/CH98/2010 (H13N?)) | Peru: Chancay | 27-Nov-2010 | Gull | H13N? | 3 | KR824616 |
| Influenza A virus (A/gull/Peru/CH98/2010 (H13N?)) | Peru: Chancay | 27-Nov-2010 | Gull | H13N? | 2 | KR824617 |
| Influenza A virus (A/gull/Peru/CH98/2010 (H13N?)) | Peru: Chancay | 27-Nov-2010 | Gull | H13N? | 1 | KR824618 |
| Influenza A virus (A/ruddy turnstone/Peru/MM149/2008 (H10N7)) | Peru: Medio Mundo | 29-Oct-2008 | Ruddy turnstone | H10N7 | 4 | KR824619 |
| Influenza A virus (A/ruddy turnstone/Peru/MM149/2008 (H10N7)) | Peru: Medio Mundo | 29-Oct-2008 | Ruddy turnstone | H10N7 | 7 | KR824620 |
| Influenza A virus (A/ruddy turnstone/Peru/MM149/2008 (H10N7)) | Peru: Medio Mundo | 29-Oct-2008 | Ruddy turnstone | H10N7 | 5 | KR824621 |
| Influenza A virus (A/ruddy turnstone/Peru/MM149/2008 (H10N7)) | Peru: Medio Mundo | 29-Oct-2008 | Ruddy turnstone | H10N7 | 8 | KR824622 |
| Influenza A virus (A/ruddy turnstone/Peru/MM149/2008 (H10N7)) | Peru: Medio Mundo | 29-Oct-2008 | Ruddy turnstone | H10N7 | 3 | KR824623 |
| Influenza A virus (A/ruddy turnstone/Peru/MM149/2008 (H10N7)) | Peru: Medio Mundo | 29-Oct-2008 | Ruddy turnstone | H10N7 | 2 | KR824624 |
| Influenza A virus (A/ruddy turnstone/Peru/MM149/2008 (H10N7)) | Peru: Medio Mundo | 29-Oct-2008 | Ruddy turnstone | H10N7 | 1 | KR824625 |
| Influenza A virus (A/oystercatcher/Peru/MM152/2008 (H10N7)) | Peru: Medio Mundo | 05-Nov-2008 | Oyster catcher | H10N7 | 4 | KR824626 |
| Influenza A virus (A/oystercatcher/Peru/MM152/2008 (H10N7)) | Peru: Medio Mundo | 05-Nov-2008 | Oyster catcher | H10N7 | 7 | KR824627 |
| Influenza A virus (A/oystercatcher/Peru/MM152/2008 (H10N7)) | Peru: Medio Mundo | 05-Nov-2008 | Oyster catcher | H10N7 | 6 | KR824628 |
| Influenza A virus (A/oystercatcher/Peru/MM152/2008 (H10N7)) | Peru: Medio Mundo | 05-Nov-2008 | Oyster catcher | H10N7 | 5 | KR824629 |
| Influenza A virus (A/oystercatcher/Peru/MM152/2008 (H10N7)) | Peru: Medio Mundo | 05-Nov-2008 | Oyster catcher | H10N7 | 8 | KR824630 |
| Influenza A virus (A/oystercatcher/Peru/MM152/2008 (H10N7)) | Peru: Medio Mundo | 05-Nov-2008 | Oyster catcher | H10N7 | 2 | KR824631 |
| Influenza A virus (A/oystercatcher/Peru/MM152/2008 (H10N7)) | Peru: Medio Mundo | 05-Nov-2008 | Oyster catcher | H10N7 | 1 | KR824632 |
| Influenza A virus (A/duck/Peru/MM17/2007(H4N5)) | Peru: Medio Mundo | 06-Feb-2007 | Duck | H4N5 | 4 | KR824633 |
| Influenza A virus (A/duck/Peru/MM17/2007(H4N5)) | Peru: Medio Mundo | 06-Feb-2007 | Duck | H4N5 | 7 | KR824634 |
| Influenza A virus (A/duck/Peru/MM17/2007(H4N5)) | Peru: Medio Mundo | 06-Feb-2007 | Duck | H4N5 | 6 | KR824635 |
| Influenza A virus (A/duck/Peru/MM17/2007(H4N5)) | Peru: Medio Mundo | 06-Feb-2007 | Duck | H4N5 | 5 | KR824636 |
| Influenza A virus (A/duck/Peru/MM17/2007(H4N5)) | Peru: Medio Mundo | 06-Feb-2007 | Duck | H4N5 | 8 | KR824637 |
| Influenza A virus (A/duck/Peru/MM17/2007(H4N5)) | Peru: Medio Mundo | 06-Feb-2007 | Duck | H4N5 | 3 | KR824638 |
| Influenza A virus (A/duck/Peru/MM17/2007(H4N5)) | Peru: Medio Mundo | 06-Feb-2007 | Duck | H4N5 | 2 | KR824639 |
| Influenza A virus (A/duck/Peru/MM17/2007(H4N5)) | Peru: Medio Mundo | 06-Feb-2007 | Duck | H4N5 | 1 | KR824640 |
| Influenza A virus (A/duck/Peru-MM23/2007 (H4N5)) | Peru: Medio Mundo | 13-Feb-2007 | Duck | H4N5 | 7 | KR824641 |
| Influenza A virus (A/duck/Peru-MM23/2007 (H4N5)) | Peru: Medio Mundo | 13-Feb-2007 | Duck | H4N5 | 6 | KR824642 |
| Influenza A virus (A/duck/Peru-MM23/2007 (H4N5)) | Peru: Medio Mundo | 13-Feb-2007 | Duck | H4N5 | 5 | KR824643 |
| Influenza A virus (A/duck/Peru-MM23/2007 (H4N5)) | Peru: Medio Mundo | 13-Feb-2007 | Duck | H4N5 | 8 | KR824644 |
| Influenza A virus (A/duck/Peru-MM23/2007 (H4N5)) | Peru: Medio Mundo | 13-Feb-2007 | Duck | H4N5 | 3 | KR824645 |
| Influenza A virus (A/duck/Peru-MM23/2007 (H4N5)) | Peru: Medio Mundo | 13-Feb-2007 | Duck | H4N5 | 2 | KR824646 |
| Influenza A virus (A/duck/Peru-MM23/2007 (H4N5)) | Peru: Medio Mundo | 13-Feb-2007 | Duck | H4N5 | 1 | KR824647 |
| Influenza A virus (A/pelican/Peru-MM24/2007 (H4N5)) | Peru: Medio Mundo | 13-Feb-2007 | Pelican | H4N5 | 4 | KR824648 |
| Influenza A virus (A/pelican/Peru-MM24/2007 (H4N5)) | Peru: Medio Mundo | 13-Feb-2007 | Pelican | H4N5 | 7 | KR824649 |
| Influenza A virus (A/pelican/Peru-MM24/2007 (H4N5)) | Peru: Medio Mundo | 13-Feb-2007 | Pelican | H4N5 | 6 | KR824650 |
| Influenza A virus (A/pelican/Peru-MM24/2007 (H4N5)) | Peru: Medio Mundo | 13-Feb-2007 | Pelican | H4N5 | 5 | KR824651 |
| Influenza A virus (A/pelican/Peru-MM24/2007 (H4N5)) | Peru: Medio Mundo | 13-Feb-2007 | Pelican | H4N5 | 8 | KR824652 |
| Influenza A virus (A/pelican/Peru-MM24/2007 (H4N5)) | Peru: Medio Mundo | 13-Feb-2007 | Pelican | H4N5 | 2 | KR824653 |
| Influenza A virus (A/pelican/Peru-MM24/2007 (H4N5)) | Peru: Medio Mundo | 13-Feb-2007 | Pelican | H4N5 | 1 | KR824654 |
| Influenza A virus (A/duck/Peru/P114/2009(H3N2)) | Peru: Paraiso | 12-May-2009 | Duck | H3N2 | 4 | KR824655 |
| Influenza A virus (A/duck/Peru/P114/2009(H3N2)) | Peru: Paraiso | 12-May-2009 | Duck | H3N2 | 7 | KR824656 |
| Influenza A virus (A/duck/Peru/P114/2009(H3N2)) | Peru: Paraiso | 12-May-2009 | Duck | H3N2 | 6 | KR824657 |
| Influenza A virus (A/duck/Peru/P114/2009(H3N2)) | Peru: Paraiso | 12-May-2009 | Duck | H3N2 | 5 | KR824658 |
| Influenza A virus (A/duck/Peru/P114/2009(H3N2)) | Peru: Paraiso | 12-May-2009 | Duck | H3N2 | 8 | KR824659 |
| Influenza A virus (A/duck/Peru/P114/2009(H3N2)) | Peru: Paraiso | 12-May-2009 | Duck | H3N2 | 3 | KR824660 |
| Influenza A virus (A/duck/Peru/P114/2009(H3N2)) | Peru: Paraiso | 12-May-2009 | Duck | H3N2 | 2 | KR824661 |
| Influenza A virus (A/duck/Peru/P114/2009(H3N2)) | Peru: Paraiso | 12-May-2009 | Duck | H3N2 | 1 | KR824662 |
| Influenza A virus (A/whimbrel/Peru/P41/2007 (H13N2)) | Peru: Paraiso | 20-Nov-2007 | Whimbrel | H13N2 | 4 | KR824663 |
| Influenza A virus (A/whimbrel/Peru/P41/2007 (H13N2)) | Peru: Paraiso | 20-Nov-2007 | Whimbrel | H13N2 | 7 | KR824664 |
| Influenza A virus (A/whimbrel/Peru/P41/2007 (H13N2)) | Peru: Paraiso | 20-Nov-2007 | Whimbrel | H13N2 | 6 | KR824665 |
| Influenza A virus (A/whimbrel/Peru/P41/2007 (H13N2)) | Peru: Paraiso | 20-Nov-2007 | Whimbrel | H13N2 | 5 | KR824666 |
| Influenza A virus (A/whimbrel/Peru/P41/2007 (H13N2)) | Peru: Paraiso | 20-Nov-2007 | Whimbrel | H13N2 | 8 | KR824667 |
| Influenza A virus (A/whimbrel/Peru/P41/2007 (H13N2)) | Peru: Paraiso | 20-Nov-2007 | Whimbrel | H13N2 | 3 | KR824668 |
| Influenza A virus (A/whimbrel/Peru/P41/2007 (H13N2)) | Peru: Paraiso | 20-Nov-2007 | Whimbrel | H13N2 | 2 | KR824669 |
| Influenza A virus (A/whimbrel/Peru/P41/2007 (H13N2)) | Peru: Paraiso | 20-Nov-2007 | Whimbrel | H13N2 | 1 | KR824670 |
| Influenza A virus (A/gull/Peru/P43/2007 (H13N2)) | Peru: Paraiso | 27-Nov-2007 | Gull | H13N2 | 4 | KR824671 |
| Influenza A virus (A/gull/Peru/P43/2007 (H13N2)) | Peru: Paraiso | 27-Nov-2007 | Gull | H13N2 | 7 | KR824672 |
| Influenza A virus (A/gull/Peru/P43/2007 (H13N2)) | Peru: Paraiso | 27-Nov-2007 | Gull | H13N2 | 6 | KR824673 |
| Influenza A virus (A/gull/Peru/P43/2007 (H13N2)) | Peru: Paraiso | 27-Nov-2007 | Gull | H13N2 | 5 | KR824674 |
| Influenza A virus (A/gull/Peru/P43/2007 (H13N2)) | Peru: Paraiso | 27-Nov-2007 | Gull | H13N2 | 8 | KR824675 |
| Influenza A virus (A/gull/Peru/P43/2007 (H13N2)) | Peru: Paraiso | 27-Nov-2007 | Gull | H13N2 | 3 | KR824676 |
| Influenza A virus (A/gull/Peru/P43/2007 (H13N2)) | Peru: Paraiso | 27-Nov-2007 | Gull | H13N2 | 2 | KR824677 |
| Influenza A virus (A/gull/Peru/P43/2007 (H13N2)) | Peru: Paraiso | 27-Nov-2007 | Gull | H13N2 | 1 | KR824678 |
| Influenza A virus (A/curlew/Peru/PuV178/2009(H3N1)) | Peru: Puerto Viejo | 24-Nov-2009 | Curlew | H3N1 | 4 | KR824679 |
| Influenza A virus (A/curlew/Peru/PuV178/2009(H3N1)) | Peru: Puerto Viejo | 24-Nov-2009 | Curlew | H3N1 | 7 | KR824680 |
| Influenza A virus (A/curlew/Peru/PuV178/2009(H3N1)) | Peru: Puerto Viejo | 24-Nov-2009 | Curlew | H3N1 | 6 | KR824681 |
| Influenza A virus (A/curlew/Peru/PuV178/2009(H3N1)) | Peru: Puerto Viejo | 24-Nov-2009 | Curlew | H3N1 | 5 | KR824682 |
| Influenza A virus (A/curlew/Peru/PuV178/2009(H3N1)) | Peru: Puerto Viejo | 24-Nov-2009 | Curlew | H3N1 | 8 | KR824683 |
| Influenza A virus (A/curlew/Peru/PuV178/2009(H3N1)) | Peru: Puerto Viejo | 24-Nov-2009 | Curlew | H3N1 | 3 | KR824684 |
| Influenza A virus (A/curlew/Peru/PuV178/2009(H3N1)) | Peru: Puerto Viejo | 24-Nov-2009 | Curlew | H3N1 | 2 | KR824685 |
| Influenza A virus: (A/ruddy turnstone /Peru/PuV181/2009(H3N1)) | Peru: Puerto Viejo | 24-Nov-2009 | Ruddy turnstone | H3N12 | 4 | KR824686 |
| Influenza A virus: (A/ruddy turnstone /Peru/PuV181/2009(H3N1)) | Peru: Puerto Viejo | 24-Nov-2009 | Ruddy turnstone | H3N1 | 7 | KR824687 |
| Influenza A virus: (A/ruddy turnstone /Peru/PuV181/2009(H3N1)) | Peru: Puerto Viejo | 24-Nov-2009 | Ruddy turnstone | H3N1 | 6 | KR824688 |
| Influenza A virus: (A/ruddy turnstone /Peru/PuV181/2009(H3N1)) | Peru: Puerto Viejo | 24-Nov-2009 | Ruddy turnstone | H3N1 | 5 | KR824689 |
| Influenza A virus: (A/ruddy turnstone /Peru/PuV181/2009(H3N1)) | Peru: Puerto Viejo | 24-Nov-2009 | Ruddy turnstone | H3N1 | 8 | KR824690 |
| Influenza A virus: (A/ruddy turnstone /Peru/PuV181/2009(H3N1)) | Peru: Puerto Viejo | 24-Nov-2009 | Ruddy turnstone | H3N1 | 3 | KR824691 |
| Influenza A virus: (A/ruddy turnstone /Peru/PuV181/2009(H3N1)) | Peru: Puerto Viejo | 24-Nov-2009 | Ruddy turnstone | H3N1 | 2 | KR824692 |
| Influenza A virus: (A/ruddy turnstone /Peru/PuV181/2009(H3N1)) | Peru: Puerto Viejo | 24-Nov-2009 | Ruddy turnstone | H3N1 | 1 | KR824693 |
| Influenza A virus: (A/ruddy turnstone /Peru/PuV182/2009(H3N1)) | Peru: Puerto Viejo | 24-Nov-2009 | Ruddy turnstone | H3N1 | 4 | KR824694 |
| Influenza A virus: (A/ruddy turnstone /Peru/PuV182/2009(H3N1)) | Peru: Puerto Viejo | 24-Nov-2009 | Ruddy turnstone | H3N1 | 7 | KR824695 |
| Influenza A virus: (A/ruddy turnstone /Peru/PuV182/2009(H3N1)) | Peru: Puerto Viejo | 24-Nov-2009 | Ruddy turnstone | H3N1 | 6 | KR824696 |
| Influenza A virus: (A/ruddy turnstone /Peru/PuV182/2009(H3N1)) | Peru: Puerto Viejo | 24-Nov-2009 | Ruddy turnstone | H3N1 | 5 | KR824697 |
| Influenza A virus: (A/ruddy turnstone /Peru/PuV182/2009(H3N1)) | Peru: Puerto Viejo | 24-Nov-2009 | Ruddy turnstone | H3N1 | 8 | KR824698 |
| Influenza A virus: (A/ruddy turnstone /Peru/PuV182/2009(H3N1)) | Peru: Puerto Viejo | 24-Nov-2009 | Ruddy turnstone | H3N1 | 3 | KR824699 |
| Influenza A virus: (A/ruddy turnstone /Peru/PuV182/2009(H3N1)) | Peru: Puerto Viejo | 24-Nov-2009 | Ruddy turnstone | H3N1 | 2 | KR824700 |
| Influenza A virus: (A/ruddy turnstone /Peru/PuV182/2009(H3N1)) | Peru: Puerto Viejo | 24-Nov-2009 | Ruddy turnstone | H3N1 | 1 | KR824701 |
| Influenza A virus (A/duck/Peru-PuV196/2009 (H10N2)) | Peru: Puerto Viejo | 14-Dec-2009 | Duck | H10N2 | 4 | KR824765 |
| Influenza A virus (A/duck/Peru-PuV196/2009 (H10N2)) | Peru: Puerto Viejo | 14-Dec-2009 | Duck | H10N2 | 7 | KR824702 |
| Influenza A virus (A/duck/Peru-PuV196/2009 (H10N2)) | Peru: Puerto Viejo | 14-Dec-2009 | Duck | H10N2 | 6 | KR824703 |
| Influenza A virus (A/duck/Peru-PuV196/2009 (H10N2)) | Peru: Puerto Viejo | 14-Dec-2009 | Duck | H10N2 | 5 | KR824704 |
| Influenza A virus (A/duck/Peru-PuV196/2009 (H10N2)) | Peru: Puerto Viejo | 14-Dec-2009 | Duck | H10N2 | 8 | KR824705 |
| Influenza A virus (A/duck/Peru-PuV196/2009 (H10N2)) | Peru: Puerto Viejo | 14-Dec-2009 | Duck | H10N2 | 2 | KR824706 |
| Influenza A virus (A/duck/Peru-PuV196/2009 (H10N2)) | Peru: Puerto Viejo | 14-Dec-2009 | Duck | H10N2 | 1 | KR824707 |
| Influenza A virus (A/duck/Peru/32/2006 (H3N8)) | Peru: Puerto Viejo | 06-Nov-2006 | Duck | H3N8 | 7 | KR824708 |
| Influenza A virus (A/duck/Peru/32/2006 (H3N8)) | Peru: Puerto Viejo | 06-Nov-2006 | Duck | H3N8 | 5 | KR824709 |
| Influenza A virus (A/duck/Peru/32/2006 (H3N8)) | Peru: Puerto Viejo | 06-Nov-2006 | Duck | H3N8 | 8 | KR824710 |
| Influenza A virus (A/duck/Peru/32/2006 (H3N8)) | Peru: Puerto Viejo | 06-Nov-2006 | Duck | H3N8 | 9 | KR824711 |
| Influenza A virus (A/duck/Peru/32/2006 (H3N8)) | Peru: Puerto Viejo | 06-Nov-2006 | Duck | H3N8 | 10 | KR824712 |
| Influenza A virus (A/duck/Peru/32/2006 (H3N8)) | Peru: Puerto Viejo | 06-Nov-2006 | Duck | H3N8 | 1 | KR824713 |
| Influenza A virus (A/ruddy turnstone /Peru/PuV51/2008 (H12N5)) | Peru: Puerto Viejo | 22-Apr-2008 | Ruddy turnstone | H12N5 | 4 | KR824714 |
| Influenza A virus (A/ruddy turnstone /Peru/PuV51/2008 (H12N5)) | Peru: Puerto Viejo | 22-Apr-2008 | Ruddy turnstone | H12N5 | 7 | KR824715 |
| Influenza A virus (A/ruddy turnstone /Peru/PuV51/2008 (H12N5)) | Peru: Puerto Viejo | 22-Apr-2008 | Ruddy turnstone | H12N5 | 6 | KR824716 |
| Influenza A virus (A/ruddy turnstone /Peru/PuV51/2008 (H12N5)) | Peru: Puerto Viejo | 22-Apr-2008 | Ruddy turnstone | H12N5 | 5 | KR824717 |
| Influenza A virus (A/ruddy turnstone /Peru/PuV51/2008 (H12N5)) | Peru: Puerto Viejo | 22-Apr-2008 | Ruddy turnstone | H12N5 | 8 | KR824718 |
| Influenza A virus (A/ruddy turnstone /Peru/PuV51/2008 (H12N5)) | Peru: Puerto Viejo | 22-Apr-2008 | Ruddy turnstone | H12N5 | 3 | KR824719 |
| Influenza A virus (A/ruddy turnstone /Peru/PuV51/2008 (H12N5)) | Peru: Puerto Viejo | 22-Apr-2008 | Ruddy turnstone | H12N5 | 2 | KR824720 |
| Influenza A virus (A/ruddy turnstone /Peru/PuV51/2008 (H12N5)) | Peru: Puerto Viejo | 22-Apr-2008 | Ruddy turnstone | H12N5 | 1 | KR824721 |
| Influenza A virus (A/ruddy turnstone /Peru/PuV52/2008 (H12N5)) | Peru: Puerto Viejo | 22-Apr-2008 | Ruddy turnstone | H12N5 | 4 | KR824722 |
| Influenza A virus (A/ruddy turnstone /Peru/PuV52/2008 (H12N5)) | Peru: Puerto Viejo | 22-Apr-2008 | Ruddy turnstone | H12N5 | 7 | KR824723 |
| Influenza A virus (A/ruddy turnstone /Peru/PuV52/2008 (H12N5)) | Peru: Puerto Viejo | 22-Apr-2008 | Ruddy turnstone | H12N5 | 6 | KR824724 |
| Influenza A virus (A/ruddy turnstone /Peru/PuV52/2008 (H12N5)) | Peru: Puerto Viejo | 22-Apr-2008 | Ruddy turnstone | H12N5 | 5 | KR824725 |
| Influenza A virus (A/ruddy turnstone /Peru/PuV52/2008 (H12N5)) | Peru: Puerto Viejo | 22-Apr-2008 | Ruddy turnstone | H12N5 | 8 | KR824726 |
| Influenza A virus (A/ruddy turnstone /Peru/PuV52/2008 (H12N5)) | Peru: Puerto Viejo | 22-Apr-2008 | Ruddy turnstone | H12N5 | 3 | KR824727 |
| Influenza A virus (A/ruddy turnstone /Peru/PuV52/2008 (H12N5)) | Peru: Puerto Viejo | 22-Apr-2008 | Ruddy turnstone | H12N5 | 2 | KR824728 |
| Influenza A virus (A/ruddy turnstone /Peru/PuV52/2008 (H12N5)) | Peru: Puerto Viejo | 22-Apr-2008 | Ruddy turnstone | H12N5 | 1 | KR824729 |
| Influenza A virus (A/duck/Peru-PV72/2008 (H7N3)) | Peru: Pantanos de villa | 15-Jul-2008 | Duck | H7N3 | 2 | KR824730 |
| Influenza A virus (A/duck/Peru-PV72/2008 (H7N3)) | Peru: Pantanos de villa | 15-Jul-2008 | Duck | H7N3 | 7 | KR824731 |
| Influenza A virus (A/duck/Peru-PV72/2008 (H7N3)) | Peru: Pantanos de villa | 15-Jul-2008 | Duck | H7N3 | 6 | KR824732 |
| Influenza A virus (A/duck/Peru-PV72/2008 (H7N3)) | Peru: Pantanos de villa | 15-Jul-2008 | Duck | H7N3 | 5 | KR824733 |
| Influenza A virus (A/duck/Peru-PV72/2008 (H7N3)) | Peru: Pantanos de villa | 15-Jul-2008 | Duck | H7N3 | 8 | KR824734 |
| Influenza A virus (A/duck/Peru-PV72/2008 (H7N3)) | Peru: Pantanos de villa | 15Jul-2008 | Duck | H7N3 | 3 | KR824735 |
| Influenza A virus (A/duck/Peru-PV72/2008 (H7N3)) | Peru: Pantanos de villa | 15-Jul-2008 | Duck | H7N3 | 2 | KR824736 |
| Influenza A virus (A/duck/Peru-PV72/2008 (H7N3)) | Peru: Pantanos de villa | 15-Jul-2008 | Duck | H7N3 | 1 | KR824737 |
| Influenza A virus (A/duck/Peru/PV80/2008(H2N9)) | Peru: Pantanos de villa | 22-Jul-2008 | Duck | H2N9 | 4 | KR824738 |
| Influenza A virus (A/duck/Peru/PV80/2008(H2N9)) | Peru: Pantanos de villa | 22-Jul-2008 | Duck | H2N9 | 3 | KR824739 |
| Influenza A virus (A/duck/Peru/PV80/2008(H2N9)) | Peru: Pantanos de villa | 22-Jul-2008 | Duck | H2N9 | 4 | KR824740 |
| Influenza A virus (A/duck/Peru/PV80/2008(H2N9)) | Peru: Pantanos de villa | 22-Jul-2008 | Duck | H2N9 | 5 | KR824741 |
| Influenza A virus (A/duck/Peru/PV80/2008(H2N9)) | Peru: Pantanos de villa | 22-Jul-2008 | Duck | H2N9 | 6 | KR824742 |
| Influenza A virus (A/duck/Peru/PV80/2008(H2N9)) | Peru: Pantanos de villa | 22-Jul-2008 | Duck | H2N9 | 7 | KR824743 |
| Influenza A virus (A/duck/Peru/PV80/2008(H2N9)) | Peru: Pantanos de villa | 22-Jul-2008 | Duck | H2N9 | 8 | KR824744 |
| Influenza A virus (A/duck/Peru/PV80/2008(H2N9)) | Peru: Pantanos de villa | 22-Jul-2008 | Duck | H2N9 | 1 | KR824745 |
| Influenza A virus (A/ruddy turnstone/Peru/XVII/2006 (H10N9)) | Peru: Puerto Viejo | 17-Oc-2006 | Ruddy turnstone | H10N9 | 4 | KR824746 |
| Influenza A virus (A/ruddy turnstone/Peru/XVII/2006 (H10N9)) | Peru: Puerto Viejo | 17-Oct-2006 | Ruddy turnstone | H10N9 | 7 | KR824747 |
| Influenza A virus (A/ruddy turnstone/Peru/XVII/2006 (H10N9)) | Peru: Puerto Viejo | 17-Oct-2006 | Ruddy turnstone | H10N9 | 6 | KR824748 |
| Influenza A virus (A/ruddy turnstone/Peru/XVII/2006 (H10N9)) | Peru: Puerto Viejo | 17-Oct-2006 | Ruddy turnstone | H10N9 | 5 | KR824749 |
| Influenza A virus (A/ruddy turnstone/Peru/XVII/2006 (H10N9)) | Peru: Puerto Viejo | 17-Oct-2006 | Ruddy turnstone | H10N9 | 8 | KR824750 |
| Influenza A virus (A/ruddy turnstone/Peru/XVII/2006 (H10N9)) | Peru: Puerto Viejo | 17-Oct-2006 | Ruddy turnstone | H10N9 | 2 | KR824751 |
| Influenza A virus (A/ruddy turnstone/Peru/XXII/2006 (H10N9)) | Peru: Puerto Viejo | 24-Oct-2006 | Ruddy turnstone | H10N9 | 4 | KR824752 |
| Influenza A virus (A/ruddy turnstone/Peru/XXII/2006 (H10N9)) | Peru: Puerto Viejo | 24-Oct-2006 | Ruddy turnstone | H10N9 | 6 | KR824753 |
| Influenza A virus (A/ruddy turnstone/Peru/XXII/2006 (H10N9)) | Peru: Puerto Viejo | 24-Oct-2006 | Ruddy turnstone | H10N9 | 5 | KR824754 |
| Influenza A virus (A/ruddy turnstone/Peru/XXII/2006 (H10N9)) | Peru: Puerto Viejo | 24-Oct-2006 | Ruddy turnstone | H10N9 | 2 | KR824755 |
| Influenza A virus (A/ruddy turnstone/Peru/XXII/2006 (H10N9)) | Peru: Puerto Viejo | 24-Oct-2006 | Ruddy turnstone | H10N9 | 1 | KR824756 |
| Influenza A virus (A/gull/Peru/PuV172/2009(H1N1)) | Peru: Puerto Viejo | 10-Nov-2009 | Gull | H1N1 | 4 | KR824757 |
| Influenza A virus (A/gull/Peru/PuV172/2009(H1N1)) | Peru: Puerto Viejo | 10-Nov-2009 | Gull | H1N1 | 5 | KR824758 |
| Influenza A virus (A/gull/Peru/PuV172/2009(H1N1)) | Peru: Puerto Viejo | 10-Nov-2009 | Gull | H1N1 | 8 | KR824759 |
| Influenza A virus (A/gull/Peru/PuV172/2009(H1N1)) | Peru: Puerto Viejo | 10-Nov-2009 | Gull | H1N1 | 2 | KR824760 |
| Influenza A virus (A/gull/Peru/PuV172/2009(H1N1)) | Peru: Puerto Viejo | 10-Nov-2009 | Gull | H1N1 | 1 | KR824761 |
| Influenza A virus (A/gull/Peru/PuV172/2009(H1N1)) | Peru: Puerto Viejo | 10-Nov-2009 | Gull | H1N1 | 7 | KR824762 |
| Influenza A virus (A/gull/Peru/PuV172/2009(H1N1)) | Peru: Puerto Viejo | 10-Nov-2009 | Gull | H1N1 | 6 | KR824763 |
| Influenza A virus (A/gull/Peru/PuV172/2009(H1N1)) | Peru: Puerto Viejo | 10-Nov-2009 | Gull | H1N1 | 3 | KR824764 |
