# Supplementary material for: The Genetic Diversity of Influenza A Viruses in Wild Birds in Peru
Source: PLoS One. 2016 Jan 19;11(1):e0146059. doi: 10.1371/journal.pone.0146059 (PMC4718589; doi:10.1371/journal.pone.0146059)
Supplement: S2 Table — (DOCX) [file pone.0146059.s016.docx]

**Table S2. Primers used in RT-PCR amplification of influenza A genome segments**

| **GENE** | ID | **SEQUENCE (5’ to 3’)** | **SIZE (bp)** |
| --- | --- | --- | --- |
| A/MP | M-1 | AGC AAA AGC AGG TAG ATA TT | 1027 |
|  | M-1027R | AGT AGA AAC AAG GTA GTT TTT |  |
| A/NP | NP-1 | AGC AAA AGC AGG GTA GAT AA | 1565 |
|  | NP-1565R | AGT AGA AAC AAG GGT ATT TTT |  |
| A/PA | PA-1 | AGC RAA AGC AGG TAC TGA TYC GAA ATG | 2233 |
|  | PA-2233R | AGT AGA AAC AAG GTA CTT TTT TGG ACA |  |
| A/PB1 | PB1-1 | AGC RAA AGC AGG CAA ACC ATT TGA ATG | 2341 |
|  | PB1-2341R | AGT AGA AAC AAG GCA TTT TTT CAT GAA |  |
| A/PB2 | PB2-1 | AGC RAA AGC AGG TCA ATT ATA TTC A | 2341 |
|  | PB2-2341R | AGT AGA AAC AAG GTC GTT TTT AAA CTA |  |
| A/HA | HA1-1 | AGC AAA AGC AGG GGA AAA TA | 1778 |
|  | HA-1778 | AGT AGA AAC AAG GGT GTT TT |  |
| A/NS | NS-1 | AGC AAA AGC AGG GTG ACA AA | 890 |
|  | NS-890R | AGT AGA AAC AAG GGT GTT TT |  |
| A/NA | NA-1 | AGC AAA AGC AGG AGT GAA AA | 1413 |
|  | NA-1413R | AGT AGA AAC AAG GAG TTT TTT |  |
