# Supplementary material for: The Genetic Diversity of Influenza A Viruses in Wild Birds in Peru
Source: PLoS One. 2016 Jan 19;11(1):e0146059. doi: 10.1371/journal.pone.0146059 (PMC4718589; doi:10.1371/journal.pone.0146059)
Supplement: S3 Table — (DOCX) [file pone.0146059.s017.docx]

**Table S3. AIVs from GenBank used in this study**

| Virus name | Subtype | Date | Accession | Region |
| --- | --- | --- | --- | --- |
| A_chestnut_teal_Victoria_1_2004 | H3N2 | 2004 | CY094942 | Austral-Pacific |
| A_duck_Tasmania_277_2007 | H7N2 | 2007 | CY033168 | Austral-Pacific |
| A_duck_Victoria_512_2007 | H7N6 | 2007 | CY061617 | Austral-Pacific |
| A_duck_Victoria_5384_2002 | H4N8 | 2002 | CY028258 | Austral-Pacific |
| A_red-necked_stint_Australia_2_2004 | H4N8 | 2004 | CY028266 | Austral-Pacific |
| A_red-necked_stint_Australia_3_2004 | H4N8 | 2004 | CY034749 | Austral-Pacific |
| A_sharp-tailed_sandpiper_Australia_10_2004 | H11N9 | 2004 | CY029888 | Austral-Pacific |
| A_sharp-tailed_sandpiper_Australia_6_2004 | H11N9 | 2004 | CY025204 | Austral-Pacific |
| A_Anas_platyrhynchos_Belgium_12827_2007 | H3N8 | 9/18/07 | HM570065 | Eurasian |
| A_Anas_platyrhynchos_Belgium_12827_2007 | H3N8 | 9/18/07 | HM570065 | Eurasian |
| A_barnacle_goose_Netherlands_1_2005 | H6N2 | 2005 | CY041393 | Eurasian |
| A_Bewicks_swan_Netherlands_1_2007 | H1N5 | 2007 | CY076983 | Eurasian |
| A_black_Headed_gull_Mongolia_1756_2006 | H16N3 | 9/8/06 | GQ907301 | Eurasian |
| A_black_Headed_gull_Mongolia_1756_2006 | H16N3 | 9/8/06 | GQ907301 | Eurasian |
| A_black_Headed_gull_Mongolia_1766_2006 | H13N6 | 9/8/06 | GQ907309 | Eurasian |
| A_black_Headed_gull_Mongolia_1766_2006 | H13N6 | 9/8/06 | GQ907309 | Eurasian |
| A_black-headed_gull_Iceland_1298_2011 | H10N5 | 10/23/11 | CY149491 | Eurasian |
| A_black-headed_gull_Iceland_1298_2011 | H10N5 | 10/23/11 | CY149491 | Eurasian |
| A_black-headed_gull_Iceland_713_2010 | H16N3 | 9/13/10 | CY138152 | Eurasian |
| A_black-headed_gull_Netherlands_1_2005 | H6N8 | 2005 | CY041385 | Eurasian |
| A_black-headed_gull_Netherlands_1_2006 | H4N5 | 2006 | CY076999 | Eurasian |
| A_black-headed_gull_Sweden_1_2005 | H13N8 | 2005 | CY077007 | Eurasian |
| A_brown-headed_gull_Qinghai_19_2009 | H5N1 | 2009/06/ | GU477558 | Eurasian |
| A_brown-headed_gull_Qinghai_19_2009 | H5N1 | 2009/06/ | GU477558 | Eurasian |
| A_brown-headed_gull_Qinghai_9_2009 | H5N1 | 2009/06/ | GU477557 | Eurasian |
| A_brown-headed_gull_Qinghai_9_2009 | H5N1 | 2009/06/ | GU477557 | Eurasian |
| A_common_eider_Netherlands_1_2006 | H3N8 | 2006 | CY041345 | Eurasian |
| A_common_eider_Netherlands2_2006 | H4N8 | 2006 | CY076920 | Eurasian |
| A_common_teal_Netherlands_2_2005 | H6N8 | 2005 | CY041377 | Eurasian |
| A_common_teal_Netherlands_2_2008 | H2N3 | 2008 | CY122291 | Eurasian |
| A_common_teal_Netherlands_9_2000 | H11N9 | 11/27/00 | CY060187 | Eurasian |
| A_dunlin_Sweden_1_2005 | H4N6 | 2005 | CY076991 | Eurasian |
| A_Eurasian_wigeon_Netherlands_4_2007 | H10N1 | 2007 | CY077055 | Eurasian |
| A_Eurasian_wigeon_Sweden_1_2004 | H6N2 | 2004 | CY041369 | Eurasian |
| A_glaucous_gull_Iceland_1382_2011 | H2N5 | 10/25/11 | CY149403 | Eurasian |
| A_glaucous_gull_Iceland_1444_2011 | H2N5 | 10/29/11 | CY149443 | Eurasian |
| A_glaucous_gull-herring_gull_Hybrid_Iceland_1108_2011 | H16N3 | 10/16/11 | CY149347 | Eurasian |
| A_great_black-backed_gull_Iceland_1110_2011 | H5N2 | 10/16/11 | CY149355 | Eurasian |
| A_great_black-backed_gull_Iceland_1113_2011 | H4N8 | 10/16/11 | CY149363 | Eurasian |
| A_great_black-backed_gull_Iceland_1393_2011 | H2N5 | 10/25/11 | CY149411 | Eurasian |
| A_great_black-backed_gull_Iceland_1395_2011 | H2N5 | 10/25/11 | CY149427 | Eurasian |
| A_great_black-headed_gull_Qinghai_8_2009 | H5N1 | 2009/06/ | GU477556 | Eurasian |
| A_great_black-headed_gull_Qinghai_8_2009 | H5N1 | 2009/06/ | GU477556 | Eurasian |
| A_greylag_goose_Iceland_0911_2011 | H6N8 | 11/1/11 | CY149475 | Eurasian |
| A_greylag_goose_Iceland_0911_2011 | H6N8 | 11/1/11 | CY149475 | Eurasian |
| A_greylag_goose_Iceland_0921_2011 | H6N8 | 11/1/11 | CY149483 | Eurasian |
| A_greylag_goose_Iceland_0921_2011 | H6N8 | 11/1/11 | CY149483 | Eurasian |
| A_greylag_goose_Iceland_0926_2011 | H6N8 | 11/1/11 | CY149499 | Eurasian |
| A_greylag_goose_Iceland_0926_2011 | H6N8 | 11/1/11 | CY149499 | Eurasian |
| A_greylag_goose_Iceland_0948_2011 | H6N5 | 11/1/11 | CY149507 | Eurasian |
| A_greylag_goose_Iceland_0948_2011 | H6N5 | 11/1/11 | CY149507 | Eurasian |
| A_greylag_goose_Iceland_0953_2011 | H6N5 | 11/1/11 | CY149515 | Eurasian |
| A_greylag_goose_Iceland_0953_2011 | H6N5 | 11/1/11 | CY149515 | Eurasian |
| A_greylag_goose_Iceland_0961_2011 | H6N8 | 11/1/11 | CY149523 | Eurasian |
| A_greylag_goose_Iceland_0961_2011 | H6N8 | 11/1/11 | CY149523 | Eurasian |
| A_greylag_goose_Iceland_0976_2011 | H6N8 | 11/1/11 | CY149531 | Eurasian |
| A_greylag_goose_Iceland_0976_2011 | H6N8 | 11/1/11 | CY149531 | Eurasian |
| A_greylag_goose_Iceland_0980_2011 | H6N8 | 11/1/11 | CY149539 | Eurasian |
| A_greylag_goose_Iceland_0980_2011 | H6N8 | 11/1/11 | CY149539 | Eurasian |
| A_greylag_goose_Iceland_1459_2011 | H6N5 | 11/1/11 | CY149451 | Eurasian |
| A_greylag_goose_Iceland_1474_2011 | H6N8 | 11/1/11 | CY149459 | Eurasian |
| A_greylag_goose_Iceland_1482_2011 | H6N8 | 11/1/11 | CY149467 | Eurasian |
| A_greylag_goose_Iceland_1482_2011 | H6N8 | 11/1/11 | CY149467 | Eurasian |
| A_gull_Moscow_3100_2006 | H6N2 | 2006/10/ | EU152234 | Eurasian |
| A_gull_Moscow_3100_2006 | H6N2 | 2006/10/ | EU152234 | Eurasian |
| A_herring_gull_Iceland_1320_2011 | H2N5 | 10/23/11 | CY149379 | Eurasian |
| A_herring_gull_Iceland_1342_2011 | H2N5 | 10/25/11 | CY149387 | Eurasian |
| A_herring_gull_Iceland_1359_2011 | H2N5 | 10/25/11 | CY149395 | Eurasian |
| A_herring_gull_Iceland_1394_2011 | H2N5 | 10/25/11 | CY149419 | Eurasian |
| A_herring_gull_Iceland_1436_2011 | H2N5 | 10/29/11 | CY149435 | Eurasian |
| A_herring_gull_Mongolia_454_2008 | H13N8 | 2008/09/ | JF775477 | Eurasian |
| A_herring_gull_Mongolia_454_2008 | H13N8 | 2008/09/ | JF775477 | Eurasian |
| A_herring_gull_Netherlands_4_2006 | H10N4 | 2006 | CY077039 | Eurasian |
| A_Iceland_gull_Iceland_1124_2011 | H2N5 | 10/16/11 | CY149371 | Eurasian |
| A_lesser_black-backed_gull_Iceland_145_2010 | H11N2 | 5/23/10 | CY138144 | Eurasian |
| A_mallard_Bavaria_185-26_2008 | H1N1 | 9/22/08 | HQ259229 | Eurasian |
| A_mallard_Bavaria_185-26_2008 | H1N1 | 9/22/08 | HQ259229 | Eurasian |
| A_mallard_Bavaria_185-8_2008 | H1N1 | 9/22/08 | HQ259221 | Eurasian |
| A_mallard_Bavaria_185-8_2008 | H1N1 | 9/22/08 | HQ259221 | Eurasian |
| A_mallard_Iceland_1007_2011 | H3N6 | 10/14/11 | CY149339 | Eurasian |
| A_mallard_Netherlands_02_2000 | H10N7 | 2000 | CY076952 | Eurasian |
| A_mallard_Netherlands_1_2007 | H3N2 | 2007 | CY043823 | Eurasian |
| A_mallard_Netherlands_1_2010 | H2N3 | 2010 | CY122315 | Eurasian |
| A_mallard_Netherlands_11_2007 | H6N5 | 2007 | CY041409 | Eurasian |
| A_mallard_Netherlands_12_2001 | H4N2 | 12/3/01 | CY060217 | Eurasian |
| A_mallard_Netherlands_12_2008 | H2N3 | 2008 | CY122307 | Eurasian |
| A_mallard_Netherlands_13_2001 | H2N9 | 2001 | CY122006 | Eurasian |
| A_mallard_Netherlands_14_2001 | H2N3 | 2001 | CY121998 | Eurasian |
| A_mallard_Netherlands_14_2007 | H2N2 | 2007 | CY121982 | Eurasian |
| A_mallard_Netherlands_2_2005 | H4N2 | 2005 | CY041257 | Eurasian |
| A_mallard_Netherlands_20_2005 | H12N8 | 2005 | CY076975 | Eurasian |
| A_mallard_Netherlands_26_2005 | H11N2 | 2005 | CY041425 | Eurasian |
| A_mallard_Netherlands_29_2010 | H2N3 | 2010 | CY122299 | Eurasian |
| A_mallard_Netherlands_3_2005 | H3N8 | 2005 | CY041249 | Eurasian |
| A_mallard_Netherlands_30_2006 | H1N4 | 2006 | CY076904 | Eurasian |
| A_mallard_Netherlands_31_2006 | H2N2 | 2006 | CY121974 | Eurasian |
| A_mallard_Netherlands_37_2008 | H2N3 | 2008 | CY122275 | Eurasian |
| A_mallard_Netherlands_42_2006 | H11N9 | 2006 | CY077063 | Eurasian |
| A_mallard_Netherlands_5_2001 | H3N6 | 10/5/01 | CY060340 | Eurasian |
| A_mallard_Netherlands_58_2008 | H2N3 | 2008 | CY122283 | Eurasian |
| A_mallard_Netherlands_71_2006 | H6N2 | 2006 | CY041401 | Eurasian |
| A_mallard_PT_35910-2_2006 | H4N6 | 10/30/06 | HM849024 | Eurasian |
| A_mallard_Sweden_103_2005 | H2N9 | 2005 | CY122227 | Eurasian |
| A_mallard_Sweden_107_2005 | H2N3 | 2005 | CY122235 | Eurasian |
| A_mallard_Sweden_109_2002 | H2N7 | 2002 | CY122038 | Eurasian |
| A_mallard_Sweden_111_2002 | H2N7 | 2002 | CY122046 | Eurasian |
| A_mallard_Sweden_112_2002 | H2N7 | 2002 | CY122058 | Eurasian |
| A_mallard_Sweden_113_2002 | H2N1 | 2002 | CY122066 | Eurasian |
| A_mallard_Sweden_12_2003 | H2N3 | 2003 | CY122122 | Eurasian |
| A_mallard_Sweden_24_2002 | H8N4 | 11/7/02 | CY060246 | Eurasian |
| A_mallard_Sweden_30_2002 | H2N5 | 2002 | CY122074 | Eurasian |
| A_mallard_Sweden_30_2005 | H6N1 | 2005 | CY043831 | Eurasian |
| A_mallard_Sweden_32_2002 | H2N3 | 2002 | CY122082 | Eurasian |
| A_mallard_Sweden_33_2002 | H2N3 | 2002 | CY122090 | Eurasian |
| A_mallard_Sweden_43_2005 | H2N9 | 2005 | CY122162 | Eurasian |
| A_mallard_Sweden_45_2002 | H11N8 | 11/13/02 | CY060278 | Eurasian |
| A_mallard_Sweden_45_2005 | H2N3 | 2005 | CY041337 | Eurasian |
| A_mallard_Sweden_46_2002 | H4N3 | 11/14/02 | CY060285 | Eurasian |
| A_mallard_Sweden_48_2002 | H11N9 | 11/14/02 | CY060291 | Eurasian |
| A_mallard_Sweden_49_2005 | H2N9 | 2005 | CY122170 | Eurasian |
| A_mallard_Sweden_5_2005 | H2N3 | 2005 | CY122243 | Eurasian |
| A_mallard_Sweden_51_2005 | H2N3 | 2005 | CY122178 | Eurasian |
| A_mallard_Sweden_53_2003 | H2N3 | 2003 | CY122154 | Eurasian |
| A_mallard_Sweden_54_2003 | H6N1 | 11/30/03 | KF695220 | Eurasian |
| A_mallard_Sweden_54_2003 | H6N1 | 11/30/03 | KF695220 | Eurasian |
| A_mallard_Sweden_55_2002 | H11N2 | 11/16/02 | CY060321 | Eurasian |
| A_mallard_Sweden_57_2002 | H2N3 | 2002 | CY122099 | Eurasian |
| A_mallard_Sweden_58_2005 | mixed | 2005 | CY121914 | Eurasian |
| A_mallard_Sweden_58_2005 | mixed | 2005 | CY121915 | Eurasian |
| A_mallard_Sweden_58112_2006 | H2N2 | 11/2/06 | CY121934 | Eurasian |
| A_mallard_Sweden_58451_2006 | H2N2 | 11/5/06 | CY121942 | Eurasian |
| A_mallard_Sweden_6_2002 | H2N3 | 2002 | CY122014 | Eurasian |
| A_mallard_Sweden_6_2002 | H2N3 | 10/25/02 | CY060363 | Eurasian |
| A_mallard_Sweden_60_2005 | mixed | 2005 | CY122187 | Eurasian |
| A_mallard_Sweden_65_2002 | H10N9 | 11/17/02 | CY060355 | Eurasian |
| A_mallard_Sweden_65_2005 | H4N3 | 2005 | CY041361 | Eurasian |
| A_mallard_Sweden_68597_2007 | H2N2 | 10/19/07 | CY121958 | Eurasian |
| A_mallard_Sweden_68735_2007 | H2N2 | 10/22/07 | CY121950 | Eurasian |
| A_mallard_Sweden_7_2003 | H10N8 | 5/24/03 | CY060371 | Eurasian |
| A_mallard_Sweden_70_2002 | H2N3 | 2002 | CY122106 | Eurasian |
| A_mallard_Sweden_72_2005 | H2N9 | 2005 | CY122195 | Eurasian |
| A_mallard_Sweden_73_2005 | H2N3 | 2005 | CY122203 | Eurasian |
| A_mallard_Sweden_81_2003 | H2N3 | 2003 | CY122130 | Eurasian |
| A_mallard_Sweden_84_2003 | H2N3 | 2003 | CY122138 | Eurasian |
| A_mallard_Sweden_88_2002 | H2N1 | 2002 | CY122022 | Eurasian |
| A_mallard_Sweden_88_2005 | mixed | 2005 | CY121926 | Eurasian |
| A_mallard_Sweden_9_2003 | H2N7 | 2003 | CY122146 | Eurasian |
| A_mallard_Sweden_91_2005 | H2N9 | 2005 | CY122211 | Eurasian |
| A_mallard_Sweden_93_2005 | H2N3 | 2005 | CY122219 | Eurasian |
| A_mallard_Sweden_99_2002 | H2N3 | 2002 | CY122030 | Eurasian |
| A_Mongolian_gull_Mongolia_401_2007 | H13N6 | 8/28/07 | GQ907317 | Eurasian |
| A_Mongolian_gull_Mongolia_401_2007 | H13N6 | 8/28/07 | GQ907317 | Eurasian |
| A_Mongolian_gull_Mongolia_405_2007 | H13N6 | 8/28/07 | GQ907325 | Eurasian |
| A_Mongolian_gull_Mongolia_405_2007 | H13N6 | 8/28/07 | GQ907325 | Eurasian |
| A_Muscovy_duck_France_09010_2009 | H6N1 | 2009/03/ | JN860169 | Eurasian |
| A_Muscovy_duck_France_09010_2009 | H6N1 | 2009/03/ | JN860169 | Eurasian |
| A_mute_swan_Netherlands_1_2006 | H10N7 | 2006 | CY077047 | Eurasian |
| A_northern_pintail_Sweden_1_2003 | H2N3 | 2003 | CY122114 | Eurasian |
| A_northern_pintail_Sweden_1_2003 | H2N3 | 9/17/03 | CY060421 | Eurasian |
| A_northern_shoveler_Netherlands_1_2006 | H8N4 | 2006 | CY077031 | Eurasian |
| A_pink-footed_goose_Iceland_0987_2011 | H6N8 | 11/1/11 | CY149547 | Eurasian |
| A_pink-footed_goose_Iceland_0987_2011 | H6N8 | 11/1/11 | CY149547 | Eurasian |
| A_turnstone_Netherlands_1_2007 | H3N8 | 2007 | CY041353 | Eurasian |
| A_American_black_duck_Illinois_08OS2688_2008 | H5N2 | 10/26/08 | CY079459 | North American |
| A_American_black_duck_Illinois_4119_2009 | H8N4 | 11/29/09 | CY097541 | North American |
| A_American_black_duck_New_Brunswick_00321_2010 | H1N1 | 8/8/10 | CY138647 | North American |
| A_American_black_duck_New_Brunswick_00322_2010 | H3N8 | 8/8/10 | CY138655 | North American |
| A_American_black_duck_New_Brunswick_00326_2010 | H1N1 | 8/8/10 | CY138671 | North American |
| A_American_black_duck_New_Brunswick_00328_2010 | H1N1 | 8/8/10 | CY138679 | North American |
| A_American_black_duck_New_Brunswick_00344_2010 | H7N7 | 8/8/10 | CY138776 | North American |
| A_American_black_duck_New_Brunswick_00385_2010 | H1N1 | 8/9/10 | CY138824 | North American |
| A_American_black_duck_New_Brunswick_00410_2010 | H1N1 | 8/10/10 | CY138849 | North American |
| A_American_black_duck_New_Brunswick_00424_2010 | H3N8 | 8/10/10 | CY138857 | North American |
| A_American_black_duck_New_Brunswick_00425_2010 | H3N8 | 8/10/10 | CY138865 | North American |
| A_American_black_duck_New_Brunswick_00454_2010 | H3N8 | 8/11/10 | CY138873 | North American |
| A_American_black_duck_New_Brunswick_00464_2010 | H4N6 | 8/12/10 | CY138052 | North American |
| A_American_black_duck_New_Brunswick_00467_2010 | H3N8 | 8/12/10 | CY138881 | North American |
| A_American_black_duck_New_Brunswick_00468_2010 | H4N6 | 8/12/10 | CY138889 | North American |
| A_American_black_duck_New_Brunswick_00469_2010 | H4N6 | 8/12/10 | CY138897 | North American |
| A_American_black_duck_New_Brunswick_00470_2010 | H4N6 | 8/12/10 | CY138905 | North American |
| A_American_black_duck_New_Brunswick_00471_2010 | H10N6 | 8/12/10 | CY138913 | North American |
| A_American_black_duck_New_Brunswick_00472_2010 | H4N6 | 8/12/10 | CY138921 | North American |
| A_American_black_duck_New_Brunswick_00473_2010 | H4N6 | 8/12/10 | CY138060 | North American |
| A_American_black_duck_New_Brunswick_00477_2010 | H10N6 | 8/12/10 | CY138940 | North American |
| A_American_black_duck_New_Brunswick_00478_2010 | H4N6 | 8/12/10 | CY138948 | North American |
| A_American_black_duck_New_Brunswick_00481_2010 | H4N6 | 8/12/10 | CY138956 | North American |
| A_American_black_duck_New_Brunswick_00484_2010 | H3N8 | 8/12/10 | CY138964 | North American |
| A_American_black_duck_New_Brunswick_00485_2010 | H3N8 | 8/12/10 | CY138972 | North American |
| A_American_black_duck_New_Brunswick_00487_2010 | H4N8 | 8/12/10 | CY138991 | North American |
| A_American_black_duck_New_Brunswick_00499_2010 | H4N6 | 8/12/10 | CY139039 | North American |
| A_American_black_duck_New_Brunswick_00500_2010 | H3N8 | 8/12/10 | CY139047 | North American |
| A_American_black_duck_New_Brunswick_00520_2010 | H4N6 | 8/12/10 | CY139066 | North American |
| A_American_black_duck_New_Brunswick_00522_2010 | H3N8 | 8/13/10 | CY139074 | North American |
| A_American_black_duck_New_Brunswick_00523_2010 | H4N6 | 8/13/10 | CY139082 | North American |
| A_American_black_duck_New_Brunswick_00525_2010 | H3N8 | 8/13/10 | CY138072 | North American |
| A_American_black_duck_New_Brunswick_00558_2010 | H3N8 | 8/14/10 | CY139090 | North American |
| A_American_black_duck_New_Brunswick_00587_2010 | H4N6 | 8/15/10 | CY139098 | North American |
| A_American_black_duck_New_Brunswick_00608_2010 | H3N8 | 8/16/10 | CY139114 | North American |
| A_American_black_duck_New_Brunswick_00612_2010 | H10N7 | 8/16/10 | CY139131 | North American |
| A_American_black_duck_New_Brunswick_00614_2010 | H3N8 | 8/16/10 | CY139139 | North American |
| A_American_black_duck_New_Brunswick_00615_2010 | H4N8 | 8/16/10 | CY139147 | North American |
| A_American_black_duck_New_Brunswick_00616_2010 | H3N8 | 8/16/10 | CY139155 | North American |
| A_American_black_duck_New_Brunswick_00618_2010 | H3N6 | 8/16/10 | CY139172 | North American |
| A_American_black_duck_New_Brunswick_00619_2010 | H3N6 | 8/16/10 | CY139180 | North American |
| A_American_black_duck_New_Brunswick_00620_2010 | H3N6 | 8/16/10 | CY139188 | North American |
| A_American_black_duck_New_Brunswick_00622_2010 | H3N6 | 8/16/10 | CY139206 | North American |
| A_American_black_duck_New_Brunswick_00623_2010 | H4N8 | 8/16/10 | CY139214 | North American |
| A_American_black_duck_New_Brunswick_00624_2010 | H3N6 | 8/16/10 | CY139222 | North American |
| A_American_black_duck_New_Brunswick_00687_2010 | H3N8 | 8/9/10 | CY139246 | North American |
| A_American_black_duck_New_Brunswick_00867_2010 | H5N2 | 9/13/10 | CY139334 | North American |
| A_American_black_duck_New_Brunswick_00878_2010 | H4N9 | 9/12/10 | CY139351 | North American |
| A_American_black_duck_New_Brunswick_00886_2010 | H4N9 | 9/13/10 | CY139376 | North American |
| A_American_black_duck_New_Brunswick_00906_2010 | H10N7 | 9/14/10 | CY139392 | North American |
| A_American_black_duck_New_Brunswick_00909_2010 | H10N7 | 9/14/10 | CY139400 | North American |
| A_American_black_duck_New_Brunswick_00924_2010 | H10N7 | 9/15/10 | CY139428 | North American |
| A_American_black_duck_New_Brunswick_00949_2010 | H4N6 | 9/15/10 | CY138080 | North American |
| A_American_black_duck_New_Brunswick_00953_2010 | H3N8 | 9/15/10 | CY139466 | North American |
| A_American_black_duck_New_Brunswick_00955_2010 | H4N8 | 9/15/10 | CY138088 | North American |
| A_American_black_duck_New_Brunswick_00971_2010 | H10N6 | 9/16/10 | CY139496 | North American |
| A_American_black_duck_New_Brunswick_00986_2010 | H4N6 | 9/16/10 | CY139512 | North American |
| A_American_black_duck_New_Brunswick_00991_2010 | H4N6 | 9/16/10 | CY139538 | North American |
| A_American_black_duck_New_Brunswick_00998_2010 | H12N6 | 9/17/10 | CY139557 | North American |
| A_American_black_duck_New_Brunswick_01989_2007 | H1N1 | 8/6/07 | CY129381 | North American |
| A_American_black_duck_New_Brunswick_02375_2007 | H4N8 | 8/5/07 | CY128765 | North American |
| A_American_black_duck_New_Brunswick_02396_2007 | H4N6 | 8/11/07 | CY128773 | North American |
| A_American_black_duck_New_Brunswick_02485_2007 | H11N9 | 9/17/07 | CY129268 | North American |
| A_American_black_duck_New_Brunswick_02490_2007 | H7N3 | 9/17/07 | CY129341 | North American |
| A_American_black_duck_New_Brunswick_02491_2007 | H3N8 | 9/17/07 | CY129157 | North American |
| A_American_black_duck_New_Brunswick_02493_2007 | H7N3 | 9/17/07 | CY128721 | North American |
| A_American_black_duck_New_Brunswick_02496_2007 | H3N8 | 9/18/07 | CY129317 | North American |
| A_American_black_duck_New_Brunswick_02497_2007 | H3N8 | 9/18/07 | CY129260 | North American |
| A_American_black_duck_New_Brunswick_02502_2007 | H3N8 | 9/18/07 | CY129301 | North American |
| A_American_black_duck_New_Brunswick_02507_2007 | H3N8 | 9/18/07 | CY129212 | North American |
| A_American_black_duck_New_Brunswick_02519_2007 | H11N9 | 9/19/07 | CY129077 | North American |
| A_American_black_duck_New_Brunswick_02525_2007 | H3N8 | 9/24/07 | CY129365 | North American |
| A_American_black_duck_New_Brunswick_02527_2007 | H4N6 | 9/24/07 | CY129325 | North American |
| A_American_black_duck_New_Brunswick_02528_2007 | H4N6 | 9/24/07 | CY129252 | North American |
| A_American_black_duck_New_Brunswick_02531_2007 | H4N6 | 9/25/07 | CY128757 | North American |
| A_American_black_duck_New_Brunswick_02549_2007 | H3N8 | 9/26/07 | CY128675 | North American |
| A_American_black_duck_New_Brunswick_02629_2007 | H3N8 | 9/22/07 | CY129053 | North American |
| A_American_black_duck_New_Brunswick_02646_2007 | H4N6 | 9/23/07 | CY128781 | North American |
| A_American_black_duck_New_Brunswick_02649_2007 | H3N6 | 9/24/07 | CY129101 | North American |
| A_American_black_duck_New_Brunswick_02650_2007 | H3N2 | 9/24/07 | CY129069 | North American |
| A_American_black_duck_New_Brunswick_02651_2007 | H3N2 | 9/24/07 | CY129220 | North American |
| A_American_black_duck_New_Brunswick_02653_2007 | H4N6 | 9/24/07 | CY129228 | North American |
| A_American_black_duck_New_Brunswick_02656_2007 | H3N8 | 9/24/07 | CY129194 | North American |
| A_American_black_duck_New_Brunswick_02659_2007 | H4N6 | 9/24/07 | CY129085 | North American |
| A_American_black_duck_New_Brunswick_02719_2007 | H4N6 | 9/19/07 | CY129117 | North American |
| A_American_black_duck_New_Brunswick_02722_2007 | H4N6 | 9/19/07 | CY129109 | North American |
| A_American_black_duck_New_Brunswick_02725_2007 | H3N8 | 9/19/07 | CY129061 | North American |
| A_American_black_duck_New_Brunswick_02726_2007 | H4N6 | 9/19/07 | CY129133 | North American |
| A_American_black_duck_New_Brunswick_02727_2007 | H4N6 | 9/19/07 | CY129141 | North American |
| A_American_black_duck_New_Brunswick_02728_2007 | H4N6 | 9/19/07 | CY129093 | North American |
| A_American_black_duck_New_Brunswick_02729_2007 | H4N6 | 9/19/07 | CY129149 | North American |
| A_American_black_duck_New_Brunswick_02730_2007 | H4N6 | 9/19/07 | CY129165 | North American |
| A_American_black_duck_New_Brunswick_02749_2007 | H3N8 | 9/4/07 | CY129349 | North American |
| A_American_black_duck_New_Brunswick_03395_2009 | H3N8 | 8/9/09 | CY129021 | North American |
| A_American_black_duck_New_Brunswick_03398_2009 | H3N8 | 8/9/09 | CY128989 | North American |
| A_American_black_duck_New_Brunswick_03408_2009 | H3N8 | 8/10/09 | CY129013 | North American |
| A_American_black_duck_New_Brunswick_03451_2009 | H3N9 | 8/12/09 | CY128957 | North American |
| A_American_black_duck_New_Brunswick_03495_2009 | H3N8 | 9/9/09 | CY129037 | North American |
| A_American_black_duck_New_Brunswick_03509_2009 | H3N8 | 9/10/09 | CY129029 | North American |
| A_American_black_duck_New_Brunswick_03511_2009 | H4N6 | 9/10/09 | CY128981 | North American |
| A_American_black_duck_New_Brunswick_03530_2009 | H4N6 | 9/10/09 | CY128491 | North American |
| A_American_black_duck_New_Brunswick_03531_2009 | H4N6 | 9/10/09 | CY128523 | North American |
| A_American_black_duck_New_Brunswick_03532_2009 | H4N6 | 9/10/09 | CY128531 | North American |
| A_American_black_duck_New_Brunswick_03534_2009 | H4N6 | 9/10/09 | CY128515 | North American |
| A_American_black_duck_New_Brunswick_03551_2009 | H4N6 | 9/11/09 | CY128507 | North American |
| A_American_black_duck_New_Brunswick_03552_2009 | H4N6 | 9/11/09 | CY128997 | North American |
| A_American_black_duck_New_Brunswick_03553_2009 | H4N6 | 9/11/09 | CY128973 | North American |
| A_American_black_duck_New_Brunswick_03554_2009 | H4N6 | 9/11/09 | CY128555 | North American |
| A_American_black_duck_New_Brunswick_03556_2009 | H4N6 | 9/11/09 | CY128539 | North American |
| A_American_black_duck_New_Brunswick_03559_2009 | H4N6 | 9/11/09 | CY128547 | North American |
| A_American_black_duck_New_Brunswick_04388_2007 | H7N3 | 8/16/07 | CY129333 | North American |
| A_American_black_duck_New_Brunswick_04395_2007 | H10N7 | 9/4/07 | CY129244 | North American |
| A_American_black_duck_New_Brunswick_04399_2007 | H10N7 | 9/4/07 | CY128729 | North American |
| A_American_black_duck_New_Brunswick_04484_2007 | H3N8 | 9/4/07 | CY129285 | North American |
| A_American_black_duck_New_Brunswick_19347_2006 | H4N6 | 9/14/06 | CY045310 | North American |
| A_American_black_duck_New_Brunswick_19497_2006 | H4N6 | 9/18/06 | CY095449 | North American |
| A_American_black_duck_New_Brunswick_19502_2006 | H4N6 | 9/18/06 | CY045318 | North American |
| A_American_black_duck_New_Brunswick_25182_2007 | H3N6 | 9/21/07 | CY047703 | North American |
| A_American_black_duck_Nova_Scotia_00083_2010 | H1N1 | 9/10/10 | CY138535 | North American |
| A_American_black_duck_Nova_Scotia_00086_2010 | H1N1 | 9/10/10 | CY138559 | North American |
| A_American_black_duck_Nova_Scotia_00089_2010 | H1N1 | 9/10/10 | CY138575 | North American |
| A_American_black_duck_Nova_Scotia_00090_2010 | H1N1 | 9/10/10 | CY138583 | North American |
| A_American_black_duck_Nova_Scotia_00091_2010 | H1N1 | 9/10/10 | CY138591 | North American |
| A_American_black_duck_Nova_Scotia_00092_2010 | H1N1 | 9/10/10 | CY138599 | North American |
| A_American_black_duck_Nova_Scotia_00094_2010 | H1N1 | 9/10/10 | CY138615 | North American |
| A_American_black_duck_Nova_Scotia_00096_2010 | H1N1 | 9/10/10 | CY138623 | North American |
| A_American_black_duck_Nova_Scotia_00098_2010 | H1N1 | 9/10/10 | CY138631 | North American |
| A_American_black_duck_Nova_Scotia_00099_2010 | H1N1 | 9/10/10 | CY138639 | North American |
| A_American_black_duck_Nova_Scotia_02043_2007 | H8N4 | 8/8/07 | CY128901 | North American |
| A_American_black_duck_Nova_Scotia_02317_2007 | H4N6 | 8/25/07 | CY125661 | North American |
| A_American_black_duck_Nova_Scotia_02319_2007 | H4N6 | 8/25/07 | CY128859 | North American |
| A_American_black_duck_Nova_Scotia_03273_2009 | H3N8 | 8/6/09 | CY125629 | North American |
| A_American_black_duck_Prince_Edward_Island_00672_2010 | H3N8 | 8/4/10 | CY139230 | North American |
| A_American_black_duck_Prince_Edward_Island_00673_2010 | H3N8 | 8/4/10 | CY139238 | North American |
| A_American_black_duck_Prince_Edward_Island_00788_2010 | H3N8 | 8/25/10 | CY139286 | North American |
| A_American_black_duck_Prince_Edward_Island_02661_2007 | H4N6 | 8/16/07 | CY129173 | North American |
| A_American_black_duck_Prince_Edward_Island_02662_2007 | H4N6 | 8/16/07 | CY125645 | North American |
| A_American_black_duck_Prince_Edward_Island_02683_2007 | H4N6 | 8/20/07 | CY128909 | North American |
| A_American_black_duck_Prince_Edward_Island_02684_2007 | H4N6 | 8/20/07 | CY128941 | North American |
| A_American_black_duck_Prince_Edward_Island_02685_2007 | H4N6 | 8/20/07 | CY128835 | North American |
| A_American_black_duck_Prince_Edward_Island_02697_2007 | H4N6 | 8/21/07 | CY128843 | North American |
| A_American_black_duck_Prince_Edward_Island_02700_2007 | H4N6 | 8/22/07 | CY128851 | North American |
| A_American_black_duck_Prince_Edward_Island_02708_2007 | H4N6 | 8/23/07 | CY125637 | North American |
| A_American_black_duck_Wisconsin_10OS3949_2010 | H7N8 | 11/18/10 | CY133281 | North American |
| A_American_black_duck_Wisconsin_2542_2009 | H4N2 | 10/5/09 | CY096985 | North American |
| A_American_coot_California_20181-006 | 2007_H10N3 | 3/2/07 | CY076100 | North American |
| A_American_coot_Illinois_3405_2009 | H10N3 | 10/31/09 | CY097166 | North American |
| A_American_coot_Mississippi_09OS615_2009 | H10N3 | 1/24/09 | CY079411 | North American |
| A_American_coot_Oregon_20589-007_2007 | H3N8 | 12/17/07 | CY076172 | North American |
| A_American_green-winged_teal_California_27790_2007 | H11N9 | 1/24/07 | CY076356 | North American |
| A_American_green-winged_teal_California_28228_2007 | H7N6 | 1/27/07 | CY076012 | North American |
| A_American_green-winged_teal_California_28855_2007 | H7N3 | 2/3/07 | CY047018 | North American |
| A_American_green-winged_teal_California_44242-906_2007 | H7N3 | 1/3/07 | CY076236 | North American |
| A_American_green-winged_teal_California_44287-066_2007 | H11N9 | 1/24/07 | CY076348 | North American |
| A_American_green-winged_teal_California_44287-084_2007 | H7N3 | 1/24/07 | CY076364 | North American |
| A_American_green-winged_teal_California_44287-305_2007 | H7N6 | 1/27/07 | CY076420 | North American |
| A_American_green-winged_teal_California_44287-713_2007 | H7N3 | 2/3/07 | CY045382 | North American |
| A_American_green-winged_teal_California_44363-067_2007 | H11N9 | 11/3/07 | CY076692 | North American |
| A_American_green-winged_teal_California_HKWF609_2007 | H5N2 | 11/10/07 | CY033441 | North American |
| A_American_green-winged_teal_Illinois_08OS2713_2008 | H10N7 | 10/26/08 | CY079475 | North American |
| A_American_green-winged_teal_Illinois_10OS1551_2010 | H4N6 | 9/4/10 | CY132900 | North American |
| A_American_green-winged_teal_Illinois_10OS1598_2010 | H4N8 | 9/5/10 | CY132924 | North American |
| A_American_green-winged_teal_Illinois_10OS3329_2010 | H7N7 | 10/30/10 | CY132980 | North American |
| A_American_green-winged_teal_Illinois_10OS3343_2010 | H2N3 | 10/30/10 | CY132996 | North American |
| A_American_green-winged_teal_Illinois_10OS3368_2010 | H7N7 | 10/30/10 | CY133004 | North American |
| A_American_green-winged_teal_Illinois_10OS3589_2010 | H6N2 | 10/30/10 | CY133020 | North American |
| A_American_green-winged_teal_Illinois_10OS3662_2010 | H11N2 | 10/31/10 | CY133536 | North American |
| A_American_green-winged_teal_Illinois_10OS4014_2010 | H7N3 | 11/27/10 | CY133044 | North American |
| A_American_green-winged_teal_Illinois_2479_2009 | H2N3 | 10/17/09 | CY097210 | North American |
| A_American_green-winged_teal_Illinois_3054_2009 | H1N2 | 10/17/09 | CY097202 | North American |
| A_American_green-winged_teal_Interior_Alaska_10BM04207R0_2010 | H3N6 | 7/13/10 | CY130476 | North American |
| A_American_green-winged_teal_Interior_Alaska_10BM05165R0_2010 | H4N6 | 7/22/10 | CY135678 | North American |
| A_American_green-winged_teal_Interior_Alaska_10BM05376R0_2010 | H3N8 | 7/25/10 | CY135736 | North American |
| A_American_green-winged_teal_Interior_Alaska_10BM06728R0_2010 | H4N6 | 7/30/10 | CY136050 | North American |
| A_American_green-winged_teal_Interior_Alaska_10BM07000R0_2010 | H4N6 | 7/31/10 | CY136026 | North American |
| A_American_green-winged_teal_Interior_Alaska_10BM07573R0_2010 | H3N8 | 8/2/10 | CY143491 | North American |
| A_American_green-winged_teal_Interior_Alaska_10BM07649R0_2010 | H3N8 | 8/2/10 | CY143507 | North American |
| A_American_green-winged_teal_Interior_Alaska_10BM07863R0_2010 | H4N6 | 8/3/10 | CY143523 | North American |
| A_American_green-winged_teal_Interior_Alaska_10BM07917R0_2010 | H3N8 | 8/3/10 | CY143531 | North American |
| A_American_green-winged_teal_Interior_Alaska_10BM08134R0_2010 | H3N8 | 8/4/10 | CY143555 | North American |
| A_American_green-winged_teal_Interior_Alaska_10BM08222R0_2010 | H3N8 | 8/4/10 | CY143563 | North American |
| A_American_green-winged_teal_Interior_Alaska_10BM08226R0_2010 | H4N6 | 8/4/10 | CY143571 | North American |
| A_American_green-winged_teal_Interior_Alaska_10BM08228R0_2010 | H3N8 | 8/4/10 | CY143579 | North American |
| A_American_green-winged_teal_Interior_Alaska_10BM08422R0_2010 | H3N8 | 8/4/10 | CY143587 | North American |
| A_American_green-winged_teal_Interior_Alaska_3_2007 | H3N8 | 10/17/07 | CY038383 | North American |
| A_American_green-winged_teal_Interior_Alaska_4_2007 | H3N8 | 9/7/07 | CY039799 | North American |
| A_American_green-winged_teal_Interior_Alaska_7MP1049_2007 | H3N8 | 9/10/07 | CY077387 | North American |
| A_American_green-winged_teal_Interior_Alaska_7MP1651_2007 | H3N8 | 9/2/07 | CY077245 | North American |
| A_American_green-winged_teal_Interior_Alaska_7MP2225_2007 | H3N8 | 9/16/07 | CY047034 | North American |
| A_American_green-winged_teal_Interior_Alaska_9BM12318R0_2009 | H3N8 | 9/2/09 | CY143177 | North American |
| A_American_green-winged_teal_Interior_Alaska_9BM12356R0_2009 | H3N8 | 9/2/09 | CY143201 | North American |
| A_American_green-winged_teal_Interior_Alaska_9BM12714R1_2009 | H3N8 | 9/6/09 | CY143451 | North American |
| A_American_green-winged_teal_Interior_Alaska_9BM3595R1_2009 | H10N7 | 7/23/09 | CY135462 | North American |
| A_American_green-winged_teal_Interior_Alaska_9BM4407R0_2009 | H10N7 | 7/27/09 | CY135406 | North American |
| A_American_green-winged_teal_Interior_Alaska_9BM5041R0_2009 | H4N6 | 7/29/09 | CY135534 | North American |
| A_American_green-winged_teal_Interior_Alaska_9BM5045R0_2009 | H8N4 | 7/29/09 | CY135542 | North American |
| A_American_green-winged_teal_Interior_Alaska_9BM5277R0_2009 | H4N6 | 7/30/09 | CY135558 | North American |
| A_American_green-winged_teal_Interior_Alaska_9BM6258R0_2009 | H4N6 | 8/3/09 | CY142617 | North American |
| A_American_green-winged_teal_Interior_Alaska_9BM6410R0_2009 | H3N8 | 8/3/09 | CY142633 | North American |
| A_American_green-winged_teal_Interior_Alaska_9BM8129R0_2009 | H4N6 | 8/9/09 | CY142977 | North American |
| A_American_green-winged_teal_Manitoba_23884_2007 | H4N6 | 8/21/07 | CY047687 | North American |
| A_American_green-winged_teal_Minnesota_AI09-3589_2009 | H6N1 | 9/12/09 | CY140859 | North American |
| A_American_green-winged_teal_Mississippi_09OS046_2009 | H7N7 | 1/9/09 | CY079315 | North American |
| A_American_green-winged_teal_Mississippi_285_2010 | H3N8 | 1/14/10 | CY097685 | North American |
| A_American_green-winged_teal_Mississippi_300_2010 | H11N9 | 1/16/10 | CY097693 | North American |
| A_American_green-winged_teal_Missouri_10OS4622_2010 | H12N4 | 12/12/10 | CY133364 | North American |
| A_American_green-winged_teal_Oregon_44336-183_2007 | H3N8 | 9/10/07 | CY076484 | North American |
| A_American_green-winged_teal_Texas_AI09-4396_2009 | H3N9 | 9/19/09 | CY140960 | North American |
| A_American_green-winged_teal_Texas_AI09-6046_2009 | H3N8 | 12/13/09 | CY141064 | North American |
| A_American_green-winged_teal_Wisconsin_08OS2270_2008 | H3N8 | 10/19/08 | CY079427 | North American |
| A_American_green-winged_teal_Wisconsin_08OS2291_2008 | H3N2 | 10/20/08 | CY079435 | North American |
| A_American_green-winged_teal_Wisconsin_08OS2292_2008 | H3N2 | 10/20/08 | CY079347 | North American |
| A_American_green-winged_teal_Wisconsin_10OS2767_2010 | H6N8 | 9/26/10 | CY132948 | North American |
| A_American_green-winged_teal_Wisconsin_10OS2847_2010 | H1N1 | 10/2/10 | CY133092 | North American |
| A_American_green-winged_teal_Wisconsin_10OS2955_2010 | H5N2 | 10/3/10 | CY133241 | North American |
| A_American_green-winged_teal_Wisconsin_10OS3127_2010 | H5N2 | 10/20/10 | CY133160 | North American |
| A_American_green-winged_teal_Wisconsin_2530_2009 | H6N2 | 10/17/09 | CY097461 | North American |
| A_American_green-winged_teal_Wisconsin_2690_2009 | H3N8 | 10/20/09 | CY097365 | North American |
| A_American_green-winged_teal_Wisconsin_2743_2009 | H1N1 | 10/17/09 | CY097509 | North American |
| A_American_widgeon_Alaska_7MP1061_2007 | H3N8 | 9/10/07 | CY043983 | North American |
| A_American_widgeon_Interior_Alaska_1_2007 | H3N8 | 9/6/07 | CY035804 | North American |
| A_American_widgeon_Interior_Alaska_7MP1707_2007 | H3N8 | 9/3/07 | CY077363 | North American |
| A_American_wigeon_California_2423_2010 | H6N1 | 10/23/10 | CY133884 | North American |
| A_American_wigeon_California_2997_2010 | H6N1 | 11/17/10 | CY133892 | North American |
| A_American_wigeon_California_3180_2010 | H5N2 | 11/27/10 | CY120634 | North American |
| A_American_wigeon_California_6588_2008 | H6N1 | 11/8/08 | CY093797 | North American |
| A_American_wigeon_California_6610_2008 | H12N5 | 11/8/08 | CY093805 | North American |
| A_American_wigeon_California_8121_2008 | H6N1 | 10/19/08 | CY093813 | North American |
| A_American_wigeon_California_8352_2008 | H12N5 | 10/19/08 | CY094388 | North American |
| A_American_wigeon_California_8363_2008 | H6N1 | 10/19/08 | CY094396 | North American |
| A_American_wigeon_California_8529_2008 | H6N1 | 10/25/08 | CY093829 | North American |
| A_American_wigeon_California_8547_2008 | H6N1 | 10/25/08 | CY093837 | North American |
| A_American_wigeon_California_8658_2008 | H6N1 | 10/29/08 | CY094404 | North American |
| A_American_wigeon_California_8670_2008 | H6N1 | 10/29/08 | CY093845 | North American |
| A_American_wigeon_California_8763_2008 | H6N1 | 11/1/08 | CY093853 | North American |
| A_American_wigeon_California_8910_2008 | H6N1 | 11/8/08 | CY094412 | North American |
| A_American_wigeon_California_9044_2008 | H6N1 | 11/15/08 | CY094420 | North American |
| A_American_wigeon_California_HKWF041C_2007 | H6N1 | 10/21/07 | CY094428 | North American |
| A_American_wigeon_California_HKWF1174_2007 | H6N1 | 12/5/07 | CY033353 | North American |
| A_American_wigeon_California_HKWF295_2007 | H6N5 | 10/31/07 | CY032897 | North American |
| A_American_wigeon_California_HKWF296C_2007 | H6N1 | 10/31/07 | CY093861 | North American |
| A_American_wigeon_California_HKWF353_2007 | H6N1 | 11/3/07 | CY033393 | North American |
| A_American_wigeon_California_HKWF371_2007 | H6N5 | 10/7/07 | CY032701 | North American |
| A_American_wigeon_California_HKWF42_2007 | H6N1 | 10/21/07 | CY033417 | North American |
| A_American_wigeon_California_HKWF450_2007 | H4N7 | 11/4/07 | CY032905 | North American |
| A_American_wigeon_California_HKWF541C_2007 | H6N5 | 11/4/07 | CY094436 | North American |
| A_American_wigeon_Interior_Alaska_7MP1726_2007 | H3N8 | 9/3/07 | CY078922 | North American |
| A_American_wigeon_Interior_Alaska_9BM2501R1_2009 | H3N8 | 7/11/09 | CY130339 | North American |
| A_American_wigeon_Iowa_10OS2748_2010 | H2N2 | 10/17/10 | CY133463 | North American |
| A_American_wigeon_Louisiana_Sg-01031_2008 | H4N6 | 11/8/08 | CY140542 | North American |
| A_American_wigeon_Louisiana_Sg-01032_2008 | H6N2 | 11/9/08 | CY140550 | North American |
| A_American_wigeon_Minnesota_Sg-01067_2008 | H3N8 | 10/12/08 | CY140630 | North American |
| A_American_wigeon_Missouri_10OS4752_2010 | H6N1 | 12/12/10 | CY133431 | North American |
| A_American_wigeon_New_Brunswick_04487_2007 | H3N8 | 9/4/07 | CY125452 | North American |
| A_American_wigeon_New_Brunswick_04488_2007 | H3N8 | 9/4/07 | CY125540 | North American |
| A_American_wigeon_New_Brunswick_04489_2007 | H3N8 | 9/4/07 | CY125516 | North American |
| A_American_wigeon_New_Brunswick_04490_2007 | H3N8 | 9/4/07 | CY125468 | North American |
| A_American_wigeon_New_Brunswick_04491_2007 | H3N8 | 9/4/07 | CY125548 | North American |
| A_American_wigeon_New_Brunswick_04492_2007 | H3N8 | 9/4/07 | CY125556 | North American |
| A_American_wigeon_New_Brunswick_04493_2007 | H3N8 | 9/4/07 | CY125460 | North American |
| A_American_wigeon_New_Brunswick_04494_2007 | H3N8 | 9/4/07 | CY125300 | North American |
| A_American_wigeon_New_Brunswick_04497_2007 | H3N8 | 9/4/07 | CY125532 | North American |
| A_American_wigeon_New_Brunswick_04500_2007 | H3N8 | 9/4/07 | CY125476 | North American |
| A_avian_Delaware_Bay_226_2006 | H7N3 | 5/22/06 | CY039326 | North American |
| A_avian_Southcentral_Alaska_7KW0388R1_2007 | H4N8 | 5/10/07 | CY079692 | North American |
| A_black_scoter_New_Brunswick_00002_2009 | H3N8 | 5/2/09 | CY125348 | North American |
| A_black_scoter_New_Brunswick_00003_2009 | H7N6 | 2/5/09 | CY129425 | North American |
| A_black_scoter_New_Brunswick_00010_2009 | H7N6 | 5/2/09 | CY125372 | North American |
| A_black_scoter_New_Brunswick_00014_2009 | H7N6 | 5/2/09 | CY125380 | North American |
| A_black-legged_kittiwake_Quebec_02838-1_2009 | H13N6 | 8/18/09 | CY125308 | North American |
| A_blue-winged_teal_Alberta_271_2007 | H10N7 | 8/4/07 | CY137713 | North American |
| A_blue-winged_teal_Alberta_346_2007 | H4N3 | 8/28/07 | CY103401 | North American |
| A_blue-winged_teal_Alberta_366_2007 | H3N8 | 8/28/07 | CY103409 | North American |
| A_blue-winged_teal_Alberta_376_2007 | H3N6 | 8/28/07 | CY101370 | North American |
| A_blue-winged_teal_Alberta_387_2007 | H3N8 | 8/28/07 | CY103442 | North American |
| A_blue-winged_teal_Alberta_405_2008 | H10N7 | 8/8/08 | CY136557 | North American |
| A_blue-winged_teal_Guatemala_CIP049-01_2008 | H7N9 | 2/7/08 | CY067667 | North American |
| A_blue-winged_teal_Guatemala_CIP049-02_2008 | H7N9 | 3/5/08 | CY067675 | North American |
| A_blue-winged_teal_Guatemala_CIP049-03_2009 | H11N2 | 11/11/09 | CY096637 | North American |
| A_blue-winged_teal_Guatemala_CIP049-04_2010 | H8N4 | 1/31/10 | CY096645 | North American |
| A_blue-winged_teal_Guatemala_CIP049-05_2010 | H3N8 | 1/31/10 | CY096653 | North American |
| A_blue-winged_teal_Guatemala_CIP049-06_2010 | H8N4 | 1/31/10 | CY096661 | North American |
| A_blue-winged_teal_Guatemala_CIP049-07_2010 | H8N4 | 1/31/10 | CY096693 | North American |
| A_blue-winged_teal_Guatemala_CIP049-08_2010 | H5N3 | 1/31/10 | CY096701 | North American |
| A_blue-winged_teal_Guatemala_CIP049-09_2010 | H5N3 | 1/31/10 | CY096717 | North American |
| A_blue-winged_teal_Guatemala_CIP049-10_2009 | H11N2 | 11/11/09 | CY096621 | North American |
| A_blue-winged_teal_Guatemala_CIP049-11_2009 | H11N2 | 11/11/09 | CY096629 | North American |
| A_blue-winged_teal_Guatemala_CIP049-12_2010 | H5N4 | 1/31/10 | CY096669 | North American |
| A_blue-winged_teal_Guatemala_CIP049-13_2010 | H5N4 | 1/31/10 | CY096677 | North American |
| A_blue-winged_teal_Guatemala_CIP049-14_2010 | H8N4 | 1/31/10 | CY096685 | North American |
| A_blue-winged_teal_Guatemala_CIP049-15_2010 | H8N4 | 1/31/10 | CY096709 | North American |
| A_blue-winged_teal_Illinois_10OS1546_2010 | H3N6 | 9/4/10 | CY132664 | North American |
| A_blue-winged_teal_Illinois_10OS1561_2010 | H4N6 | 9/4/10 | CY132908 | North American |
| A_blue-winged_teal_Illinois_10OS1563_2010 | H4N6 | 9/4/10 | CY132916 | North American |
| A_blue-winged_teal_Illinois_10OS2988_2010 | H4N6 | 9/19/10 | CY133632 | North American |
| A_blue-winged_teal_Illinois_10OS3610_2010 | H6N2 | 10/30/10 | CY132688 | North American |
| A_blue-winged_teal_Iowa_10OS2411_2010 | H3N8 | 9/18/10 | CY133576 | North American |
| A_blue-winged_teal_Iowa_10OS2624_2010 | H3N2 | 9/18/10 | CY133608 | North American |
| A_blue-winged_teal_Iowa_10OS2639_2010 | H3N8 | 9/18/10 | CY133616 | North American |
| A_blue-winged_teal_Iowa_10OS2649_2010 | H3N6 | 9/18/10 | CY133624 | North American |
| A_blue-winged_teal_Louisiana_AI09-4180_2009 | H4N6 | 9/5/09 | CY140934 | North American |
| A_blue-winged_teal_Louisiana_AI09-5159_2009 | H3N8 | 11/14/09 | CY140984 | North American |
| A_blue-winged_teal_Louisiana_AI09-5234_2009 | H11N2 | 11/15/09 | CY141000 | North American |
| A_blue-winged_teal_Louisiana_AI09-5291_2009 | H4N6 | 11/15/09 | CY141008 | North American |
| A_blue-winged_teal_Louisiana_Sg-00073_2007 | H10N7 | 9/15/07 | CY064192 | North American |
| A_blue-winged_teal_Louisiana_Sg-00224_2007 | H3N8 | 9/15/07 | CY078360 | North American |
| A_blue-winged_Teal_Minnesota_AI09-2389_2009 | H4N6 | 8/4/09 | CY140702 | North American |
| A_blue-winged_Teal_Minnesota_AI09-2977_2009 | H4N8 | 9/6/09 | CY140768 | North American |
| A_blue-winged_Teal_Minnesota_AI09-3575_2009 | H4N8 | 9/12/09 | CY140851 | North American |
| A_blue-winged_Teal_Minnesota_AI09-3786_2009 | H4N6 | 9/6/09 | CY140883 | North American |
| A_blue-winged_teal_Minnesota_Sg-00028_2007 | H4N6 | 8/2/07 | CY063750 | North American |
| A_blue-winged_teal_Minnesota_Sg-00029_2007 | H4N6 | 8/3/07 | CY063872 | North American |
| A_blue-winged_teal_Minnesota_Sg-00030_2007 | H4N6 | 8/3/07 | CY063880 | North American |
| A_blue-winged_teal_Minnesota_Sg-00031_2007 | H4N6 | 8/3/07 | CY063888 | North American |
| A_blue-winged_teal_Minnesota_Sg-00032_2007 | H4N6 | 8/3/07 | CY063896 | North American |
| A_blue-winged_teal_Minnesota_Sg-00034_2007 | H4N6 | 8/3/07 | CY063912 | North American |
| A_blue-winged_teal_Minnesota_Sg-00035_2007 | H4N6 | 8/3/07 | CY063920 | North American |
| A_blue-winged_teal_Minnesota_Sg-00036_2007 | H4N6 | 8/3/07 | CY063928 | North American |
| A_blue-winged_teal_Minnesota_Sg-00037_2007 | H3N8 | 8/3/07 | CY063936 | North American |
| A_blue-winged_teal_Minnesota_Sg-00038_2007 | H4N6 | 8/3/07 | CY063944 | North American |
| A_blue-winged_teal_Minnesota_Sg-00039_2007 | H4N6 | 8/3/07 | CY063952 | North American |
| A_blue-winged_teal_Minnesota_Sg-00040_2007 | H4N6 | 8/3/07 | CY063960 | North American |
| A_blue-winged_teal_Minnesota_Sg-00043_2007 | H4N6 | 8/3/07 | CY063984 | North American |
| A_blue-winged_teal_Minnesota_Sg-00201_2007 | H4N6 | 9/29/07 | CY078328 | North American |
| A_blue-winged_teal_Minnesota_Sg-00613_2008 | H3N8 | 7/29/08 | CY140268 | North American |
| A_blue-winged_teal_Minnesota_Sg-00649_2008 | H3N8 | 7/31/08 | CY042749 | North American |
| A_blue-winged_teal_Minnesota_Sg-00649_2008 | H3N8 | 7/31/08 | CY140077 | North American |
| A_blue-winged_teal_Minnesota_Sg-00787_2008 | H3N8 | 8/3/08 | CY140203 | North American |
| A_blue-winged_teal_Minnesota_Sg-00799_2008 | H4N6 | 9/1/08 | CY140211 | North American |
| A_blue-winged_teal_Minnesota_Sg-00899_2008 | H10N6 | 9/4/08 | CY145790 | North American |
| A_blue-winged_teal_Minnesota_Sg-01026_2008 | H4N6 | 9/11/08 | CY140534 | North American |
| A_blue-winged_teal_Missouri_10MO0011_2010 | H3N6 | 9/15/10 | CY133752 | North American |
| A_blue-winged_teal_Missouri_10MO003_2010 | H4N6 | 9/15/10 | CY133744 | North American |
| A_blue-winged_teal_Missouri_10MO013_2010 | H3N1 | 9/15/10 | CY133760 | North American |
| A_blue-winged_teal_Missouri_10MO021_2010 | H3N8 | 9/15/10 | CY133768 | North American |
| A_blue-winged_teal_Missouri_10MO030_2010 | H4N6 | 9/16/10 | CY133776 | North American |
| A_blue-winged_teal_Missouri_10MO0407_2010 | H4N6 | 9/17/10 | CY133858 | North American |
| A_blue-winged_teal_New_Brunswick_00283_2010 | H3N8 | 9/14/10 | CY138303 | North American |
| A_blue-winged_teal_New_Brunswick_00285_2010 | H3N8 | 9/14/10 | CY138311 | North American |
| A_blue-winged_teal_New_Brunswick_00288_2010 | H5N2 | 9/14/10 | CY138319 | North American |
| A_blue-winged_teal_New_Brunswick_00289_2010 | H3N8 | 9/14/10 | CY138327 | North American |
| A_blue-winged_teal_New_Brunswick_00291_2010 | H3N8 | 9/14/10 | CY138335 | North American |
| A_blue-winged_teal_New_Brunswick_00538_2010 | H3N8 | 8/13/10 | CY138343 | North American |
| A_blue-winged_teal_New_Brunswick_00595_2010 | H3N7 | 8/15/10 | CY138351 | North American |
| A_blue-winged_teal_New_Brunswick_00597_2010 | H3N7 | 8/15/10 | CY138359 | North American |
| A_blue-winged_teal_New_Brunswick_00598_2010 | H3N7 | 8/15/10 | CY138367 | North American |
| A_blue-winged_teal_New_Brunswick_00599_2010 | H3N7 | 8/15/10 | CY138375 | North American |
| A_blue-winged_teal_New_Brunswick_00600_2010 | H3N7 | 8/15/10 | CY138383 | North American |
| A_blue-winged_teal_New_Brunswick_00765_2010 | H3N8 | 8/25/10 | CY138391 | North American |
| A_blue-winged_teal_New_Brunswick_00766_2010 | H3N8 | 8/25/10 | CY138399 | North American |
| A_blue-winged_teal_New_Brunswick_00767_2010 | H3N8 | 8/25/10 | CY138407 | North American |
| A_blue-winged_teal_New_Brunswick_00768_2010 | H3N8 | 8/25/10 | CY138415 | North American |
| A_blue-winged_teal_New_Brunswick_00769_2010 | H3N8 | 8/25/10 | CY138423 | North American |
| A_blue-winged_teal_New_Brunswick_00770_2010 | H3N8 | 8/25/10 | CY138029 | North American |
| A_blue-winged_teal_New_Brunswick_00771_2010 | H3N8 | 8/25/10 | CY138431 | North American |
| A_blue-winged_teal_New_Brunswick_00772_2010 | H3N8 | 8/25/10 | CY138439 | North American |
| A_blue-winged_teal_New_Brunswick_00774_2010 | H3N8 | 8/25/10 | CY138447 | North American |
| A_blue-winged_teal_New_Brunswick_00775_2010 | H3N8 | 8/25/10 | CY138455 | North American |
| A_blue-winged_teal_New_Brunswick_03756_2009 | H4N2 | 9/14/09 | CY125588 | North American |
| A_blue-winged_teal_New_Brunswick_03757_2009 | H3N6 | 9/14/09 | CY125605 | North American |
| A_blue-winged_Teal_North_Dakota_AI09-2912_2009 | H6N1 | 9/2/09 | CY140760 | North American |
| A_blue-winged_Teal_North_Dakota_AI09-3131_2009 | H4N6 | 9/3/09 | CY140803 | North American |
| A_blue-winged_Teal_North_Dakota_AI09-3642_2009 | H3N1 | 9/4/09 | CY140867 | North American |
| A_blue-winged_Teal_North_Dakota_AI09-3760_2009 | H7N3 | 9/4/09 | CY140875 | North American |
| A_blue-winged_Teal_North_Dakota_AI09-3881_2009 | H4N6 | 9/13/09 | CY140910 | North American |
| A_blue-winged_Teal_North_Dakota_AI09-4039_2009 | H3N6 | 9/14/09 | CY140926 | North American |
| A_blue-winged_teal_North_Dakota_Sg-00706_2008 | H4N6 | 9/2/08 | CY140119 | North American |
| A_blue-winged_teal_North_Dakota_Sg-00719_2008 | H3N8 | 9/8/08 | CY140127 | North American |
| A_blue-winged_teal_North_Dakota_Sg-00730_2008 | H10N7 | 9/5/08 | CY140135 | North American |
| A_blue-winged_teal_North_Dakota_Sg-00733_2008 | H1N5 | 9/5/08 | CY140357 | North American |
| A_blue-winged_teal_North_Dakota_Sg-00735_2008 | H1N1 | 9/5/08 | CY140365 | North American |
| A_blue-winged_teal_North_Dakota_Sg-00740_2008 | H3N8 | 9/7/08 | CY140151 | North American |
| A_blue-winged_teal_North_Dakota_Sg-00750_2008 | H4N6 | 9/10/08 | CY140171 | North American |
| A_blue-winged_teal_North_Dakota_Sg-00759_2008 | H3N6 | 9/11/08 | CY140373 | North American |
| A_blue-winged_teal_Nova_Scotia_00069_2010 | H3N8 | 9/7/10 | CY138179 | North American |
| A_blue-winged_teal_Nova_Scotia_00071_2010 | H3N8 | 9/7/10 | CY138199 | North American |
| A_blue-winged_teal_Nova_Scotia_00072_2010 | H3N8 | 9/7/10 | CY138207 | North American |
| A_blue-winged_teal_Nova_Scotia_00073_2010 | H3N8 | 9/7/10 | CY138215 | North American |
| A_blue-winged_teal_Nova_Scotia_00074_2010 | H3N8 | 9/7/10 | CY138223 | North American |
| A_blue-winged_teal_Nova_Scotia_00075_2010 | H3N8 | 9/7/10 | CY138231 | North American |
| A_blue-winged_teal_Nova_Scotia_00076_2010 | H3N8 | 9/7/10 | CY138239 | North American |
| A_blue-winged_teal_Nova_Scotia_00077_2010 | H3N8 | 9/7/10 | CY138247 | North American |
| A_blue-winged_teal_Nova_Scotia_00078_2010 | H3N8 | 9/7/10 | CY138255 | North American |
| A_blue-winged_teal_Nova_Scotia_00079_2010 | H3N8 | 9/7/10 | CY138263 | North American |
| A_blue-winged_teal_Nova_Scotia_00080_2010 | H3N8 | 9/7/10 | CY138271 | North American |
| A_blue-winged_teal_Nova_Scotia_00081_2010 | H3N8 | 9/7/10 | CY138279 | North American |
| A_blue-winged_teal_Nova_Scotia_00121_2010 | H1N1 | 9/10/10 | CY138287 | North American |
| A_blue-winged_teal_Nova_Scotia_00126_2010 | H1N1 | 9/10/10 | CY138295 | North American |
| A_blue-winged_teal_Nova_Scotia_01000_2010 | H6N1 | 9/20/10 | CY138487 | North American |
| A_blue-winged_teal_Nova_Scotia_01002_2010 | H3N8 | 9/20/10 | CY138495 | North American |
| A_blue-winged_teal_Nova_Scotia_01003_2010 | H6N1 | 9/20/10 | CY138503 | North American |
| A_blue-winged_teal_Nova_Scotia_01008_2010 | H4N6 | 9/20/10 | CY138511 | North American |
| A_blue-winged_teal_Nova_Scotia_01009_2010 | H4N6 | 9/20/10 | CY138519 | North American |
| A_blue-winged_teal_Nova_Scotia_01027_2010 | H3N8 | 9/22/10 | CY138527 | North American |
| A_blue-winged_teal_Nova_Scotia_03971_2009 | H3N8 | 8/27/09 | CY125273 | North American |
| A_blue-winged_teal_Prince_Edward_Island_00803_2010 | H3N8 | 9/1/10 | CY138463 | North American |
| A_blue-winged_teal_Prince_Edward_Island_00831_2010 | H3N7 | 9/2/10 | CY138471 | North American |
| A_blue-winged_teal_Prince_Edward_Island_00844_2010 | H4N9 | 9/3/10 | CY138037 | North American |
| A_blue-winged_teal_Prince_Edward_Island_00849_2010 | H6N1 | 9/3/10 | CY138479 | North American |
| A_blue-winged_teal_Prince_Edward_Island_03910_2009 | H4N9 | 8/19/09 | CY125580 | North American |
| A_blue-winged_teal_Prince_Edward_Island_03912_2009 | H3N8 | 8/19/09 | CY125572 | North American |
| A_blue-winged_teal_Prince_Edward_Island_03927_2009 | H3N8 | 8/19/09 | CY125292 | North American |
| A_blue-winged_teal_Saskatchewan_22542_2007 | H5N2 | 8/18/07 | CY047711 | North American |
| A_blue-winged_Teal_Texas_AI09-3464_2009 | H4N8 | 9/13/09 | CY140843 | North American |
| A_blue-winged_teal_Texas_AI09-4405_2009 | H3N6 | 9/19/09 | CY140968 | North American |
| A_blue-winged_teal_Texas_AI09-4463_2009 | H4N8 | 9/20/09 | CY140976 | North American |
| A_blue-winged_teal_Texas_AI09-6182_2009 | H6N2 | 12/13/09 | CY141072 | North American |
| A_blue-winged_teal_Texas_Sg-00074_2007 | H4N8 | 9/15/07 | CY077708 | North American |
| A_blue-winged_teal_Texas_Sg-00076_2007 | H4N6 | 9/16/07 | CY064208 | North American |
| A_blue-winged_teal_Texas_Sg-00077_2007 | H4N6 | 9/15/07 | CY064216 | North American |
| A_blue-winged_teal_Texas_Sg-00078_2007 | H3N8 | 9/15/07 | CY064224 | North American |
| A_blue-winged_teal_Texas_Sg-00079_2007 | H3N8 | 9/15/07 | CY064232 | North American |
| A_blue-winged_teal_Texas_Sg-00080_2007 | H4N6 | 9/15/07 | CY064240 | North American |
| A_blue-winged_teal_Texas_Sg-00081_2007 | H4N6 | 9/15/07 | CY064248 | North American |
| A_blue-winged_teal_Texas_Sg-00085_2007 | H3N6 | 9/16/07 | CY078264 | North American |
| A_blue-winged_teal_Texas_Sg-00158_2007 | H4N6 | 9/15/07 | CY078288 | North American |
| A_blue-winged_teal_Texas_Sg-00173_2007 | H4N8 | 9/16/07 | CY090860 | North American |
| A_blue-winged_teal_Texas_Sg-00188_2007 | H4N8 | 9/15/07 | CY078312 | North American |
| A_blue-winged_teal_Texas_Sg-00206_2007 | H4N6 | 9/15/07 | CY078336 | North American |
| A_blue-winged_teal_Wisconsin_10OS2862_2010 | H3N2 | 10/2/10 | CY133108 | North American |
| A_blue-winged_teal_Wisconsin_10OS3092_2010 | H3N6 | 10/3/10 | CY133152 | North American |
| A_blue-winged_teal_Wisconsin_2509_2009 | H1N2 | 10/3/09 | CY097445 | North American |
| A_blue-winged_teal_Wisconsin_2572_2009 | H2N3 | 10/6/09 | CY097001 | North American |
| A_blue-winged_teal_Wisconsin_2649_2009 | H6N1 | 10/17/09 | CY097469 | North American |
| A_blue-winged_teal_Wisconsin_2665_2009 | H4N2 | 10/17/09 | CY097477 | North American |
| A_blue-winged_teal_Wisconsin_2713_2009 | H4N2 | 10/17/09 | CY097025 | North American |
| A_blue-winged_teal_Wisconsin_2720_2009 | H3N2 | 10/17/09 | CY097493 | North American |
| A_blue-winged_teal_Wisconsin_2741_2009 | H4N6 | 10/17/09 | CY097033 | North American |
| A_blue-winged_teal_Wisconsin_2753_2009 | H4N6 | 10/24/09 | CY097049 | North American |
| A_blue-winged_teal_Wisconsin_3060_2009 | H3N2 | 10/17/09 | CY097218 | North American |
| A_bufflehead_Alberta_400_2007 | H3N8 | 8/28/07 | CY103450 | North American |
| A_bufflehead_California_8522_2008 | H4N8 | 10/25/08 | CY093869 | North American |
| A_bufflehead_California_HKWF205_2007 | H4N8 | 10/28/07 | CY032889 | North American |
| A_bufflehead_California_JN1016_2006 | H2N9 | 12/6/06 | CY076847 | North American |
| A_bufflehead_Illinois_4016_2009 | H4N8 | 11/28/09 | CY096977 | North American |
| A_bufflehead_Wisconsin_10OS3204_2010 | H12N5 | 11/7/10 | CY133116 | North American |
| A_California_gull_Washington_20371-003_2007 | H10N7 | 7/24/07 | CY076124 | North American |
| A_Canada_goose_Delaware_Bay_34_2010 | H6N1 | 5/19/10 | CY127920 | North American |
| A_chicken_New_York_19495-3_2006 | H7N2 | 2/21/06 | CY034917 | North American |
| A_chicken_New_York_19495-4_2006 | H7N2 | 2/21/06 | CY035993 | North American |
| A_chicken_New_York_19495-5_2006 | H7N2 | 2/21/06 | CY047010 | North American |
| A_chicken_New_York_19499-1_2006 | H7N2 | 2/17/06 | CY036017 | North American |
| A_cinnamon_teal_California_44287-234_2007 | H11N9 | 1/27/07 | CY076412 | North American |
| A_cinnamon_teal_California_44287-325_2007 | H3N8 | 1/27/07 | CY076020 | North American |
| A_cinnamon_teal_California_44287-659_2007 | H10N3 | 2/3/07 | CY076468 | North American |
| A_cinnamon_teal_California_HKWF1111C_2007 | H5N7 | 12/2/07 | CY094444 | North American |
| A_common_goldeneye_Iowa_3192_2009 | H11N9 | 11/7/09 | CY097073 | North American |
| A_common_goldeneye_Wisconsin_10OS4202_2010 | H7N6 | 12/2/10 | CY133136 | North American |
| A_common_murre_Oregon_20361-001_2007 | H10N7 | 7/24/07 | CY076108 | North American |
| A_common_murre_Oregon_20361-002_2007 | H12N5 | 7/24/07 | CY076116 | North American |
| A_common_teal_Netherlands_1_2005 | H8N4 | 2005 | CY041265 | North American |
| A_duck_Interior_Alaska_7MP1550_2007 | H4N6 | 9/1/07 | CY080198 | North American |
| A_duck_Interior_Alaska_7MP1570_2007 | H4N6 | 9/1/07 | CY078986 | North American |
| A_duck_Interior_Alaska_7MP1582_2007 | H1N1 | 9/1/07 | CY078994 | North American |
| A_duck_Interior_Alaska_7MP1591R1_2007 | H3N8 | 9/1/07 | CY078954 | North American |
| A_duck_Interior_Alaska_7MP1598_2007 | H3N8 | 9/1/07 | CY079002 | North American |
| A_gadwall_Altai_1326_2007 | H3N8 | 2007/09/ | CY049801 | North American |
| A_gadwall_California_29595_2007 | H10N7 | 1/28/07 | CY076452 | North American |
| A_gadwall_California_44287-543_2007 | H10N7 | 1/28/07 | CY076460 | North American |
| A_gadwall_California_8340_2008 | H6N1 | 10/19/08 | CY093925 | North American |
| A_gadwall_California_8504_2008 | H6N1 | 10/25/08 | CY093933 | North American |
| A_gadwall_California_8535_2008 | H6N1 | 10/25/08 | CY094484 | North American |
| A_gadwall_California_8708_2008 | H6N1 | 11/1/08 | CY094492 | North American |
| A_gadwall_California_9155_2008 | H6N1 | 11/19/08 | CY093948 | North American |
| A_gadwall_California_AKS-514_2007 | H6N1 | 12/9/07 | CY093956 | North American |
| A_gadwall_California_HKWF100_2007 | H6N1 | 10/24/07 | CY032661 | North American |
| A_gadwall_duck_Minnesota_Sg-00575_2008 | H3N8 | 7/31/08 | CY042624 | North American |
| A_gadwall_duck_Minnesota_Sg-00575_2008 | H3N8 | 7/31/08 | CY140012 | North American |
| A_gadwall_Illinois_10OS3342_2010 | H6N1 | 10/30/10 | CY132988 | North American |
| A_gadwall_Illinois_10OS3384_2010 | H11N2 | 10/30/10 | CY133012 | North American |
| A_gadwall_Illinois_3860_2009 | H6N1 | 11/15/09 | CY097525 | North American |
| A_gadwall_Iowa_10OS2753_2010 | H3N8 | 10/17/10 | CY133479 | North American |
| A_gadwall_Mississippi_10OS4531_2010 | H6N2 | 12/11/10 | CY133289 | North American |
| A_gadwall_Missouri_10MO0280_2010 | H5N2 | 11/16/10 | CY133816 | North American |
| A_gadwall_Missouri_10MO095_2010 | H6N1 | 11/16/10 | CY133800 | North American |
| A_gadwall_Missouri_10OS4731_2010 | H7N3 | 12/12/10 | CY133415 | North American |
| A_gadwall_Nova_Scotia_00783_2010 | H3N8 | 9/7/10 | CY139270 | North American |
| A_gadwall_Nova_Scotia_00786_2010 | H3N8 | 9/7/10 | CY139278 | North American |
| A_gadwall_Wisconsin_08OS2293_2008 | H3N2 | 10/20/08 | CY079355 | North American |
| A_gadwall_Wisconsin_08OS2296_2008 | H6N2 | 10/20/08 | CY079443 | North American |
| A_garganey_Altai_1216_2007 | H3N6 | 2007/09/ | CY049777 | North American |
| A_glaucous-winged_gull_Southeastern_Alaska_10JR01572R0_2010 | H16N3 | 8/13/10 | CY130500 | North American |
| A_glaucous-winged_gull_Southeastern_Alaska_10JR01681R0_2010 | H16N3 | 9/8/10 | CY130508 | North American |
| A_glaucous-winged_gull_Southeastern_Alaska_10JR01700R0_2010 | H16N3 | 9/8/10 | CY130516 | North American |
| A_glaucous-winged_gull_Southeastern_Alaska_10JR01811R0_2010 | H16N3 | 9/9/10 | CY130524 | North American |
| A_glaucous-winged_gull_Southeastern_Alaska_10JR01856R0_2010 | H11N9 | 9/9/10 | CY125749 | North American |
| A_glaucous-winged_gull_Southeastern_Alaska_9JR0822R0_2009 | H13N6 | 8/19/09 | CY130347 | North American |
| A_greater_scaup_Wisconsin_4234_2009 | H11N9 | 12/6/09 | CY097316 | North American |
| A_greater_white-fronted_goose_California_10936_2008 | H1N1 | 11/29/08 | CY093980 | North American |
| A_greater_white-fronted_goose_California_44358-076_2007 | H6N1 | 10/27/07 | CY076652 | North American |
| A_greater_white-fronted_goose_California_44358-077_2007 | H6N1 | 10/27/07 | CY076084 | North American |
| A_greater_white-fronted_goose_California_44358-089_2007 | H6N1 | 10/27/07 | CY076660 | North American |
| A_greater_white-fronted_goose_California_44358-095_2007 | H6N1 | 10/27/07 | CY076668 | North American |
| A_greater_white-fronted_goose_California_44358-112_2007 | H6N1 | 11/4/07 | CY076676 | North American |
| A_greater_white-fronted_goose_California_6365_2008 | H6N1 | 10/29/08 | CY094500 | North American |
| A_greater_white-fronted_goose_California_6461_2008 | H6N1 | 11/2/08 | CY093964 | North American |
| A_greater_white-fronted_goose_California_6548_2008 | H6N1 | 11/5/08 | CY093972 | North American |
| A_greater_white-fronted_goose_California_AKS617_2007 | H6N1 | 12/12/07 | CY039561 | North American |
| A_greater_white-fronted_goose_California_HKWF446C_2007 | H10N7 | 11/4/07 | CY094508 | North American |
| A_green_winged_teal_California_AKS1305_2008 | H11N9 | 1/26/08 | CY039529 | North American |
| A_green_winged_teal_California_AKS1370_2008 | H7N3 | 1/27/08 | CY039537 | North American |
| A_green-winged_teal_California_00083_2009 | H10N7 | 1/3/09 | CY093917 | North American |
| A_green-winged_teal_California_10197_2008 | H10N7 | 12/14/08 | CY093893 | North American |
| A_green-winged_teal_California_11275_2008 | H7N3 | 12/13/08 | CY094468 | North American |
| A_green-winged_teal_California_1841_2009 | H7N3 | 1/18/09 | CY094580 | North American |
| A_green-winged_teal_California_6990_2008 | H6N1 | 11/23/08 | CY093877 | North American |
| A_green-winged_teal_California_7972_2008 | H6N2 | 10/25/08 | CY093885 | North American |
| A_green-winged_teal_California_8326_2008 | H1N2 | 10/19/08 | CY094452 | North American |
| A_green-winged_teal_California_8612_2008 | H6N1 | 10/29/08 | CY094460 | North American |
| A_green-winged_teal_California_K481_2006 | H1N3 | 1/7/06 | CY053820 | North American |
| A_green-winged_teal_Interior_Alaska_1_2007 | H3N8 | 8/11/07 | CY036656 | North American |
| A_green-winged_teal_Interior_Alaska_6MP0736_2006 | H3N8 | 8/7/06 | CY078442 | North American |
| A_green-winged_teal_Interior_Alaska_6MP0909_2006 | H3N8 | 8/17/06 | CY078482 | North American |
| A_green-winged_teal_Interior_Alaska_6MP0936R1_2006 | H3N8 | 8/17/06 | CY078658 | North American |
| A_green-winged_teal_Interior_Alaska_6MP1077_2006 | H3N8 | 8/20/06 | CY078546 | North American |
| A_green-winged_teal_Interior_Alaska_6MP1140_2006 | H3N8 | 9/22/06 | CY079739 | North American |
| A_green-winged_teal_Interior_Alaska_6MP1312_2006 | H3N8 | 9/3/06 | CY078530 | North American |
| A_green-winged_teal_Interior_Alaska_6MP1330_2006 | H3N8 | 9/8/06 | CY078554 | North American |
| A_green-winged_teal_Louisiana_Sg-00092_2007 | H3N8 | 9/15/07 | CY078272 | North American |
| A_green-winged_teal_Minnesota_Sg-00180_2007 | H6N2 | 9/14/07 | CY078304 | North American |
| A_green-winged_teal_Minnesota_Sg-00199_2007 | H6N2 | 9/14/07 | CY078320 | North American |
| A_green-winged_teal_Minnesota_Sg-00222_2007 | H6N2 | 9/16/07 | CY078352 | North American |
| A_green-winged_teal_Minnesota_Sg-00820_2008 | H4N5 | 9/3/08 | CY140381 | North American |
| A_green-winged_teal_Minnesota_Sg-00991_2008 | H3N8 | 9/11/08 | CY140498 | North American |
| A_green-winged_teal_Minnesota_Sg-01000_2008 | H3N6 | 9/11/08 | CY140506 | North American |
| A_green-winged_teal_Minnesota_Sg-01065_2008 | H6N2 | 10/11/08 | CY140614 | North American |
| A_green-winged_teal_Minnesota_Sg-01073_2008 | H6N2 | 11/9/08 | CY140638 | North American |
| A_green-winged_teal_New_Brunswick_00333_2010 | H1N1 | 8/8/10 | CY138720 | North American |
| A_green-winged_teal_New_Brunswick_00335_2010 | H3N8 | 8/8/10 | CY138728 | North American |
| A_green-winged_teal_New_Brunswick_00336_2010 | H3N8 | 8/8/10 | CY138736 | North American |
| A_green-winged_teal_New_Brunswick_00392_2010 | H7N7 | 8/9/10 | CY138841 | North American |
| A_green-winged_teal_New_Brunswick_02426_2007 | H4N6 | 8/27/07 | CY125564 | North American |
| A_green-winged_teal_New_Brunswick_02586_2007 | H3N8 | 9/20/07 | CY125444 | North American |
| A_green-winged_teal_New_Brunswick_02587_2007 | H3N8 | 9/20/07 | CY125524 | North American |
| A_green-winged_teal_New_Brunswick_02588_2007 | H3N8 | 9/20/07 | CY125492 | North American |
| A_green-winged_teal_New_Brunswick_02590_2007 | H3N8 | 9/20/07 | CY125436 | North American |
| A_green-winged_teal_New_Brunswick_02591_2007 | H3N8 | 9/20/07 | CY125428 | North American |
| A_green-winged_teal_New_Brunswick_02592_2007 | H3N8 | 9/20/07 | CY125484 | North American |
| A_green-winged_teal_New_Brunswick_02593_2007 | H3N8 | 9/20/07 | CY125508 | North American |
| A_green-winged_teal_New_Brunswick_02595_2007 | H3N8 | 9/20/07 | CY125412 | North American |
| A_green-winged_teal_New_Brunswick_02596_2007 | H3N8 | 9/20/07 | CY125420 | North American |
| A_green-winged_teal_New_Brunswick_02597_2007 | H3N8 | 9/20/07 | CY125500 | North American |
| A_green-winged_teal_New_Brunswick_02600_2007 | H4N6 | 9/21/07 | CY125227 | North American |
| A_green-winged_teal_New_Brunswick_02600_2007 | H4N6 | 9/21/07 | CY125228 | North American |
| A_green-winged_teal_New_Brunswick_02601_2007 | H4N6 | 9/21/07 | CY125236 | North American |
| A_green-winged_teal_New_Brunswick_02601_2007 | H4N6 | 9/21/07 | CY125237 | North American |
| A_green-winged_teal_New_Brunswick_02630_2007 | H4N6 | 9/22/07 | CY125404 | North American |
| A_green-winged_teal_New_Brunswick_03483_2009 | H3N8 | 8/14/09 | CY125388 | North American |
| A_green-winged_teal_Nova_Scotia_00354_2010 | H3N8 | 8/8/10 | CY138800 | North American |
| A_green-winged_teal_Prince_Edward_Island_00838_2010 | H3N8 | 9/2/10 | CY139294 | North American |
| A_green-winged_teal_Prince_Edward_Island_00839_2010 | H3N8 | 9/2/10 | CY139302 | North American |
| A_green-winged_teal_Prince_Edward_Island_00840_2010 | H3N8 | 9/2/10 | CY139310 | North American |
| A_green-winged_teal_Prince_Edward_Island_00841_2010 | H3N8 | 9/2/10 | CY139318 | North American |
| A_Guinea_fowl_New_York_19495-6_2006 | H7N2 | 2/21/06 | CY036001 | North American |
| A_Guinea_fowl_New_York_32084_2006 | H7N2 | 7/26/06 | CY034245 | North American |
| A_gull_Delaware_AI09-438_2009 | H1N1 | 5/28/09 | CY146003 | North American |
| A_gull_Delaware_AI09-453_2009 | H1N1 | 5/28/09 | CY146011 | North American |
| A_gull_New_Jersey_AI09-964_2009 | H1N8 | 5/27/09 | CY146253 | North American |
| A_gull_Southeastern_Alaska_10JR01527R0_2010 | H16N3 | 8/12/10 | CY130492 | North American |
| A_herring_gull_New_Jersey_AI09-1262_2009 | H10N7 | 6/10/09 | CY146375 | North American |
| A_herring_gull_New_Jersey_AI09-335_2009 | H11N1 | 5/16/09 | CY145962 | North American |
| A_hooded_merganser_New_Brunswick_03749_2009 | H3N8 | 9/14/09 | CY125332 | North American |
| A_hooded_merganser_New_Brunswick_03750_2009 | H13N6 | 9/14/09 | CY125316 | North American |
| A_laughing_gull_Delaware_Bay_42_2006 | H7N3 | 5/22/06 | CY102974 | North American |
| A_laughing_gull_Delaware_Bay_46_2006 | H7N3 | 5/22/06 | CY037094 | North American |
| A_laughing_gull_Delaware_Bay_6_2006 | H7N3 | 5/22/06 | CY037086 | North American |
| A_laughing_gull_New_Jersey_AI09-218_2009 | H11N1 | 5/15/09 | CY145922 | North American |
| A_laughing_gull_New_Jersey_AI09-271_2009 | H11N1 | 5/15/09 | CY145938 | North American |
| A_laughing_gull_New_Jersey_Sg-00485_2008 | H13N9 | 5/15/08 | CY042427 | North American |
| A_laughing_gull_New_Jersey_Sg-00485_2008 | H13N9 | 5/15/08 | CY144988 | North American |
| A_laughing_gull_New_Jersey_Sg-00559_2008 | H13N9 | 5/28/08 | CY042590 | North American |
| A_laughing_gull_New_Jersey_Sg-00559_2008 | H13N9 | 5/28/08 | CY145460 | North American |
| A_laughing_gull_New_Jersey_Sg-00568_2008 | H13N9 | 5/28/08 | CY042604 | North American |
| A_laughing_gull_New_Jersey_Sg-00568_2008 | H13N9 | 5/28/08 | CY140260 | North American |
| A_least_sandpiper_Alaska_7KW0411_2007 | H4N8 | 5/10/07 | CY044007 | North American |
| A_least_sandpiper_South_Central_Alaska_2_2007 | H4N8 | 5/9/07 | CY035780 | North American |
| A_least_sandpiper_South_Central_Alaska_3_2007 | H4N8 | 5/9/07 | CY035788 | North American |
| A_least_sandpiper_South_Central_Alaska_7KW0434_2007 | H4N8 | 5/10/07 | CY077332 | North American |
| A_lesser_scaup_Illinois_4115_2009 | H10N7 | 11/29/09 | CY097533 | North American |
| A_lesser_scaup_Louisiana_AI09-5204_2009 | H6N8 | 11/14/09 | CY140992 | North American |
| A_lesser_scaup_Wisconsin_3964_2009 | H10N3 | 11/23/09 | CY097300 | North American |
| A_long-tailed_duck_Wisconsin_10OS3915_2010 | H3N6 | 11/16/10 | CY133552 | North American |
| A_long-tailed_duck_Wisconsin_10OS3918_2010 | H14N8 | 11/16/10 | CY133560 | North American |
| A_long-tailed_duck_Wisconsin_10OS3919_2010 | H10N6 | 11/16/10 | CY133568 | North American |
| A_mallard_Alaska_7MP1028_2007 | H4N8 | 9/7/07 | CY043975 | North American |
| A_mallard_Alberta_107_2007 | H3N8 | 8/22/07 | CY103226 | North American |
| A_mallard_Alberta_114_2007 | H4N6 | 8/22/07 | CY103243 | North American |
| A_mallard_Alberta_116_2007 | H3N8 | 8/22/07 | CY103264 | North American |
| A_mallard_Alberta_121_2008 | H4N6 | 8/6/08 | CY103474 | North American |
| A_mallard_Alberta_122_2007 | H3N8 | 8/22/07 | CY103272 | North American |
| A_mallard_Alberta_128_2006 | H1N1 | 8/3/06 | CY137665 | North American |
| A_mallard_Alberta_130_2007 | H3N8 | 8/22/07 | CY103288 | North American |
| A_mallard_Alberta_134_2007 | H3N8 | 8/22/07 | CY103297 | North American |
| A_mallard_Alberta_137_2007 | H3N8 | 8/22/07 | CY103305 | North American |
| A_mallard_Alberta_145_2007 | H4N6 | 8/22/07 | CY103313 | North American |
| A_mallard_Alberta_152_2006 | H1N3 | 8/3/06 | CY137673 | North American |
| A_mallard_Alberta_155_2009 | H1N9 | 8/19/09 | CY137997 | North American |
| A_mallard_Alberta_160_2007 | H4N6 | 8/22/07 | CY103331 | North American |
| A_mallard_Alberta_162_2007 | H12N5 | 8/3/07 | CY077189 | North American |
| A_mallard_Alberta_166_2006 | H1N1 | 8/4/06 | CY137681 | North American |
| A_mallard_Alberta_182_2007 | H10N7 | 8/7/07 | CY137705 | North American |
| A_mallard_Alberta_221_2006 | H12N6 | 8/9/06 | CY077165 | North American |
| A_mallard_Alberta_221_2006 | H12N6 | 8/9/06 | CY103077 | North American |
| A_mallard_Alberta_224_2006 | H12N5 | 8/9/06 | CY077173 | North American |
| A_mallard_Alberta_224_2006 | H12N5 | 8/9/06 | CY103085 | North American |
| A_mallard_Alberta_225_2006 | H3N8 | 8/9/06 | CY103093 | North American |
| A_mallard_Alberta_228_2006 | H3N8 | 8/9/06 | CY103101 | North American |
| A_mallard_Alberta_234_2007 | H12N5 | 8/3/07 | CY077197 | North American |
| A_mallard_Alberta_234_2007 | H12N5 | 8/3/07 | CY103339 | North American |
| A_mallard_Alberta_238_2006 | H3N8 | 8/9/06 | CY103109 | North American |
| A_mallard_Alberta_243_2006 | H7N3 | 8/9/06 | CY103117 | North American |
| A_mallard_Alberta_246_2006 | H4N6 | 8/9/06 | CY103125 | North American |
| A_mallard_Alberta_254_2006 | H4N6 | 8/9/06 | CY103133 | North American |
| A_mallard_Alberta_258_2006 | H4N6 | 8/9/06 | CY103153 | North American |
| A_mallard_Alberta_27_2007 | H3N8 | 8/18/07 | CY103193 | North American |
| A_mallard_Alberta_270_2008 | H4N6 | 8/7/08 | CY103482 | North American |
| A_mallard_Alberta_274_2006 | H3N8 | 8/9/06 | CY103161 | North American |
| A_mallard_Alberta_279_2006 | H3N8 | 8/9/06 | CY103169 | North American |
| A_mallard_Alberta_28_2007 | H3N8 | 8/18/07 | CY103201 | North American |
| A_mallard_Alberta_289_2007 | H10N7 | 8/4/07 | CY137721 | North American |
| A_mallard_Alberta_289_2009 | H2N9 | 8/20/09 | CY117404 | North American |
| A_mallard_Alberta_297_2006 | H4N6 | 8/9/06 | CY103177 | North American |
| A_mallard_Alberta_312_2007 | H3N8 | 8/28/07 | CY103367 | North American |
| A_mallard_Alberta_318_2007 | H3N8 | 8/28/07 | CY103375 | North American |
| A_mallard_Alberta_319_2009 | H2N3 | 8/22/09 | CY117420 | North American |
| A_mallard_Alberta_330_2007 | H4N6 | 8/28/07 | CY103383 | North American |
| A_mallard_Alberta_34_2007 | H3N8 | 8/18/07 | CY103209 | North American |
| A_mallard_Alberta_35_2009 | H2N3 | 8/19/09 | CY117412 | North American |
| A_mallard_Alberta_417_2009 | H2N3 | 8/22/09 | CY103526 | North American |
| A_mallard_Alberta_419_2009 | H2N3 | 8/22/09 | CY117428 | North American |
| A_mallard_Alberta_421_2009 | H2N3 | 8/22/09 | CY103534 | North American |
| A_mallard_Alberta_496_2008 | H1N1 | 8/8/08 | CY137753 | North American |
| A_mallard_Alberta_507_2008 | H3N8 | 8/8/08 | CY103490 | North American |
| A_mallard_Alberta_527_2008 | H3N8 | 8/8/08 | CY103498 | North American |
| A_mallard_Alberta_551_2009 | H3N8 | 8/23/09 | CY103542 | North American |
| A_mallard_Alberta_569_2008 | H1N1 | 8/8/08 | CY137761 | North American |
| A_mallard_Alberta_66_2007 | H1N1 | 8/18/07 | CY137689 | North American |
| A_mallard_Alberta_76_2006 | H1N3 | 7/26/06 | CY077157 | North American |
| A_mallard_Alberta_80_2006 | H1N3 | 7/26/06 | CY137657 | North American |
| A_mallard_Alberta_91_2007 | H1N5 | 8/22/07 | CY137697 | North American |
| A_mallard_Altai_1208_2007 | H3N6 | 2007/09/ | CY049761 | North American |
| A_mallard_Arkansas_AI09-5649_2009 | H9N2 | 12/11/09 | CY146520 | North American |
| A_mallard_Arkansas_AI09-5663_2009 | H11N9 | 12/10/09 | CY141016 | North American |
| A_mallard_Arkansas_AI09-5761_2009 | H2N3 | 12/11/09 | CY141024 | North American |
| A_mallard_Arkansas_AI09-5900_2009 | H3N8 | 12/11/09 | CY141032 | North American |
| A_mallard_Arkansas_AI09-5944_2009 | H4N8 | 12/10/09 | CY141040 | North American |
| A_mallard_Arkansas_AI09-6032_2009 | H10N9 | 12/11/09 | CY141056 | North American |
| A_mallard_California_10064_2008 | H1N6 | 12/21/08 | CY094244 | North American |
| A_mallard_California_10125_2008 | H11N9 | 12/13/08 | CY093678 | North American |
| A_mallard_California_10126_2008 | H10N7 | 12/13/08 | CY093686 | North American |
| A_mallard_California_11095_2008 | H4N6 | 12/6/08 | CY094252 | North American |
| A_mallard_California_11100_2008 | H11N2 | 12/6/08 | CY093709 | North American |
| A_mallard_California_11119_2008 | H11N9 | 12/6/08 | CY093717 | North American |
| A_mallard_California_11353_2008 | H10N2 | 12/13/08 | CY094260 | North American |
| A_mallard_California_1154_2010 | H4N6 | 7/27/10 | CY125957 | North American |
| A_mallard_California_1156_2010 | H4N6 | 7/27/10 | CY094756 | North American |
| A_mallard_California_1188_2010 | H4N6 | 7/29/10 | CY094780 | North American |
| A_mallard_California_1210_2010 | H4N6 | 7/29/10 | CY094764 | North American |
| A_mallard_California_1289_2010 | H4N6 | 7/30/10 | CY094788 | North American |
| A_mallard_California_1297_2010 | H4N6 | 7/30/10 | CY120690 | North American |
| A_mallard_California_1305_2010 | H10N7 | 8/3/10 | CY094772 | North American |
| A_mallard_California_1335_2010 | H10N7 | 8/3/10 | CY120698 | North American |
| A_mallard_California_1335-1_2010 | H10N7 | 8/3/10 | CY120706 | North American |
| A_mallard_California_1353_2010 | H10N7 | 8/6/10 | CY094796 | North American |
| A_mallard_California_1356_2010 | H10N7 | 8/4/10 | CY120554 | North American |
| A_mallard_California_1390_2010 | H7N5 | 8/4/10 | CY120562 | North American |
| A_mallard_California_1438_2010 | H2N3 | 8/9/10 | CY120570 | North American |
| A_mallard_California_1492_2010 | H6N4 | 8/10/10 | CY120578 | North American |
| A_mallard_California_1523_2010 | H4N6 | 8/11/10 | CY120586 | North American |
| A_mallard_California_20385-002_2007 | H1N1 | 8/5/07 | CY076132 | North American |
| A_mallard_California_20385-004_2007 | H1N1 | 8/5/07 | CY076140 | North American |
| A_mallard_California_2396_2010 | H5N2 | 10/30/10 | CY120594 | North American |
| A_mallard_California_2420_2010 | H10N7 | 10/23/10 | CY133900 | North American |
| A_mallard_California_2444_2010 | H6N1 | 10/27/10 | CY120602 | North American |
| A_mallard_California_2529_2010 | H6N1 | 10/23/10 | CY134390 | North American |
| A_mallard_California_3134_2010 | H1N1 | 11/24/10 | CY120618 | North American |
| A_mallard_California_3188_2010 | H6N8 | 11/27/10 | CY120650 | North American |
| A_mallard_California_3569_2010 | H1N3 | 12/18/10 | CY134406 | North American |
| A_mallard_California_5149_2009 | H5N2 | 7/25/09 | CY094660 | North American |
| A_mallard_California_5174_2009 | H5N2 | 7/25/09 | CY094668 | North American |
| A_mallard_California_5191_2009 | H5N2 | 7/27/09 | CY094740 | North American |
| A_mallard_California_5192_2009 | H4N2 | 7/27/09 | CY094676 | North American |
| A_mallard_California_5197_2009 | H5N2 | 7/27/09 | CY120714 | North American |
| A_mallard_California_5212_2009 | H5N2 | 7/27/09 | CY094588 | North American |
| A_mallard_California_5219_2009 | H5N2 | 7/27/09 | CY094020 | North American |
| A_mallard_California_5222_2009 | H5N2 | 7/27/09 | CY094596 | North American |
| A_mallard_California_5250_2009 | H5N2 | 7/28/09 | CY094604 | North American |
| A_mallard_California_5255_2009 | H3N8 | 7/28/09 | CY094692 | North American |
| A_mallard_California_5271_2009 | H4N6 | 7/29/09 | CY094612 | North American |
| A_mallard_California_5276_2009 | H5N2 | 7/29/09 | CY094620 | North American |
| A_mallard_California_5296_2009 | H5N2 | 7/29/09 | CY094636 | North American |
| A_mallard_California_5319_2009 | H5N2 | 7/23/09 | CY094700 | North American |
| A_mallard_California_5351_2009 | H1N1 | 8/4/09 | CY094708 | North American |
| A_mallard_California_5359_2009 | H5N2 | 8/4/09 | CY094716 | North American |
| A_mallard_California_5386_2009 | H5N2 | 8/4/09 | CY094724 | North American |
| A_mallard_California_5491_2009 | H5N2 | 7/23/09 | CY094732 | North American |
| A_mallard_California_5495_2009 | H4N2 | 7/23/09 | CY094644 | North American |
| A_mallard_California_5502_2009 | H5N2 | 7/23/09 | CY094652 | North American |
| A_mallard_California_6404_2008 | H6N1 | 11/1/08 | CY094092 | North American |
| A_mallard_California_6420_2008 | H6N2 | 11/1/08 | CY094100 | North American |
| A_mallard_California_6469_2008 | H6N2 | 11/2/08 | CY093598 | North American |
| A_mallard_California_6471_2008 | H3N8 | 11/2/08 | CY093606 | North American |
| A_mallard_California_6490_2008 | H12N5 | 11/2/08 | CY094108 | North American |
| A_mallard_California_6517_2008 | H11N9 | 11/5/08 | CY094116 | North American |
| A_mallard_California_6524_2008 | H12N5 | 11/5/08 | CY094124 | North American |
| A_mallard_California_6634_2008 | H11N9 | 11/9/08 | CY094132 | North American |
| A_mallard_California_6695_2009 | H6N1 | 10/25/09 | CY094572 | North American |
| A_mallard_California_6744_2009 | H6N1 | 10/25/09 | CY094548 | North American |
| A_mallard_California_6768_2009 | H1N1 | 10/25/09 | CY094556 | North American |
| A_mallard_California_6772_2008 | H4N6 | 11/15/08 | CY094140 | North American |
| A_mallard_California_6957_2008 | H10N7 | 11/22/08 | CY094148 | North American |
| A_mallard_California_7766_2008 | H4N6 | 10/8/08 | CY039736 | North American |
| A_mallard_California_8028_2008 | H11N2 | 10/18/08 | CY094156 | North American |
| A_mallard_California_8035_2008 | H5N2 | 10/18/08 | CY094164 | North American |
| A_mallard_California_8118_2008 | H11N9 | 10/19/08 | CY093614 | North American |
| A_mallard_California_8212_2008 | H6N1 | 10/18/08 | CY094172 | North American |
| A_mallard_California_8293_2008 | H6N1 | 10/18/08 | CY094180 | North American |
| A_mallard_California_8322_2008 | H6N1 | 10/19/08 | CY093622 | North American |
| A_mallard_California_8399_2008 | H6N1 | 10/22/08 | CY093630 | North American |
| A_mallard_California_8416_2008 | H6N1 | 10/22/08 | CY094196 | North American |
| A_mallard_California_8427_2008 | H6N1 | 10/22/08 | CY094204 | North American |
| A_mallard_California_8429_2008 | H6N1 | 10/22/08 | CY094212 | North American |
| A_mallard_California_8432_2008 | H6N1 | 10/22/08 | CY094220 | North American |
| A_mallard_California_8457_2008 | H6N2 | 10/22/08 | CY093638 | North American |
| A_mallard_California_8462_2008 | H6N1 | 10/22/08 | CY093646 | North American |
| A_mallard_California_8518_2008 | H6N1 | 10/25/08 | CY094228 | North American |
| A_mallard_California_8519_2008 | H6N1 | 10/25/08 | CY093654 | North American |
| A_mallard_California_8834_2008 | H5N9 | 10/19/08 | CY094188 | North American |
| A_mallard_California_8843_2008 | H1N1 | 11/5/08 | CY093662 | North American |
| A_mallard_California_9573_2008 | H4N6 | 11/30/08 | CY094236 | North American |
| A_mallard_California_9704_2008 | H10N7 | 12/7/08 | CY093670 | North American |
| A_mallard_California_AKS478_2007 | H10N7 | 12/8/07 | CY094268 | North American |
| A_mallard_California_K752_2006 | H10N7 | 2/4/06 | CY053812 | North American |
| A_mallard_Illinois_08OS2315_2008 | H4N6 | 10/25/08 | CY079451 | North American |
| A_mallard_Illinois_08OS2710_2008 | H10N7 | 10/26/08 | CY079363 | North American |
| A_mallard_Illinois_08OS2711_2008 | H10N7 | 10/26/08 | CY079467 | North American |
| A_mallard_Illinois_10OS2677_2010 | H3N2 | 10/2/10 | CY132704 | North American |
| A_mallard_Illinois_10OS3249_2010 | H11N2 | 10/29/10 | CY132866 | North American |
| A_mallard_Illinois_10OS3599_2010 | H6N1 | 10/30/10 | CY133028 | North American |
| A_mallard_Illinois_10OS3659_2010 | H11N2 | 10/31/10 | CY133528 | North American |
| A_mallard_Illinois_10OS3676_2010 | H3N8 | 10/31/10 | CY133544 | North American |
| A_mallard_Illinois_10OS3786_2010 | H10N7 | 11/27/10 | CY132656 | North American |
| A_mallard_Illinois_10OS4078_2010 | H10N7 | 11/28/10 | CY133496 | North American |
| A_mallard_Illinois_10OS4111_2010 | H10N7 | 11/28/10 | CY133504 | North American |
| A_mallard_Illinois_10OS4130_2010 | H11N2 | 11/29/10 | CY133512 | North American |
| A_mallard_Illinois_10OS4179_2010 | H11N2 | 11/29/10 | CY133520 | North American |
| A_mallard_Illinois_10OS4334_2010 | H10N7 | 11/27/10 | CY132892 | North American |
| A_mallard_Illinois_3048_2009 | H11N2 | 10/17/09 | CY096941 | North American |
| A_mallard_Illinois_3051_2009 | H11N3 | 10/17/09 | CY096949 | North American |
| A_mallard_Illinois_3747_2009 | H6N1 | 11/14/09 | CY097236 | North American |
| A_mallard_Illinois_3974_2009 | H5N2 | 10/17/09 | CY097183 | North American |
| A_mallard_Illinois_4124_2009 | H4N8 | 11/29/09 | CY097549 | North American |
| A_mallard_Illinois_4162_2009 | H11N2 | 11/30/09 | CY097565 | North American |
| A_mallard_Illinois_4179_2009 | H11N9 | 11/30/09 | CY097573 | North American |
| A_mallard_Illinois_4180_2009 | H6N1 | 11/30/09 | CY097581 | North American |
| A_mallard_Interior_Alaska_1_2007 | H4N5 | 9/3/07 | CY039839 | North American |
| A_mallard_Interior_Alaska_1_2007 | H3N8 | 9/6/07 | CY035874 | North American |
| A_mallard_Interior_Alaska_10BM01929R0_2010 | H10N7 | 6/15/10 | CY130371 | North American |
| A_mallard_Interior_Alaska_10BM02111R0_2010 | H12N5 | 6/10/10 | CY130379 | North American |
| A_mallard_Interior_Alaska_10BM02530R0_2010 | H4N6 | 6/22/10 | CY130387 | North American |
| A_mallard_Interior_Alaska_10BM02644R0_2010 | H4N6 | 6/25/10 | CY130444 | North American |
| A_mallard_Interior_Alaska_10BM02980R0_2010 | H9N2 | 7/2/10 | CY130460 | North American |
| A_mallard_Interior_Alaska_10BM03979R0_2010 | H4N6 | 7/12/10 | CY130468 | North American |
| A_mallard_Interior_Alaska_10BM04564R1_2010 | H7N3 | 7/18/10 | CY135598 | North American |
| A_mallard_Interior_Alaska_10BM04618R0_2010 | H4N6 | 7/18/10 | CY135606 | North American |
| A_mallard_Interior_Alaska_10BM04623R0_2010 | H4N6 | 7/18/10 | CY135614 | North American |
| A_mallard_Interior_Alaska_10BM04624R0_2010 | H4N6 | 7/18/10 | CY135622 | North American |
| A_mallard_Interior_Alaska_10BM04626R0_2010 | H4N6 | 7/18/10 | CY135630 | North American |
| A_mallard_Interior_Alaska_10BM04988R0_2010 | H4N6 | 7/21/10 | CY135662 | North American |
| A_mallard_Interior_Alaska_10BM05247R0_2010 | H4N6 | 7/23/10 | CY135694 | North American |
| A_mallard_Interior_Alaska_10BM05249R0_2010 | H4N6 | 7/23/10 | CY135702 | North American |
| A_mallard_Interior_Alaska_10BM05337R0_2010 | H4N6 | 7/24/10 | CY135720 | North American |
| A_mallard_Interior_Alaska_10BM05347R0_2010 | H7N3 | 7/25/10 | CY135728 | North American |
| A_mallard_Interior_Alaska_10BM05797R0_2010 | H3N8 | 7/26/10 | CY135818 | North American |
| A_mallard_Interior_Alaska_10BM05860R0_2010 | H7N3 | 7/26/10 | CY135826 | North American |
| A_mallard_Interior_Alaska_10BM05900R0_2010 | H3N8 | 7/26/10 | CY135834 | North American |
| A_mallard_Interior_Alaska_10BM05968R0_2010 | H3N8 | 7/27/10 | CY135842 | North American |
| A_mallard_Interior_Alaska_10BM05970R0_2010 | H3N8 | 7/27/10 | CY135850 | North American |
| A_mallard_Interior_Alaska_10BM06448R0_2010 | H4N6 | 7/30/10 | CY135874 | North American |
| A_mallard_Interior_Alaska_10BM06450R0_2010 | H4N6 | 7/30/10 | CY135882 | North American |
| A_mallard_Interior_Alaska_10BM06452R0_2010 | H4N6 | 7/30/10 | CY135890 | North American |
| A_mallard_Interior_Alaska_10BM06454R0_2010 | H4N6 | 7/30/10 | CY135898 | North American |
| A_mallard_Interior_Alaska_10BM06455R0_2010 | H4N6 | 7/30/10 | CY135906 | North American |
| A_mallard_Interior_Alaska_10BM06456R0_2010 | H4N6 | 7/30/10 | CY135914 | North American |
| A_mallard_Interior_Alaska_10BM06459R0_2010 | H4N6 | 7/30/10 | CY135922 | North American |
| A_mallard_Interior_Alaska_10BM06460R0_2010 | H4N6 | 7/30/10 | CY135930 | North American |
| A_mallard_Interior_Alaska_10BM06461R0_2010 | H4N6 | 7/30/10 | CY135938 | North American |
| A_mallard_Interior_Alaska_10BM06828R0_2010 | H3N8 | 7/31/10 | CY135954 | North American |
| A_mallard_Interior_Alaska_10BM06835R0_2010 | H3N8 | 7/31/10 | CY135962 | North American |
| A_mallard_Interior_Alaska_10BM06838R0_2010 | H7N3 | 7/31/10 | CY135970 | North American |
| A_mallard_Interior_Alaska_10BM06905R0_2010 | H7N3 | 7/31/10 | CY136002 | North American |
| A_mallard_Interior_Alaska_10BM06909R0_2010 | H7N3 | 7/31/10 | CY136010 | North American |
| A_mallard_Interior_Alaska_10BM06911R0_2010 | H7N3 | 7/31/10 | CY136018 | North American |
| A_mallard_Interior_Alaska_10BM07777R0_2010 | H4N6 | 8/3/10 | CY143515 | North American |
| A_mallard_Interior_Alaska_10BM07971R0_2010 | H4N6 | 8/3/10 | CY143539 | North American |
| A_mallard_Interior_Alaska_10BM08622R0_2010 | H4N6 | 8/5/10 | CY143659 | North American |
| A_mallard_Interior_Alaska_10BM08828R0_2010 | H4N6 | 8/5/10 | CY143667 | North American |
| A_mallard_Interior_Alaska_10BM09158R0_2010 | H3N8 | 8/6/10 | CY143699 | North American |
| A_mallard_Interior_Alaska_10BM09159R0_2010 | H3N8 | 8/6/10 | CY143707 | North American |
| A_mallard_Interior_Alaska_10BM09162R0_2010 | H3N8 | 8/6/10 | CY143715 | North American |
| A_mallard_Interior_Alaska_10BM09164R0_2010 | H3N8 | 8/6/10 | CY143723 | North American |
| A_mallard_Interior_Alaska_10BM09561R0_2010 | H3N8 | 8/7/10 | CY143731 | North American |
| A_mallard_Interior_Alaska_10BM09562R0_2010 | H4N6 | 8/7/10 | CY143739 | North American |
| A_mallard_Interior_Alaska_10BM09570R0_2010 | H4N6 | 8/7/10 | CY143747 | North American |
| A_mallard_Interior_Alaska_10BM10000R0_2010 | H3N8 | 8/8/10 | CY143811 | North American |
| A_mallard_Interior_Alaska_10BM10173R0_2010 | H4N6 | 8/8/10 | CY143819 | North American |
| A_mallard_Interior_Alaska_10BM10176R0_2010 | H4N6 | 8/8/10 | CY143827 | North American |
| A_mallard_Interior_Alaska_10BM10829R0_2010 | H7N3 | 8/10/10 | CY143860 | North American |
| A_mallard_Interior_Alaska_10BM10864R0_2010 | H4N6 | 8/10/10 | CY143868 | North American |
| A_mallard_Interior_Alaska_10BM11311R0_2010 | H3N8 | 8/12/10 | CY143884 | North American |
| A_mallard_Interior_Alaska_10BM11414R0_2010 | H3N8 | 8/12/10 | CY143892 | North American |
| A_mallard_Interior_Alaska_10BM11415R0_2010 | H3N8 | 8/12/10 | CY143900 | North American |
| A_mallard_Interior_Alaska_10BM11416R0_2010 | H3N8 | 8/12/10 | CY143908 | North American |
| A_mallard_Interior_Alaska_10BM11418R0_2010 | H3N8 | 8/12/10 | CY143916 | North American |
| A_mallard_Interior_Alaska_10BM11419R0_2010 | H3N8 | 8/12/10 | CY143924 | North American |
| A_mallard_Interior_Alaska_10BM11420R0_2010 | H3N8 | 8/12/10 | CY143932 | North American |
| A_mallard_Interior_Alaska_10BM11421R0_2010 | H3N8 | 8/12/10 | CY143940 | North American |
| A_mallard_Interior_Alaska_10BM11497R0_2010 | H3N6 | 8/12/10 | CY143958 | North American |
| A_mallard_Interior_Alaska_10BM12049R0_2010 | H3N8 | 8/13/10 | CY144102 | North American |
| A_mallard_Interior_Alaska_10BM12172R0_2010 | H3N8 | 8/13/10 | CY143990 | North American |
| A_mallard_Interior_Alaska_10BM12177R0_2010 | H3N8 | 8/13/10 | CY143998 | North American |
| A_mallard_Interior_Alaska_10BM12178R0_2010 | H3N8 | 8/13/10 | CY144006 | North American |
| A_mallard_Interior_Alaska_10BM12179R0_2010 | H3N8 | 8/13/10 | CY144014 | North American |
| A_mallard_Interior_Alaska_10BM12308R0_2010 | H4N6 | 8/14/10 | CY144022 | North American |
| A_mallard_Interior_Alaska_10BM12311R0_2010 | H3N8 | 8/14/10 | CY144030 | North American |
| A_mallard_Interior_Alaska_10BM12350R0_2010 | H3N8 | 8/14/10 | CY144038 | North American |
| A_mallard_Interior_Alaska_10BM12351R0_2010 | H3N8 | 8/14/10 | CY144046 | North American |
| A_mallard_Interior_Alaska_10BM12352R0_2010 | H3N8 | 8/14/10 | CY144054 | North American |
| A_mallard_Interior_Alaska_10BM12362R0_2010 | H3N8 | 8/14/10 | CY141514 | North American |
| A_mallard_Interior_Alaska_10BM12363R0_2010 | H3N8 | 8/14/10 | CY144062 | North American |
| A_mallard_Interior_Alaska_10BM12370R0_2010 | H3N8 | 8/14/10 | CY144070 | North American |
| A_mallard_Interior_Alaska_10BM12374R0_2010 | H3N8 | 8/14/10 | CY144078 | North American |
| A_mallard_Interior_Alaska_10BM12383R0_2010 | H3N8 | 8/14/10 | CY144086 | North American |
| A_mallard_Interior_Alaska_10BM12582R0_2010 | H3N8 | 8/14/10 | CY141522 | North American |
| A_mallard_Interior_Alaska_10CH00021R0_2010 | H4N6 | 9/4/10 | CY143763 | North American |
| A_mallard_Interior_Alaska_10CH00035R0_2010 | H3N8 | 9/5/10 | CY143771 | North American |
| A_mallard_Interior_Alaska_2_2007 | H3N8 | 9/6/07 | CY035796 | North American |
| A_mallard_Interior_Alaska_2_2007 | H4N6 | 9/10/07 | CY039807 | North American |
| A_mallard_Interior_Alaska_3_2007 | H3N8 | 9/7/07 | CY038359 | North American |
| A_mallard_Interior_Alaska_3_2007 | H4N6 | 9/16/07 | CY039863 | North American |
| A_mallard_Interior_Alaska_4_2007 | H3N8 | 12/2/07 | CY038375 | North American |
| A_mallard_Interior_Alaska_5_2007 | H3N8 | 9/10/07 | CY039815 | North American |
| A_mallard_Interior_Alaska_6_2007 | H3N8 | 8/17/07 | CY039752 | North American |
| A_mallard_Interior_Alaska_6MP0038BR2_2006 | H2N3 | 8/11/06 | CY078698 | North American |
| A_mallard_Interior_Alaska_6MP0050R1_2006 | H3N8 | 8/11/06 | CY078570 | North American |
| A_mallard_Interior_Alaska_6MP0124_2006 | H3N8 | 8/10/06 | CY078882 | North American |
| A_mallard_Interior_Alaska_6MP0155_2006 | H3N8 | 8/11/06 | CY078434 | North American |
| A_mallard_Interior_Alaska_6MP0160AR1_2006 | H3N8 | 8/12/06 | CY078682 | North American |
| A_mallard_Interior_Alaska_6MP0163AR2_2006 | H3N8 | 8/12/06 | CY078706 | North American |
| A_mallard_Interior_Alaska_6MP0272_2006 | H3N8 | 8/15/06 | CY078578 | North American |
| A_mallard_Interior_Alaska_6MP0745_2006 | H4N6 | 8/8/06 | CY078450 | North American |
| A_mallard_Interior_Alaska_6MP0758_2006 | H10N8 | 8/8/06 | CY078466 | North American |
| A_mallard_Interior_Alaska_6MP0878_2006 | H3N8 | 8/16/06 | CY078594 | North American |
| A_mallard_Interior_Alaska_6MP0891_2006 | H4N6 | 8/16/06 | CY078602 | North American |
| A_mallard_Interior_Alaska_6MP0915_2006 | H3N8 | 8/17/06 | CY078490 | North American |
| A_mallard_Interior_Alaska_6MP0935R1_2006 | H3N8 | 8/17/06 | CY078650 | North American |
| A_mallard_Interior_Alaska_6MP0951_2006 | H3N8 | 8/18/06 | CY078610 | North American |
| A_mallard_Interior_Alaska_6MP0952_2006 | H3N6 | 8/18/06 | CY078618 | North American |
| A_mallard_Interior_Alaska_6MP0956R1_2006 | H4N6 | 8/18/06 | CY078666 | North American |
| A_mallard_Interior_Alaska_6MP0972R1_2006 | H3N8 | 8/17/06 | CY078674 | North American |
| A_mallard_Interior_Alaska_6MP0975_2006 | H3N8 | 8/18/06 | CY078498 | North American |
| A_mallard_Interior_Alaska_6MP0983R1_2006 | H3N8 | 8/18/06 | CY079747 | North American |
| A_mallard_Interior_Alaska_6MP0984_2006 | H7N3 | 8/19/06 | CY078890 | North American |
| A_mallard_Interior_Alaska_6MP0991_2006 | H3N2 | 8/19/06 | CY078506 | North American |
| A_mallard_Interior_Alaska_6MP0992_2006 | H3N2 | 8/19/06 | CY078514 | North American |
| A_mallard_Interior_Alaska_6MP1107R1_2006 | H3N8 | 9/8/06 | CY079763 | North American |
| A_mallard_Interior_Alaska_6MP1142_2006 | H3N6 | 9/22/06 | CY078634 | North American |
| A_mallard_Interior_Alaska_7_2007 | H3N8 | 9/5/07 | CY039847 | North American |
| A_mallard_Interior_Alaska_7MP0167_2007 | H12N5 | 8/13/07 | CY077205 | North American |
| A_mallard_Interior_Alaska_7MP0172_2007 | H1N1 | 8/13/07 | CY078938 | North American |
| A_mallard_Interior_Alaska_7MP0510_2007 | H3N6 | 9/7/07 | CY077237 | North American |
| A_mallard_Interior_Alaska_7MP0512_2007 | H4N6 | 9/7/07 | CY077269 | North American |
| A_mallard_Interior_Alaska_7MP0709_2007 | H3N8 | 8/9/07 | CY045438 | North American |
| A_mallard_Interior_Alaska_7MP0747_2007 | H1N1 | 8/10/07 | CY078962 | North American |
| A_mallard_Interior_Alaska_7MP1050R1_2007 | H4N6 | 9/10/07 | CY045430 | North American |
| A_mallard_Interior_Alaska_7MP1056_2007 | H3N8 | 9/10/07 | CY077293 | North American |
| A_mallard_Interior_Alaska_7MP1094_2007 | H3N8 | 9/13/07 | CY077403 | North American |
| A_mallard_Interior_Alaska_7MP1700_2007 | H3N8 | 9/5/07 | CY077379 | North American |
| A_mallard_Interior_Alaska_7MP1757_2007 | H3N8 | 9/3/07 | CY047042 | North American |
| A_mallard_Interior_Alaska_7MP1771R1_2007 | H4N6 | 9/5/07 | CY078898 | North American |
| A_mallard_Interior_Alaska_7MP1835_2007 | H3N8 | 9/5/07 | CY079018 | North American |
| A_mallard_Interior_Alaska_7MP2230_2007 | H1N6 | 9/16/07 | CY077261 | North American |
| A_mallard_Interior_Alaska_8BM2102_2008 | H1N1 | 8/9/08 | CY080122 | North American |
| A_mallard_Interior_Alaska_8BM2312_2008 | H3N8 | 8/11/08 | CY080168 | North American |
| A_mallard_Interior_Alaska_8BM2967_2008 | H12N5 | 8/16/08 | CY079074 | North American |
| A_mallard_Interior_Alaska_8BM3061_2008 | H8N4 | 8/18/08 | CY079106 | North American |
| A_mallard_Interior_Alaska_8BM3584R1_2008 | H8N4 | 9/14/08 | CY079684 | North American |
| A_mallard_Interior_Alaska_8BM3586_2008 | H3N8 | 9/14/08 | CY079130 | North American |
| A_mallard_Interior_Alaska_8BM3614_2008 | H3N8 | 9/16/08 | CY079138 | North American |
| A_mallard_Interior_Alaska_8BM3627R1_2008 | H4N6 | 9/16/08 | CY079202 | North American |
| A_mallard_Interior_Alaska_8MP0457R1_2008 | H8N4 | 8/10/08 | CY079970 | North American |
| A_mallard_Interior_Alaska_8MP0547_2008 | H8N4 | 8/11/08 | CY079379 | North American |
| A_mallard_Interior_Alaska_8MP0792R1_2008 | H12N5 | 8/19/08 | CY079194 | North American |
| A_mallard_Interior_Alaska_9BM10047R0_2009 | H3N8 | 8/15/09 | CY142729 | North American |
| A_mallard_Interior_Alaska_9BM10051R0_2009 | H3N8 | 8/15/09 | CY142737 | North American |
| A_mallard_Interior_Alaska_9BM10055R0_2009 | H3N8 | 8/15/09 | CY142745 | North American |
| A_mallard_Interior_Alaska_9BM10095R0_2009 | H4N6 | 8/15/09 | CY142753 | North American |
| A_mallard_Interior_Alaska_9BM10111R0_2009 | H3N8 | 8/15/09 | CY142761 | North American |
| A_mallard_Interior_Alaska_9BM10375R0_2009 | H4N6 | 8/17/09 | CY143403 | North American |
| A_mallard_Interior_Alaska_9BM10563R2_2009 | H4N6 | 8/16/09 | CY141456 | North American |
| A_mallard_Interior_Alaska_9BM10717R0_2009 | H3N8 | 8/17/09 | CY143049 | North American |
| A_mallard_Interior_Alaska_9BM10851R0_2009 | H3N8 | 8/17/09 | CY143089 | North American |
| A_mallard_Interior_Alaska_9BM11015R0_2009 | H4N6 | 8/17/09 | CY142793 | North American |
| A_mallard_Interior_Alaska_9BM11381R0_2009 | H3N8 | 8/18/09 | CY142809 | North American |
| A_mallard_Interior_Alaska_9BM11542R0_2009 | H3N8 | 8/19/09 | CY142825 | North American |
| A_mallard_Interior_Alaska_9BM11867R0_2009 | H3N8 | 8/19/09 | CY142865 | North American |
| A_mallard_Interior_Alaska_9BM11965R0_2009 | H3N8 | 8/20/09 | CY142889 | North American |
| A_mallard_Interior_Alaska_9BM12198R0_2009 | H4N6 | 8/21/09 | CY143129 | North American |
| A_mallard_Interior_Alaska_9BM12204R0_2009 | H4N6 | 8/21/09 | CY143137 | North American |
| A_mallard_Interior_Alaska_9BM12285R0_2009 | H3N8 | 9/1/09 | CY143153 | North American |
| A_mallard_Interior_Alaska_9BM12326R0_2009 | H3N8 | 9/2/09 | CY143185 | North American |
| A_mallard_Interior_Alaska_9BM12388R0_2009 | H3N8 | 9/2/09 | CY143209 | North American |
| A_mallard_Interior_Alaska_9BM12390R0_2009 | H3N8 | 9/2/09 | CY143217 | North American |
| A_mallard_Interior_Alaska_9BM12523R0_2009 | H3N8 | 9/3/09 | CY143241 | North American |
| A_mallard_Interior_Alaska_9BM12567R0_2009 | H3N8 | 9/4/09 | CY143313 | North American |
| A_mallard_Interior_Alaska_9BM12627R0_2009 | H3N8 | 9/4/09 | CY143321 | North American |
| A_mallard_Interior_Alaska_9BM1585R1_2009 | H12N5 | 6/24/09 | CY078874 | North American |
| A_mallard_Interior_Alaska_9BM1799_2009 | H12N5 | 7/1/09 | CY079785 | North American |
| A_mallard_Interior_Alaska_9BM1807_2009 | H4N6 | 7/2/09 | CY078746 | North American |
| A_mallard_Interior_Alaska_9BM1809_2009 | H4N6 | 7/2/09 | CY079799 | North American |
| A_mallard_Interior_Alaska_9BM1812_2009 | H4N6 | 7/2/09 | CY078722 | North American |
| A_mallard_Interior_Alaska_9BM1852_2009 | H10N5 | 7/6/09 | CY078730 | North American |
| A_mallard_Interior_Alaska_9BM1856_2009 | H4N6 | 7/2/09 | CY078738 | North American |
| A_mallard_Interior_Alaska_9BM1864_2009 | H4N6 | 7/1/09 | CY079589 | North American |
| A_mallard_Interior_Alaska_9BM1869_2009 | H4N6 | 7/1/09 | CY078754 | North American |
| A_mallard_Interior_Alaska_9BM1947_2009 | H4N6 | 7/4/09 | CY078762 | North American |
| A_mallard_Interior_Alaska_9BM1949_2009 | H4N6 | 7/4/09 | CY078770 | North American |
| A_mallard_Interior_Alaska_9BM1957_2009 | H4N6 | 7/4/09 | CY079597 | North American |
| A_mallard_Interior_Alaska_9BM1958_2009 | H4N6 | 7/4/09 | CY078778 | North American |
| A_mallard_Interior_Alaska_9BM1959_2009 | H4N6 | 7/4/09 | CY079822 | North American |
| A_mallard_Interior_Alaska_9BM1962_2009 | H4N6 | 7/4/09 | CY078786 | North American |
| A_mallard_Interior_Alaska_9BM1964_2009 | H4N6 | 7/6/09 | CY079605 | North American |
| A_mallard_Interior_Alaska_9BM1965_2009 | H4N6 | 7/6/09 | CY078794 | North American |
| A_mallard_Interior_Alaska_9BM1967_2009 | H4N6 | 7/6/09 | CY078802 | North American |
| A_mallard_Interior_Alaska_9BM1968_2009 | H4N6 | 7/6/09 | CY079830 | North American |
| A_mallard_Interior_Alaska_9BM1969_2009 | H4N6 | 7/6/09 | CY079838 | North American |
| A_mallard_Interior_Alaska_9BM1974_2009 | H4N6 | 7/6/09 | CY078810 | North American |
| A_mallard_Interior_Alaska_9BM1975_2009 | H4N6 | 7/6/09 | CY078818 | North American |
| A_mallard_Interior_Alaska_9BM2057_2009 | H4N6 | 7/8/09 | CY078826 | North American |
| A_mallard_Interior_Alaska_9BM2073_2009 | H4N6 | 7/9/09 | CY079854 | North American |
| A_mallard_Interior_Alaska_9BM2077_2009 | H4N6 | 7/9/09 | CY078834 | North American |
| A_mallard_Interior_Alaska_9BM2164_2009 | H4N6 | 7/10/09 | CY079869 | North American |
| A_mallard_Interior_Alaska_9BM2168_2009 | H4N6 | 7/10/09 | CY078842 | North American |
| A_mallard_Interior_Alaska_9BM2170_2009 | H4N6 | 7/10/09 | CY078850 | North American |
| A_mallard_Interior_Alaska_9BM2239_2009 | H4N6 | 7/11/09 | CY079877 | North American |
| A_mallard_Interior_Alaska_9BM2241R1_2009 | H4N6 | 7/11/09 | CY130363 | North American |
| A_mallard_Interior_Alaska_9BM2243_2009 | H4N6 | 7/11/09 | CY079885 | North American |
| A_mallard_Interior_Alaska_9BM2250R0_2009 | H4N6 | 7/11/09 | CY130355 | North American |
| A_mallard_Interior_Alaska_9BM2252_2009 | H4N6 | 7/11/09 | CY079893 | North American |
| A_mallard_Interior_Alaska_9BM2254_2009 | H4N6 | 7/11/09 | CY078858 | North American |
| A_mallard_Interior_Alaska_9BM2256_2009 | H4N6 | 7/11/09 | CY078866 | North American |
| A_mallard_Interior_Alaska_9BM2259_2009 | H4N6 | 7/11/09 | CY079613 | North American |
| A_mallard_Interior_Alaska_9BM2351R0_2009 | H8N4 | 7/12/09 | CY130239 | North American |
| A_mallard_Interior_Alaska_9BM2388R0_2009 | H8N4 | 7/13/09 | CY130208 | North American |
| A_mallard_Interior_Alaska_9BM2393R0_2009 | H4N6 | 7/13/09 | CY130247 | North American |
| A_mallard_Interior_Alaska_9BM2395R0_2009 | H4N6 | 7/13/09 | CY130255 | North American |
| A_mallard_Interior_Alaska_9BM2398R0_2009 | H4N6 | 7/13/09 | CY130216 | North American |
| A_mallard_Interior_Alaska_9BM2535R0_2009 | H4N6 | 7/12/09 | CY130263 | North American |
| A_mallard_Interior_Alaska_9BM2536R0_2009 | H4N6 | 7/13/09 | CY130271 | North American |
| A_mallard_Interior_Alaska_9BM2606R0_2009 | H4N6 | 7/14/09 | CY130279 | North American |
| A_mallard_Interior_Alaska_9BM2705R0_2009 | H8N4 | 7/15/09 | CY130287 | North American |
| A_mallard_Interior_Alaska_9BM2750R0_2009 | H10N7 | 7/15/09 | CY130295 | North American |
| A_mallard_Interior_Alaska_9BM2751R0_2009 | H10N7 | 7/15/09 | CY130303 | North American |
| A_mallard_Interior_Alaska_9BM2783R0_2009 | H10N7 | 7/15/09 | CY130311 | North American |
| A_mallard_Interior_Alaska_9BM3016R0_2009 | H10N7 | 7/17/09 | CY135342 | North American |
| A_mallard_Interior_Alaska_9BM3075R2_2009 | H10N7 | 7/18/09 | CY135350 | North American |
| A_mallard_Interior_Alaska_9BM3076R0_2009 | H10N7 | 7/18/09 | CY135358 | North American |
| A_mallard_Interior_Alaska_9BM3078R0_2009 | H10N7 | 7/18/09 | CY135366 | North American |
| A_mallard_Interior_Alaska_9BM3205R0_2009 | H10N7 | 7/20/09 | CY135382 | North American |
| A_mallard_Interior_Alaska_9BM3355R0_2009 | H10N7 | 7/21/09 | CY135390 | North American |
| A_mallard_Interior_Alaska_9BM3357R0_2009 | H10N7 | 7/21/09 | CY135398 | North American |
| A_mallard_Interior_Alaska_9BM3361R0_2009 | H10N7 | 7/21/09 | CY135486 | North American |
| A_mallard_Interior_Alaska_9BM3379R0_2009 | H10N7 | 7/22/09 | CY135494 | North American |
| A_mallard_Interior_Alaska_9BM6886R0_2009 | H4N6 | 8/10/09 | CY142649 | North American |
| A_mallard_Interior_Alaska_9BM8471R0_2009 | H4N6 | 8/10/09 | CY142993 | North American |
| A_mallard_Interior_Alaska_9BM8829R0_2009 | H4N6 | 8/11/09 | CY143025 | North American |
| A_mallard_Interior_Alaska_9BM9049R0_2009 | H4N6 | 8/11/09 | CY143041 | North American |
| A_mallard_Interior_Alaska_9BM9683R2_2009 | H4N6 | 8/14/09 | CY143265 | North American |
| A_mallard_Interior_Alaska_9BM9755R0_2009 | H3N1 | 8/14/09 | CY143273 | North American |
| A_mallard_Interior_Alaska_9BM9983R0_2009 | H4N6 | 8/15/09 | CY143289 | North American |
| A_mallard_Interior_Alaska_9BM9984R0_2009 | H4N6 | 8/15/09 | CY143459 | North American |
| A_mallard_Interior_Alaska_9BM9985R0_2009 | H4N6 | 8/15/09 | CY143297 | North American |
| A_mallard_Iowa_10OS2420_2010 | H4N6 | 9/18/10 | CY133584 | North American |
| A_mallard_Iowa_10OS2426_2010 | H3N8 | 9/18/10 | CY133592 | North American |
| A_mallard_Iowa_10OS2492_2010 | H3N6 | 9/18/10 | CY133600 | North American |
| A_mallard_Iowa_10OS2685_2010 | H4N2 | 10/16/10 | CY133439 | North American |
| A_mallard_Iowa_10OS2692_2010 | H4N2 | 10/16/10 | CY133447 | North American |
| A_mallard_Iowa_10OS2721_2010 | H2N2 | 10/16/10 | CY133455 | North American |
| A_mallard_Iowa_10OS2752_2010 | H11N9 | 10/17/10 | CY133471 | North American |
| A_mallard_Iowa_3193_2009 | H11N9 | 11/7/09 | CY097589 | North American |
| A_mallard_Manitoba_23912_2007 | H4N7 | 8/24/07 | CY047695 | North American |
| A_mallard_Michigan_10OS1491_2010 | H4N6 | 8/20/10 | CY132124 | North American |
| A_mallard_Michigan_10OS1497_2010 | H3N2 | 8/20/10 | CY132132 | North American |
| A_mallard_Minnesota_AI08-3437_2008 | H2N3 | 8/2/08 | CY141167 | North American |
| A_mallard_Minnesota_AI08-3881_2008 | H2N2 | 9/3/08 | CY141175 | North American |
| A_mallard_Minnesota_AI09-1666_2009 | H10N3 | 7/27/09 | CY146439 | North American |
| A_mallard_Minnesota_AI09-1669_2009 | H10N3 | 7/27/09 | CY146447 | North American |
| A_mallard_Minnesota_AI09-1678_2009 | H1N1 | 7/27/09 | CY140646 | North American |
| A_mallard_Minnesota_AI09-1684_2009 | H1N1 | 7/27/09 | CY140654 | North American |
| A_mallard_Minnesota_AI09-1829_2009 | H3N8 | 7/28/09 | CY140662 | North American |
| A_mallard_Minnesota_AI09-1833_2009 | H8N4 | 7/28/09 | CY140670 | North American |
| A_mallard_Minnesota_AI09-1851_2009 | H10N5 | 7/28/09 | CY146455 | North American |
| A_mallard_Minnesota_AI09-1854_2009 | H8N4 | 7/28/09 | CY140678 | North American |
| A_mallard_Minnesota_AI09-1864_2009 | H10N5 | 7/28/09 | CY146463 | North American |
| A_mallard_Minnesota_AI09-1867_2009 | H8N4 | 7/28/09 | CY140686 | North American |
| A_mallard_Minnesota_AI09-1870_2009 | H3N3 | 7/28/09 | CY140694 | North American |
| A_mallard_Minnesota_AI09-2434_2009 | H3N8 | 8/5/09 | CY140710 | North American |
| A_mallard_Minnesota_AI09-2509_2009 | H6N1 | 9/6/09 | CY140718 | North American |
| A_mallard_Minnesota_AI09-2518_2009 | H2N3 | 9/6/09 | CY140726 | North American |
| A_mallard_Minnesota_AI09-2519_2009 | H3N6 | 9/6/09 | CY140734 | North American |
| A_mallard_Minnesota_AI09-2571_2009 | H3N8 | 9/7/09 | CY140742 | North American |
| A_mallard_Minnesota_AI09-3066_2009 | H12N5 | 9/7/09 | CY140787 | North American |
| A_mallard_Minnesota_AI09-3100_2009 | H6N3 | 9/8/09 | CY140795 | North American |
| A_mallard_Minnesota_AI09-3228_2009 | H3N1 | 9/8/09 | CY140811 | North American |
| A_mallard_Minnesota_AI09-3302_2009 | H11N9 | 9/9/09 | CY140827 | North American |
| A_mallard_Minnesota_AI09-3353_2009 | H2N9 | 9/9/09 | CY140835 | North American |
| A_mallard_Minnesota_AI09-3821_2009 | H4N6 | 9/13/09 | CY140902 | North American |
| A_mallard_Minnesota_AI09-4022_2009 | H3N2 | 9/6/09 | CY140918 | North American |
| A_mallard_Minnesota_AI10-2217_2010 | H12N5 | 8/4/10 | CY141104 | North American |
| A_mallard_Minnesota_AI10-2762_2010 | H11N9 | 9/1/10 | CY141150 | North American |
| A_mallard_Minnesota_Sg-00045_2007 | H4N6 | 8/3/07 | CY064000 | North American |
| A_mallard_Minnesota_Sg-00047_2007 | H3N8 | 8/3/07 | CY064016 | North American |
| A_mallard_Minnesota_Sg-00048_2007 | H3N8 | 8/3/07 | CY064024 | North American |
| A_mallard_Minnesota_Sg-00050_2007 | H4N6 | 8/3/07 | CY064040 | North American |
| A_mallard_Minnesota_Sg-00052_2007 | H4N6 | 8/3/07 | CY064056 | North American |
| A_mallard_Minnesota_Sg-00053_2007 | H4N6 | 8/3/07 | CY064064 | North American |
| A_mallard_Minnesota_Sg-00055_2007 | H12N5 | 8/2/07 | CY064072 | North American |
| A_mallard_Minnesota_Sg-00056_2007 | H10N7 | 8/3/07 | CY064080 | North American |
| A_mallard_Minnesota_Sg-00057_2007 | H10N7 | 8/3/07 | CY064088 | North American |
| A_mallard_Minnesota_Sg-00058_2007 | H4N6 | 8/3/07 | CY064096 | North American |
| A_mallard_Minnesota_Sg-00059_2007 | H3N8 | 8/3/07 | CY064104 | North American |
| A_mallard_Minnesota_Sg-00061_2007 | H3N8 | 8/3/07 | CY064120 | North American |
| A_mallard_Minnesota_Sg-00062_2007 | H8N4 | 8/3/07 | CY064128 | North American |
| A_mallard_Minnesota_Sg-00063_2007 | H4N6 | 8/3/07 | CY064136 | North American |
| A_mallard_Minnesota_Sg-00064_2007 | H3N8 | 8/3/07 | CY064144 | North American |
| A_mallard_Minnesota_Sg-00065_2007 | H10N7 | 8/3/07 | CY064152 | North American |
| A_mallard_Minnesota_Sg-00106_2007 | H6N2 | 9/16/07 | CY078280 | North American |
| A_mallard_Minnesota_Sg-00166_2007 | H6N2 | 9/22/07 | CY078296 | North American |
| A_mallard_Minnesota_Sg-00195_2007 | H10N3 | 9/22/07 | CY080294 | North American |
| A_mallard_Minnesota_Sg-00200_2007 | H3N8 | 9/22/07 | CY078169 | North American |
| A_mallard_Minnesota_Sg-00220_2007 | H6N1 | 9/14/07 | CY078344 | North American |
| A_mallard_Minnesota_Sg-00570_2008 | H8N4 | 8/3/08 | CY038258 | North American |
| A_mallard_Minnesota_Sg-00570_2008 | H8N4 | 8/3/08 | CY139992 | North American |
| A_mallard_Minnesota_Sg-00589_2008 | H3N8 | 7/31/08 | CY140020 | North American |
| A_mallard_Minnesota_Sg-00590_2008 | H3N8 | 7/31/08 | CY140028 | North American |
| A_mallard_Minnesota_Sg-00591_2008 | H3N8 | 7/31/08 | CY140036 | North American |
| A_mallard_Minnesota_Sg-00605_2008 | H3N8 | 7/31/08 | CY140044 | North American |
| A_mallard_Minnesota_Sg-00616_2008 | H4N8 | 7/29/08 | CY140276 | North American |
| A_mallard_Minnesota_Sg-00620_2008 | H1N1 | 7/29/08 | CY140052 | North American |
| A_mallard_Minnesota_Sg-00630_2008 | H10N7 | 7/29/08 | CY042668 | North American |
| A_mallard_Minnesota_Sg-00630_2008 | H10N7 | 7/29/08 | CY145734 | North American |
| A_mallard_Minnesota_Sg-00672_2008 | H3N8 | 8/3/08 | CY042832 | North American |
| A_mallard_Minnesota_Sg-00672_2008 | H3N8 | 8/3/08 | CY140309 | North American |
| A_mallard_Minnesota_Sg-00674_2008 | H3N8 | 8/3/08 | CY140317 | North American |
| A_mallard_Minnesota_Sg-00682_2008 | H8N4 | 8/3/08 | CY042871 | North American |
| A_mallard_Minnesota_Sg-00682_2008 | H8N4 | 8/3/08 | CY140325 | North American |
| A_mallard_Minnesota_Sg-00684_2008 | H8N4 | 8/3/08 | CY042879 | North American |
| A_mallard_Minnesota_Sg-00684_2008 | H8N4 | 8/3/08 | CY140095 | North American |
| A_mallard_Minnesota_Sg-00686_2008 | H8N4 | 8/3/08 | CY140103 | North American |
| A_mallard_Minnesota_Sg-00692_2008 | H2N3 | 8/1/08 | CY042911 | North American |
| A_mallard_Minnesota_Sg-00692_2008 | H2N3 | 8/1/08 | CY140333 | North American |
| A_mallard_Minnesota_Sg-00697_2008 | H4N6 | 8/1/08 | CY042931 | North American |
| A_mallard_Minnesota_Sg-00697_2008 | H4N6 | 8/1/08 | CY140341 | North American |
| A_mallard_Minnesota_Sg-00701_2008 | H8N4 | 8/3/08 | CY042943 | North American |
| A_mallard_Minnesota_Sg-00701_2008 | H8N4 | 8/3/08 | CY140349 | North American |
| A_mallard_Minnesota_Sg-00767_2008 | H4N8 | 8/27/08 | CY140179 | North American |
| A_mallard_Minnesota_Sg-00768_2008 | H6N2 | 9/1/08 | CY140187 | North American |
| A_mallard_Minnesota_Sg-00782_2008 | H2N3 | 8/2/08 | CY140195 | North American |
| A_mallard_Minnesota_Sg-00804_2008 | H3N8 | 9/1/08 | CY140219 | North American |
| A_mallard_Minnesota_Sg-00831_2008 | H3N8 | 9/4/08 | CY140399 | North American |
| A_mallard_Minnesota_Sg-00837_2008 | H6N1 | 9/4/08 | CY140417 | North American |
| A_mallard_Minnesota_Sg-00868_2008 | H3N8 | 9/3/08 | CY140456 | North American |
| A_mallard_Minnesota_Sg-00927_2008 | H6N1 | 9/17/08 | CY140227 | North American |
| A_mallard_Minnesota_Sg-00929_2008 | H3N8 | 9/16/08 | CY140244 | North American |
| A_mallard_Minnesota_Sg-00930_2008 | H11N9 | 9/16/08 | CY140474 | North American |
| A_mallard_Minnesota_Sg-00931_2008 | H4N6 | 9/17/08 | CY140482 | North American |
| A_mallard_Minnesota_Sg-00932_2008 | H6N1 | 9/16/08 | CY140252 | North American |
| A_mallard_Minnesota_Sg-00949_2008 | H3N2 | 9/3/08 | CY140490 | North American |
| A_mallard_Minnesota_Sg-01018_2008 | H6N2 | 9/9/08 | CY140526 | North American |
| A_mallard_Minnesota_Sg-01030_2008 | H10N7 | 9/12/08 | CY145857 | North American |
| A_mallard_Minnesota_Sg-01034_2008 | H11N2 | 10/14/08 | CY140566 | North American |
| A_mallard_Minnesota_Sg-01035_2008 | H3N8 | 9/14/08 | CY140574 | North American |
| A_mallard_Minnesota_Sg-01039_2008 | H4N8 | 9/14/08 | CY140582 | North American |
| A_mallard_Minnesota_Sg-01040_2008 | H3N2 | 9/14/08 | CY140590 | North American |
| A_mallard_Minnesota_Sg-01042_2008 | H6N8 | 9/14/08 | CY140598 | North American |
| A_mallard_Minnesota_Sg-01047_2008 | H6N1 | 9/25/08 | CY140606 | North American |
| A_mallard_Minnesota_Sg-0617_2008 | H1N1 | 7/29/08 | CY140284 | North American |
| A_mallard_Mississippi_10OS4494_2010 | H1N1 | 12/10/10 | CY133305 | North American |
| A_mallard_Mississippi_10OS4593_2010 | H1N2 | 12/11/10 | CY133297 | North American |
| A_mallard_Mississippi_329_2010 | H10N7 | 1/16/10 | CY097701 | North American |
| A_mallard_Mississippi_390_2010 | H3N8 | 1/17/10 | CY097733 | North American |
| A_mallard_Mississippi_413_2010 | H1N1 | 1/17/10 | CY097769 | North American |
| A_mallard_Mississippi_442_2010 | H1N1 | 1/19/10 | CY097777 | North American |
| A_mallard_Missouri_10MO0253_2010 | H1N2 | 11/16/10 | CY133808 | North American |
| A_mallard_Missouri_10MO0333_2010 | H11N9 | 12/22/10 | CY133832 | North American |
| A_mallard_Missouri_10MO0391_2010 | H10N7 | 12/22/10 | CY133850 | North American |
| A_mallard_Missouri_10MO053_2010 | H7N4 | 11/16/10 | CY133784 | North American |
| A_mallard_Missouri_10MO0550_2010 | H11N3 | 12/29/10 | CY133876 | North American |
| A_mallard_Missouri_10MO0551_2010 | H7N7 | 12/29/10 | CY132148 | North American |
| A_mallard_Missouri_10MO084_2010 | H5N9 | 11/16/10 | CY133792 | North American |
| A_mallard_Missouri_10OS4670_2010 | H9N2 | 12/12/10 | CY133380 | North American |
| A_mallard_Missouri_129_2009 | H6N2 | 12/7/09 | CY097597 | North American |
| A_mallard_Missouri_132_2009 | H2N3 | 12/7/09 | CY097605 | North American |
| A_mallard_Missouri_220_2009 | H7N3 | 12/7/09 | CY097629 | North American |
| A_mallard_Missouri_350_2009 | H11N9 | 12/6/09 | CY097100 | North American |
| A_mallard_New_Brunswick_00324_2010 | H1N1 | 8/8/10 | CY138663 | North American |
| A_mallard_New_Brunswick_00329_2010 | H1N1 | 8/8/10 | CY138687 | North American |
| A_mallard_New_Brunswick_00337_2010 | H1N1 | 8/8/10 | CY138744 | North American |
| A_mallard_New_Brunswick_00339_2010 | H1N1 | 8/8/10 | CY138752 | North American |
| A_mallard_New_Brunswick_00340_2010 | H1N1 | 8/8/10 | CY138760 | North American |
| A_mallard_New_Brunswick_00341_2010 | H3N8 | 8/8/10 | CY138768 | North American |
| A_mallard_New_Brunswick_00349_2010 | H1N1 | 8/8/10 | CY138792 | North American |
| A_mallard_New_Brunswick_00495_2010 | H3N8 | 8/12/10 | CY139031 | North American |
| A_mallard_New_Brunswick_00593_2010 | H3N7 | 8/15/10 | CY139106 | North American |
| A_mallard_New_Brunswick_00854_2010 | H4N6 | 9/13/10 | CY139326 | North American |
| A_mallard_New_Brunswick_00879_2010 | H4N9 | 9/12/10 | CY139359 | North American |
| A_mallard_New_Brunswick_00979_2010 | H4N8 | 9/16/10 | CY139504 | North American |
| A_mallard_New_Brunswick_02293_2007 | H4N6 | 8/20/07 | CY129357 | North American |
| A_mallard_New_Brunswick_02294_2007 | H4N6 | 8/20/07 | CY128705 | North American |
| A_mallard_New_Brunswick_02311_2007 | H4N6 | 8/20/07 | CY129125 | North American |
| A_mallard_New_Brunswick_02747_2007 | H3N8 | 9/4/07 | CY129309 | North American |
| A_mallard_New_Brunswick_03396_2009 | H3N8 | 8/9/09 | CY129045 | North American |
| A_mallard_New_Brunswick_03397_2009 | H3N8 | 8/9/09 | CY129005 | North American |
| A_mallard_New_Brunswick_03482_2009 | H3N8 | 8/14/09 | CY128563 | North American |
| A_mallard_New_Brunswick_04394_2007 | H10N7 | 9/4/07 | CY129373 | North American |
| A_mallard_New_Brunswick_04400_2007 | H3N8 | 9/4/07 | CY128713 | North American |
| A_mallard_New_Brunswick_04482_2007 | H3N8 | 9/4/07 | CY129236 | North American |
| A_mallard_New_Brunswick_04485_2007 | H3N8 | 9/4/07 | CY129293 | North American |
| A_mallard_New_Jersey_AI09-5121_2009 | H3N8 | 9/29/09 | CY146480 | North American |
| A_mallard_New_Jersey_AI09-5122_2009 | H11N9 | 9/28/09 | CY146488 | North American |
| A_mallard_New_Jersey_AI09-5137_2009 | H4N9 | 9/28/09 | CY146496 | North American |
| A_mallard_New_Jersey_AI09-5361_2009 | H11N9 | 9/28/09 | CY146504 | North American |
| A_mallard_New_Jersey_AI09-5405_2009 | H6N2 | 9/28/09 | CY146512 | North American |
| A_mallard_New_Jersey_AI09-6426_2009 | H4N9 | 9/28/09 | CY146528 | North American |
| A_mallard_New_Jersey_AI09-6434_2009 | H6N2 | 9/28/09 | CY146536 | North American |
| A_mallard_New_Jersey_AI09-6446_2009 | H4N1 | 9/28/09 | CY146544 | North American |
| A_mallard_New_Jersey_Sg-00760_2008 | H4N6 | 9/21/08 | CY145742 | North American |
| A_mallard_New_Jersey_Sg-00884_2008 | H11N2 | 9/22/08 | CY145750 | North American |
| A_mallard_New_Jersey_Sg-00888_2008 | H3N2 | 9/23/08 | CY145758 | North American |
| A_mallard_New_Jersey_Sg-00892_2008 | H3N8 | 9/20/08 | CY145766 | North American |
| A_mallard_New_Jersey_Sg-00893_2008 | H3N2 | 9/23/08 | CY145774 | North American |
| A_mallard_New_Jersey_Sg-00895_2008 | H3N8 | 9/20/08 | CY145782 | North American |
| A_mallard_New_Jersey_Sg-00939_2008 | H4N6 | 9/21/08 | CY145798 | North American |
| A_mallard_New_Jersey_Sg-00941_2008 | H4N6 | 9/21/08 | CY145806 | North American |
| A_mallard_New_Jersey_Sg-00943_2008 | H4N6 | 9/21/08 | CY145824 | North American |
| A_mallard_New_Jersey_Sg-00965_2008 | H4N6 | 9/21/08 | CY145492 | North American |
| A_mallard_New_Jersey_Sg-00966_2008 | H4N6 | 9/21/08 | CY145832 | North American |
| A_mallard_New_Jersey_Sg-00967_2008 | H4N6 | 9/21/08 | CY145840 | North American |
| A_mallard_New_Zealand_1365-350_2005 | H6N9 | 2005 | CY077591 | North American |
| A_mallard_New_Zealand_1615-17_2004 | H4N6 | 2004 | CY045366 | North American |
| A_mallard_North_Dakota_Sg-00703_2008 | H12N4 | 9/2/08 | CY042951 | North American |
| A_mallard_North_Dakota_Sg-00703_2008 | H12N4 | 9/2/08 | CY140111 | North American |
| A_mallard_North_Dakota_Sg-00737_2008 | H6N8 | 9/5/08 | CY140143 | North American |
| A_mallard_Nova_Scotia_00084_2010 | H1N1 | 9/10/10 | CY138543 | North American |
| A_mallard_Nova_Scotia_00085_2010 | H1N1 | 9/10/10 | CY138551 | North American |
| A_mallard_Nova_Scotia_00088_2010 | H1N1 | 9/10/10 | CY138567 | North American |
| A_mallard_Nova_Scotia_00093_2010 | H1N1 | 9/10/10 | CY138607 | North American |
| A_mallard_Nova_Scotia_00346_2010 | H1N1 | 8/8/10 | CY138784 | North American |
| A_mallard_Nova_Scotia_00372_2010 | H7N7 | 8/8/10 | CY138808 | North American |
| A_mallard_Nova_Scotia_00375_2010 | H10N7 | 8/8/10 | CY138816 | North American |
| A_mallard_Nova_Scotia_00778_2010 | H3N8 | 9/7/10 | CY139262 | North American |
| A_mallard_Nova_Scotia_01013_2010 | H6N1 | 9/20/10 | CY139565 | North American |
| A_mallard_Nova_Scotia_01016_2010 | H3N8 | 9/20/10 | CY139573 | North American |
| A_mallard_Nova_Scotia_02151_2007 | H1N1 | 8/8/07 | CY128949 | North American |
| A_mallard_Nova_Scotia_02153_2007 | H1N1 | 8/8/07 | CY128925 | North American |
| A_mallard_Nova_Scotia_02286_2007 | H7N4 | 8/21/07 | CY128867 | North American |
| A_mallard_Nova_Scotia_02320_2007 | H4N6 | 8/25/07 | CY128893 | North American |
| A_mallard_Nova_Scotia_02335_2007 | H4N6 | 8/26/07 | CY128917 | North American |
| A_mallard_Nova_Scotia_02446_2007 | H4N6 | 8/28/07 | CY128819 | North American |
| A_mallard_Nova_Scotia_03271_2009 | H3N8 | 8/6/09 | CY125621 | North American |
| A_mallard_Ohio_08OS1270_2008 | H4N6 | 8/7/08 | CY132600 | North American |
| A_mallard_Ohio_10OS1319_2010 | H4N6 | 8/2/10 | CY132140 | North American |
| A_mallard_Ohio_10OS1354_2010 | H6N1 | 8/6/10 | CY132092 | North American |
| A_mallard_Ohio_10OS1467_2010 | H3N2 | 8/18/10 | CY132100 | North American |
| A_mallard_Ohio_10OS1469_2010 | H3N2 | 8/18/10 | CY132108 | North American |
| A_mallard_Ohio_10OS1470_2010 | H3N2 | 8/18/10 | CY132116 | North American |
| A_mallard_Ohio_1686_2009 | H6N9 | 8/10/09 | CY097108 | North American |
| A_mallard_Ohio_1688_2009 | H12N5 | 8/10/09 | CY097653 | North American |
| A_mallard_Ohio_1690_2009 | H6N1 | 8/10/09 | CY097116 | North American |
| A_mallard_Ohio_1695_2009 | H4N6 | 8/25/09 | CY097669 | North American |
| A_mallard_Ohio_2031_2009 | H4N9 | 8/3/09 | CY097661 | North American |
| A_mallard_Ohio_2033_2009 | H4N9 | 8/3/09 | CY097124 | North American |
| A_mallard_Ohio_2043_2009 | H6N1 | 8/3/09 | CY097142 | North American |
| A_mallard_Oregon_44221-105_2006 | H3N6 | 10/14/06 | CY076180 | North American |
| A_mallard_PT_35910-2_2006 | H4N6 | 10/30/06 | HM849024 | North American |
| A_mallard_Quebec_02916-1_2009 | H16N3 | 8/18/09 | CY125613 | North American |
| A_mallard_Quebec_10969_2006 | H2N3 | 8/18/06 | CY045286 | North American |
| A_mallard_Quebec_11002_2006 | H4N6 | 8/19/06 | CY047591 | North American |
| A_mallard_Quebec_11040_2006 | H3N2 | 8/19/06 | CY047599 | North American |
| A_mallard_Quebec_11045_2006 | H3N2 | 8/19/06 | CY047607 | North American |
| A_mallard_Quebec_11063_2006 | H2N3 | 8/19/06 | CY045294 | North American |
| A_mallard_Quebec_11082_2006 | H3N8 | 8/19/06 | CY047615 | North American |
| A_mallard_Quebec_11102_2006 | H4N6 | 8/20/06 | CY047583 | North American |
| A_mallard_Quebec_11106_2006 | H4N6 | 8/20/06 | CY047631 | North American |
| A_mallard_Quebec_11111_2006 | H11N9 | 8/20/06 | CY045302 | North American |
| A_mallard_Quebec_11121_2006 | H3N2 | 8/20/06 | CY047639 | North American |
| A_mallard_Quebec_11182_2006 | H4N6 | 8/24/06 | CY047647 | North American |
| A_mallard_Quebec_11194_2006 | H3N2 | 8/24/06 | CY047679 | North American |
| A_mallard_Quebec_11247_2006 | H3N2 | 8/24/06 | CY047663 | North American |
| A_mallard_Quebec_11281_2006 | H2N3 | 8/25/06 | CY047671 | North American |
| A_mallard_Washington_20010-002 | 2006_H3N8 | 10/1/06 | CY045374 | North American |
| A_mallard_Washington_44242-124_2006 | H3N2 | 7/23/06 | CY076188 | North American |
| A_mallard_Washington_44242-144_2006 | H4N6 | 8/14/06 | CY076196 | North American |
| A_mallard_Washington_44242-264_2006 | H5N2 | 8/10/06 | CY076204 | North American |
| A_mallard_Washington_44242-271_2006 | H4N6 | 8/15/06 | CY076212 | North American |
| A_mallard_Washington_44242-288_2006 | H4N6 | 8/18/06 | CY076220 | North American |
| A_mallard_Washington_44242-290_2006 | H4N6 | 8/16/06 | CY076228 | North American |
| A_mallard_Washington_44256-522_2006 | H11N3 | 11/8/06 | CY076332 | North American |
| A_mallard_Washington_44256-527_2006 | H11N9 | 11/8/06 | CY076340 | North American |
| A_mallard_Washington_44338-009_2007 | H6N1 | 8/29/07 | CY076492 | North American |
| A_mallard_Washington_44338-011_2007 | H4N6 | 8/29/07 | CY076500 | North American |
| A_mallard_Washington_44338-012_2007 | H4N6 | 8/29/07 | CY076508 | North American |
| A_mallard_Washington_44338-014_2007 | H4N6 | 8/29/07 | CY076516 | North American |
| A_mallard_Washington_44338-015_2007 | H4N6 | 8/29/07 | CY076524 | North American |
| A_mallard_Washington_44338-016_2007 | H4N6 | 8/29/07 | CY076532 | North American |
| A_mallard_Washington_44338-017_2007 | H4N6 | 8/29/07 | CY076540 | North American |
| A_mallard_Washington_44338-018_2007 | H4N6 | 8/29/07 | CY076548 | North American |
| A_mallard_Washington_44338-019_2007 | H6N1 | 8/29/07 | CY076556 | North American |
| A_mallard_Washington_44338-029_2007 | H6N1 | 8/29/07 | CY076564 | North American |
| A_mallard_Washington_44338-034_2007 | H6N1 | 9/4/07 | CY076572 | North American |
| A_mallard_Washington_44338-037_2007 | H6N1 | 9/4/07 | CY076580 | North American |
| A_mallard_Washington_44338-039_2007 | H6N1 | 9/4/07 | CY076588 | North American |
| A_mallard_Washington_44338-045_2007 | H6N1 | 9/4/07 | CY076596 | North American |
| A_mallard_Washington_44338-047_2007 | H6N1 | 9/4/07 | CY076604 | North American |
| A_mallard_Washington_44338-049_2007 | H6N1 | 9/4/07 | CY076612 | North American |
| A_mallard_Washington_44338-052_2007 | H3N1 | 9/12/07 | CY076620 | North American |
| A_mallard_Washington_44338-112_2007 | H6N2 | 9/10/07 | CY076628 | North American |
| A_mallard_Washington_44338-120_2007 | H6N2 | 9/10/07 | CY076636 | North American |
| A_mallard_Washington_44338-195_2007 | H12N5 | 11/20/07 | CY076068 | North American |
| A_mallard_Washington_44338-218_2007 | H1N1 | 11/15/07 | CY076644 | North American |
| A_mallard_Wisconsin_08OS2261_2008 | H4N6 | 10/19/08 | CY079331 | North American |
| A_mallard_Wisconsin_08OS2271_2008 | H11N9 | 10/19/08 | CY079339 | North American |
| A_mallard_Wisconsin_08OS2841_2008 | H2N3 | 10/31/08 | CY079483 | North American |
| A_mallard_Wisconsin_08OS2844_2008 | H2N3 | 10/31/08 | CY079499 | North American |
| A_mallard_Wisconsin_10OS1604_2010 | H5N2 | 9/25/10 | CY132696 | North American |
| A_mallard_Wisconsin_10OS2524_2010 | H11N2 | 9/26/10 | CY132932 | North American |
| A_mallard_Wisconsin_10OS2538_2010 | H3N2 | 9/26/10 | CY132940 | North American |
| A_mallard_Wisconsin_10OS2659_2010 | H4N2 | 10/2/10 | CY133076 | North American |
| A_mallard_Wisconsin_10OS2667_2010 | H11N9 | 10/1/10 | CY133068 | North American |
| A_mallard_Wisconsin_10OS2672_2010 | H5N1 | 10/2/10 | CY133084 | North American |
| A_mallard_Wisconsin_10OS2773_2010 | H3N8 | 9/26/10 | CY132956 | North American |
| A_mallard_Wisconsin_10OS2889_2010 | H11N9 | 10/3/10 | CY133192 | North American |
| A_mallard_Wisconsin_10OS2900_2010 | H3N2 | 10/3/10 | CY133200 | North American |
| A_mallard_Wisconsin_10OS2909_2010 | H3N8 | 10/3/10 | CY133208 | North American |
| A_mallard_Wisconsin_10OS2917_2010 | H4N6 | 10/3/10 | CY133216 | North American |
| A_mallard_Wisconsin_10OS2918_2010 | H4N6 | 10/3/10 | CY133224 | North American |
| A_mallard_Wisconsin_10OS3051_2010 | H4N2 | 10/3/10 | CY133249 | North American |
| A_mallard_Wisconsin_10OS3066_2010 | H6N2 | 10/3/10 | CY133257 | North American |
| A_mallard_Wisconsin_10OS3067_2010 | H4N2 | 10/3/10 | CY133265 | North American |
| A_mallard_Wisconsin_10OS3084_2010 | H3N8 | 10/3/10 | CY133144 | North American |
| A_mallard_Wisconsin_10OS3144_2010 | H6N1 | 10/29/10 | CY133168 | North American |
| A_mallard_Wisconsin_10OS3169_2010 | H4N9 | 10/29/10 | CY133176 | North American |
| A_mallard_Wisconsin_10OS3171_2010 | H7N3 | 10/30/10 | CY133321 | North American |
| A_mallard_Wisconsin_10OS3845_2010 | H5N2 | 11/12/10 | CY133339 | North American |
| A_mallard_Wisconsin_10OS3866_2010 | H3N6 | 11/13/10 | CY133347 | North American |
| A_mallard_Wisconsin_10OS3941_2010 | H14N6 | 11/18/10 | CY133273 | North American |
| A_mallard_Wisconsin_10OS4193_2010 | H11N9 | 11/30/10 | CY133052 | North American |
| A_mallard_Wisconsin_10OS4194_2010 | H11N9 | 11/30/10 | CY133060 | North American |
| A_mallard_Wisconsin_1534_2009 | H6N8 | 8/18/09 | CY097284 | North American |
| A_mallard_Wisconsin_1538_2009 | H4N6 | 8/18/09 | CY097292 | North American |
| A_mallard_Wisconsin_2530_2009 | H4N2 | 10/4/09 | CY097453 | North American |
| A_mallard_Wisconsin_2543_2009 | H3N2 | 10/5/09 | CY096993 | North American |
| A_mallard_Wisconsin_2549_2009 | H3N2 | 10/5/09 | CY097333 | North American |
| A_mallard_Wisconsin_2560_2009 | H2N3 | 10/5/09 | CY097341 | North American |
| A_mallard_Wisconsin_2575_2009 | H3N2 | 10/6/09 | CY097349 | North American |
| A_mallard_Wisconsin_2576_2009 | H5N1 | 10/6/09 | CY097357 | North American |
| A_mallard_Wisconsin_2653_2009 | H4N6 | 10/17/09 | CY097017 | North American |
| A_mallard_Wisconsin_2712_2009 | H3N6 | 10/11/09 | CY097041 | North American |
| A_mallard_Wisconsin_2719_2009 | H4N2 | 10/17/09 | CY097485 | North American |
| A_mallard_Wisconsin_2755_2009 | H1N1 | 10/30/09 | CY097009 | North American |
| A_mallard_Wisconsin_2756_2009 | H1N1 | 10/30/09 | CY097373 | North American |
| A_mallard_Wisconsin_2785_2009 | H2N3 | 10/31/09 | CY097381 | North American |
| A_mallard_Wisconsin_3165_2009 | H1N1 | 10/31/09 | CY097389 | North American |
| A_mallard_Wisconsin_4194_2009 | H1N1 | 12/3/09 | CY097397 | North American |
| A_mallard_Wisconsin_4197_2009 | H1N1 | 12/3/09 | CY097413 | North American |
| A_mallard_Wisconsin_4203_2009 | H11N9 | 12/3/09 | CY097429 | North American |
| A_mallard_Wisconsin_4218_2009 | H12N5 | 12/5/09 | CY097276 | North American |
| A_mallard_Wisconsin_4230_2009 | H10N1 | 12/6/09 | CY097308 | North American |
| A_mallard-black_duck_Hybrid_New_Brunswick_00904_2010 | H10N7 | 9/14/10 | CY139384 | North American |
| A_mallard-black_duck_Hybrid_New_Brunswick_02642_2007 | H3N8 | 9/23/07 | CY129397 | North American |
| A_mallard-black_duck_Hybrid_New_Brunswick_02657_2007 | H3N8 | 9/24/07 | CY129389 | North American |
| A_mallard-black_duck_Hybrid_New_Brunswick_02724_2007 | H4N6 | 9/19/07 | CY129405 | North American |
| A_mallard-black_duck_Hybrid_New_Brunswick_02748_2007 | H3N8 | 9/4/07 | CY128789 | North American |
| A_mallard-black_duck_Hybrid_New_Brunswick_03736_2009 | H13N6 | 9/14/09 | CY128965 | North American |
| A_mallard-black_duck_Hybrid_Nova_Scotia_02318_2007 | H4N6 | 8/25/07 | CY128827 | North American |
| A_mallard-black_duck_Hybrid_Nova_Scotia_02330_2007 | H4N6 | 8/26/07 | CY125653 | North American |
| A_Muscovy_duck_New_York_19495-7_2006 | H7N2 | 2/21/06 | CY036009 | North American |
| A_Muscovy_duck_New_York_62095-1_2006 | H5N2 | 5/15/06 | CY036041 | North American |
| A_northern_pintail_Alaska_7MP0344_2007 | H3N8 | 8/16/07 | CY043943 | North American |
| A_northern_pintail_Alaska_7MP0508_2007 | H3N8 | 9/9/07 | CY045454 | North American |
| A_northern_pintail_Alaska_7MP0608_2007 | H3N8 | 8/6/07 | CY043951 | North American |
| A_northern_pintail_Alaska_7MP1092_2007 | H3N8 | 9/10/07 | CY043991 | North American |
| A_northern_pintail_Alaska_7MP1393_2007 | H4N6 | 8/14/07 | CY043999 | North American |
| A_northern_pintail_Alberta_265_2007 | H4N6 | 8/4/07 | CY103347 | North American |
| A_northern_pintail_Alberta_8_2009 | H3N8 | 8/19/09 | CY103518 | North American |
| A_northern_pintail_California_2548_2010 | H11N2 | 10/23/10 | CY134398 | North American |
| A_northern_pintail_California_3452_2010 | H1N1 | 12/4/10 | CY120658 | North American |
| A_northern_pintail_California_3466_2010 | H1N3 | 12/8/10 | CY120666 | North American |
| A_northern_pintail_California_6495_2008 | H6N1 | 11/2/08 | CY093988 | North American |
| A_northern_pintail_California_6763_2008 | H6N1 | 11/15/08 | CY093996 | North American |
| A_northern_pintail_California_6791_2008 | H11N2 | 11/16/08 | CY094516 | North American |
| A_northern_pintail_California_8105_2008 | H6N1 | 10/19/08 | CY094004 | North American |
| A_northern_pintail_California_8152_2008 | H6N1 | 10/22/08 | CY094524 | North American |
| A_northern_pintail_California_8470_2008 | H6N1 | 10/22/08 | CY094532 | North American |
| A_northern_pintail_California_8764_2008 | H6N1 | 11/1/08 | CY094540 | North American |
| A_northern_pintail_California_HKWF151_2007 | H6N1 | 10/24/07 | CY035846 | North American |
| A_northern_pintail_California_HKWF440C_2007 | H6N1 | 11/4/07 | CY094012 | North American |
| A_northern_pintail_California_HKWF792_2007 | H3N8 | 11/28/07 | CY032717 | North American |
| A_northern_pintail_Illinois_10OS3959_2010 | H7N3 | 11/21/10 | CY132884 | North American |
| A_northern_pintail_Interior_Alaska_1_2007 | H3N8 | 8/7/07 | CY039775 | North American |
| A_northern_pintail_Interior_Alaska_1_2007 | H4N6 | 8/8/07 | CY036648 | North American |
| A_northern_pintail_Interior_Alaska_1_2007 | H1N1 | 8/11/07 | CY039744 | North American |
| A_northern_pintail_Interior_Alaska_10BM00303R0_2010 | H3N8 | 5/14/10 | CY130420 | North American |
| A_northern_pintail_Interior_Alaska_10BM00849R2_2010 | H3N1 | 5/23/10 | CY130484 | North American |
| A_northern_pintail_Interior_Alaska_10BM02539R0_2010 | H7N3 | 6/22/10 | CY130395 | North American |
| A_northern_pintail_Interior_Alaska_10BM02585R0_2010 | H4N6 | 6/22/10 | CY130428 | North American |
| A_northern_pintail_Interior_Alaska_10BM02791R0_2010 | H4N6 | 6/25/10 | CY130452 | North American |
| A_northern_pintail_Interior_Alaska_10BM04668R0_2010 | H4N6 | 7/18/10 | CY135638 | North American |
| A_northern_pintail_Interior_Alaska_10BM04704R0_2010 | H4N6 | 7/18/10 | CY135646 | North American |
| A_northern_pintail_Interior_Alaska_10BM04722R0_2010 | H4N6 | 7/18/10 | CY135654 | North American |
| A_northern_pintail_Interior_Alaska_10BM05034R0_2010 | H3N6 | 7/21/10 | CY135670 | North American |
| A_northern_pintail_Interior_Alaska_10BM05171R0_2010 | H4N6 | 7/22/10 | CY135686 | North American |
| A_northern_pintail_Interior_Alaska_10BM06303R0_2010 | H7N3 | 7/29/10 | CY135858 | North American |
| A_northern_pintail_Interior_Alaska_10BM06306R0_2010 | H7N3 | 7/29/10 | CY135866 | North American |
| A_northern_pintail_Interior_Alaska_10BM06524R0_2010 | H7N3 | 7/30/10 | CY135946 | North American |
| A_northern_pintail_Interior_Alaska_10BM06704R0_2010 | H3N8 | 7/30/10 | CY136034 | North American |
| A_northern_pintail_Interior_Alaska_10BM06720R0_2010 | H7N3 | 7/30/10 | CY136042 | North American |
| A_northern_pintail_Interior_Alaska_10BM06872R0_2010 | H3N8 | 7/31/10 | CY135978 | North American |
| A_northern_pintail_Interior_Alaska_10BM06895R0_2010 | H7N3 | 7/31/10 | CY135986 | North American |
| A_northern_pintail_Interior_Alaska_10BM06899R0_2010 | H7N3 | 7/31/10 | CY135994 | North American |
| A_northern_pintail_Interior_Alaska_10BM07040R0_2010 | H4N6 | 8/1/10 | CY143467 | North American |
| A_northern_pintail_Interior_Alaska_10BM07242R0_2010 | H4N6 | 8/1/10 | CY143475 | North American |
| A_northern_pintail_Interior_Alaska_10BM07476R0_2010 | H4N6 | 8/2/10 | CY143483 | North American |
| A_northern_pintail_Interior_Alaska_10BM07597R0_2010 | H3N8 | 8/2/10 | CY143499 | North American |
| A_northern_pintail_Interior_Alaska_10BM07993R0_2010 | H4N6 | 8/3/10 | CY143547 | North American |
| A_northern_pintail_Interior_Alaska_10BM08488R0_2010 | H3N8 | 8/4/10 | CY143595 | North American |
| A_northern_pintail_Interior_Alaska_10BM08489R0_2010 | H3N8 | 8/4/10 | CY143603 | North American |
| A_northern_pintail_Interior_Alaska_10BM08493R0_2010 | H3N8 | 8/4/10 | CY143611 | North American |
| A_northern_pintail_Interior_Alaska_10BM08505R0_2010 | H3N8 | 8/4/10 | CY143619 | North American |
| A_northern_pintail_Interior_Alaska_10BM08514R0_2010 | H4N6 | 8/4/10 | CY143627 | North American |
| A_northern_pintail_Interior_Alaska_10BM08540R0_2010 | H4N6 | 8/4/10 | CY143635 | North American |
| A_northern_pintail_Interior_Alaska_10BM08587R0_2010 | H3N8 | 8/4/10 | CY143643 | North American |
| A_northern_pintail_Interior_Alaska_10BM08612R0_2010 | H4N6 | 8/4/10 | CY143651 | North American |
| A_northern_pintail_Interior_Alaska_10BM08899R0_2010 | H4N6 | 8/5/10 | CY143675 | North American |
| A_northern_pintail_Interior_Alaska_10BM08903R0_2010 | H4N6 | 8/5/10 | CY143683 | North American |
| A_northern_pintail_Interior_Alaska_10BM08920R0_2010 | H3N8 | 8/5/10 | CY143691 | North American |
| A_northern_pintail_Interior_Alaska_10BM09101R0_2010 | H3N8 | 8/5/10 | CY143779 | North American |
| A_northern_pintail_Interior_Alaska_10BM09102R0_2010 | H3N8 | 8/5/10 | CY143787 | North American |
| A_northern_pintail_Interior_Alaska_10BM09104R0_2010 | H3N8 | 8/5/10 | CY143795 | North American |
| A_northern_pintail_Interior_Alaska_10BM09105R0_2010 | H3N8 | 8/5/10 | CY143803 | North American |
| A_northern_pintail_Interior_Alaska_10BM10298R0_2010 | H3N8 | 8/9/10 | CY143835 | North American |
| A_northern_pintail_Interior_Alaska_10BM10480R0_2010 | H3N8 | 8/9/10 | CY143843 | North American |
| A_northern_pintail_Interior_Alaska_10BM10928R0_2010 | H3N8 | 8/11/10 | CY143876 | North American |
| A_northern_pintail_Interior_Alaska_10BM11672R0_2010 | H3N8 | 8/13/10 | CY143966 | North American |
| A_northern_pintail_Interior_Alaska_10BM11694R0_2010 | H3N8 | 8/13/10 | CY143974 | North American |
| A_northern_pintail_Interior_Alaska_10BM11989R0_2010 | H3N8 | 8/13/10 | CY144094 | North American |
| A_northern_pintail_Interior_Alaska_10BM12146R0_2010 | H4N6 | 8/13/10 | CY144110 | North American |
| A_northern_pintail_Interior_Alaska_10BM12163R0_2010 | H3N8 | 8/13/10 | CY143982 | North American |
| A_northern_pintail_Interior_Alaska_10BM12221R0_2010 | H4N6 | 8/13/10 | CY141506 | North American |
| A_northern_pintail_Interior_Alaska_10BM12552R0_2010 | H3N8 | 8/14/10 | CY144118 | North American |
| A_northern_pintail_Interior_Alaska_10BM12603R0_2010 | H3N8 | 8/14/10 | CY144126 | North American |
| A_northern_pintail_Interior_Alaska_10BM14807R2_2010 | H9N2 | 8/18/10 | CY125757 | North American |
| A_northern_pintail_Interior_Alaska_10CH00003R0_2010 | H4N6 | 9/4/10 | CY143755 | North American |
| A_northern_pintail_Interior_Alaska_2_2007 | H4N8 | 9/7/07 | CY038367 | North American |
| A_northern_pintail_Interior_Alaska_6MP0741R2_2006 | H4N6 | 8/8/06 | CY078714 | North American |
| A_northern_pintail_Interior_Alaska_6MP0792_2006 | H2N3 | 8/10/06 | CY078586 | North American |
| A_northern_pintail_Interior_Alaska_6MP0814_2006 | H3N8 | 8/12/06 | CY078538 | North American |
| A_northern_pintail_Interior_Alaska_6MP0964_2006 | H3N8 | 8/17/06 | CY078642 | North American |
| A_northern_pintail_Interior_Alaska_6MP1080R1_2006 | H3N8 | 8/20/06 | CY078690 | North American |
| A_northern_pintail_Interior_Alaska_7MP0278_2007 | H1N1 | 8/17/07 | CY077317 | North American |
| A_northern_pintail_Interior_Alaska_7MP0343_2007 | H3N8 | 8/16/07 | CY045414 | North American |
| A_northern_pintail_Interior_Alaska_7MP0345BR2_2007 | H3N8 | 8/16/07 | CY078906 | North American |
| A_northern_pintail_Interior_Alaska_7MP0408_2007 | H3N8 | 8/18/07 | CY078946 | North American |
| A_northern_pintail_Interior_Alaska_7MP0509_2007 | H3N8 | 9/6/07 | CY078914 | North American |
| A_northern_pintail_Interior_Alaska_7MP0692_2007 | H1N1 | 8/8/07 | CY077324 | North American |
| A_northern_pintail_Interior_Alaska_7MP1067R1_2007 | H4N5 | 9/15/07 | CY077411 | North American |
| A_northern_pintail_Interior_Alaska_7MP1246_2007 | H4N6 | 8/8/07 | CY077221 | North American |
| A_northern_pintail_Interior_Alaska_7MP1460R2_2007 | H3N8 | 8/16/07 | CY079050 | North American |
| A_northern_pintail_Interior_Alaska_7MP1644_2007 | H4N6 | 9/2/07 | CY077371 | North American |
| A_northern_pintail_Interior_Alaska_7MP1822_2007 | H3N8 | 9/5/07 | CY079010 | North American |
| A_northern_pintail_Interior_Alaska_7MP2228_2007 | H3N8 | 9/16/07 | CY079026 | North American |
| A_northern_pintail_Interior_Alaska_8BM1995R1_2008 | H12N5 | 8/9/08 | CY079621 | North American |
| A_northern_pintail_Interior_Alaska_8BM2046R1_2008 | H8N4 | 8/9/08 | CY079387 | North American |
| A_northern_pintail_Interior_Alaska_8BM2082_2008 | H8N4 | 8/9/08 | CY080114 | North American |
| A_northern_pintail_Interior_Alaska_8BM2528R1_2008 | H3N8 | 8/13/08 | CY079066 | North American |
| A_northern_pintail_Interior_Alaska_8BM2621R1_2008 | H8N4 | 8/14/08 | CY079629 | North American |
| A_northern_pintail_Interior_Alaska_8BM2974_2008 | H12N5 | 8/17/08 | CY079082 | North American |
| A_northern_pintail_Interior_Alaska_8BM2987_2008 | H8N4 | 8/17/08 | CY079090 | North American |
| A_northern_pintail_Interior_Alaska_8BM3041_2008 | H8N4 | 8/17/08 | CY079098 | North American |
| A_northern_pintail_Interior_Alaska_8BM3088_2008 | H8N4 | 8/18/08 | CY079114 | North American |
| A_northern_pintail_Interior_Alaska_8BM3091_2008 | H8N4 | 8/18/08 | CY079637 | North American |
| A_northern_pintail_Interior_Alaska_8BM3137_2008 | H8N4 | 8/18/08 | CY079645 | North American |
| A_northern_pintail_Interior_Alaska_8BM3582_2008 | H3N8 | 9/14/08 | CY079122 | North American |
| A_northern_pintail_Interior_Alaska_8BM3608_2008 | H4N6 | 9/15/08 | CY079668 | North American |
| A_northern_pintail_Interior_Alaska_8BM3658_2008 | H4N6 | 9/24/08 | CY079146 | North American |
| A_northern_pintail_Interior_Alaska_8BM3696_2008 | H3N8 | 9/26/08 | CY080099 | North American |
| A_northern_pintail_Interior_Alaska_8BM3700_2008 | H3N8 | 9/26/08 | CY079162 | North American |
| A_northern_pintail_Interior_Alaska_8BM3723_2008 | H4N1 | 9/27/08 | CY079676 | North American |
| A_northern_pintail_Interior_Alaska_8BM3731_2008 | H3N8 | 9/27/08 | CY079178 | North American |
| A_northern_pintail_Interior_Alaska_8BM3736_2008 | H12N5 | 9/27/08 | CY079186 | North American |
| A_northern_pintail_Interior_Alaska_8MP0213R2_2008 | H8N4 | 8/7/08 | CY080137 | North American |
| A_northern_pintail_Interior_Alaska_8MP0262R2_2008 | H7N3 | 8/8/08 | CY080214 | North American |
| A_northern_pintail_Interior_Alaska_8MP0689_2008 | H8N4 | 8/13/08 | CY079058 | North American |
| A_northern_pintail_Interior_Alaska_9BM10471R0_2009 | H3N8 | 8/16/09 | CY143411 | North American |
| A_northern_pintail_Interior_Alaska_9BM10749R0_2009 | H4N6 | 8/17/09 | CY143057 | North American |
| A_northern_pintail_Interior_Alaska_9BM10813R0_2009 | H4N6 | 8/17/09 | CY143065 | North American |
| A_northern_pintail_Interior_Alaska_9BM10819R0_2009 | H3N8 | 8/17/09 | CY143073 | North American |
| A_northern_pintail_Interior_Alaska_9BM10835R0_2009 | H4N6 | 8/17/09 | CY143081 | North American |
| A_northern_pintail_Interior_Alaska_9BM10877R0_2009 | H4N6 | 8/17/09 | CY142769 | North American |
| A_northern_pintail_Interior_Alaska_9BM10969R0_2009 | H3N8 | 8/17/09 | CY142777 | North American |
| A_northern_pintail_Interior_Alaska_9BM10987R0_2009 | H3N8 | 8/17/09 | CY142785 | North American |
| A_northern_pintail_Interior_Alaska_9BM11231R0_2009 | H3N8 | 8/18/09 | CY142801 | North American |
| A_northern_pintail_Interior_Alaska_9BM11487R0_2009 | H3N8 | 8/18/09 | CY142817 | North American |
| A_northern_pintail_Interior_Alaska_9BM11572R0_2009 | H3N8 | 8/20/09 | CY142833 | North American |
| A_northern_pintail_Interior_Alaska_9BM11719R1_2009 | H4N6 | 8/19/09 | CY142841 | North American |
| A_northern_pintail_Interior_Alaska_9BM11757R0_2009 | H3N8 | 8/19/09 | CY142849 | North American |
| A_northern_pintail_Interior_Alaska_9BM11789R0_2009 | H3N8 | 8/19/09 | CY142857 | North American |
| A_northern_pintail_Interior_Alaska_9BM11911R0_2009 | H3N8 | 8/19/09 | CY142873 | North American |
| A_northern_pintail_Interior_Alaska_9BM11915R0_2009 | H4N6 | 8/19/09 | CY142881 | North American |
| A_northern_pintail_Interior_Alaska_9BM12066R1_2009 | H3N8 | 8/20/09 | CY142897 | North American |
| A_northern_pintail_Interior_Alaska_9BM12176R0_2009 | H3N8 | 8/20/09 | CY143097 | North American |
| A_northern_pintail_Interior_Alaska_9BM12180R0_2009 | H3N8 | 8/20/09 | CY143105 | North American |
| A_northern_pintail_Interior_Alaska_9BM12186R0_2009 | H4N6 | 8/21/09 | CY143113 | North American |
| A_northern_pintail_Interior_Alaska_9BM12190R0_2009 | H4N6 | 8/21/09 | CY143121 | North American |
| A_northern_pintail_Interior_Alaska_9BM12210R0_2009 | H3N8 | 8/21/09 | CY143145 | North American |
| A_northern_pintail_Interior_Alaska_9BM12211R0_2009 | H3N8 | 8/21/09 | CY143419 | North American |
| A_northern_pintail_Interior_Alaska_9BM12464R0_2009 | H3N8 | 9/2/09 | CY143225 | North American |
| A_northern_pintail_Interior_Alaska_9BM12647R0_2009 | H3N8 | 9/5/09 | CY143329 | North American |
| A_northern_pintail_Interior_Alaska_9BM12700R0_2009 | H3N8 | 9/6/09 | CY143347 | North American |
| A_northern_pintail_Interior_Alaska_9BM3859R1_2009 | H10N7 | 7/24/09 | CY135470 | North American |
| A_northern_pintail_Interior_Alaska_9BM4347R0_2009 | H4N6 | 7/27/09 | CY135374 | North American |
| A_northern_pintail_Interior_Alaska_9BM4410R1_2009 | H10N7 | 7/28/09 | CY135478 | North American |
| A_northern_pintail_Interior_Alaska_9BM4583R0_2009 | H4N6 | 7/29/09 | CY135414 | North American |
| A_northern_pintail_Interior_Alaska_9BM4637R0_2009 | H10N7 | 7/29/09 | CY135422 | North American |
| A_northern_pintail_Interior_Alaska_9BM4641R0_2009 | H4N6 | 7/29/09 | CY135430 | North American |
| A_northern_pintail_Interior_Alaska_9BM4646R0_2009 | H10N7 | 7/29/09 | CY135438 | North American |
| A_northern_pintail_Interior_Alaska_9BM4660R0_2009 | H4N6 | 7/29/09 | CY135446 | North American |
| A_northern_pintail_Interior_Alaska_9BM4666R0_2009 | H10N7 | 7/29/09 | CY135454 | North American |
| A_northern_pintail_Interior_Alaska_9BM4989R0_2009 | H10N7 | 7/29/09 | CY135518 | North American |
| A_northern_pintail_Interior_Alaska_9BM4991R0_2009 | H10N7 | 7/29/09 | CY135526 | North American |
| A_northern_pintail_Interior_Alaska_9BM5053R0_2009 | H10N7 | 7/29/09 | CY135550 | North American |
| A_northern_pintail_Interior_Alaska_9BM5054R0_2009 | H10N7 | 7/29/09 | CY135196 | North American |
| A_northern_pintail_Interior_Alaska_9BM5375R0_2009 | H10N7 | 7/30/09 | CY135566 | North American |
| A_northern_pintail_Interior_Alaska_9BM5376R0_2009 | H10N7 | 7/30/09 | CY135574 | North American |
| A_northern_pintail_Interior_Alaska_9BM5554R0_2009 | H10N7 | 7/31/09 | CY135582 | North American |
| A_northern_pintail_Interior_Alaska_9BM5698R0_2009 | H10N7 | 7/31/09 | CY135590 | North American |
| A_northern_pintail_Interior_Alaska_9BM6039R0_2009 | H4N6 | 8/2/09 | CY143427 | North American |
| A_northern_pintail_Interior_Alaska_9BM6040R0_2009 | H4N6 | 8/2/09 | CY143363 | North American |
| A_northern_pintail_Interior_Alaska_9BM6094R0_2009 | H4N6 | 8/2/09 | CY143435 | North American |
| A_northern_pintail_Interior_Alaska_9BM6095R0_2009 | H4N6 | 8/2/09 | CY143443 | North American |
| A_northern_pintail_Interior_Alaska_9BM6144R0_2009 | H3N8 | 8/2/09 | CY142593 | North American |
| A_northern_pintail_Interior_Alaska_9BM6145R0_2009 | H3N8 | 8/2/09 | CY143371 | North American |
| A_northern_pintail_Interior_Alaska_9BM6146R0_2009 | H3N8 | 8/2/09 | CY142601 | North American |
| A_northern_pintail_Interior_Alaska_9BM6248R0_2009 | H4N6 | 8/3/09 | CY142609 | North American |
| A_northern_pintail_Interior_Alaska_9BM6290R0_2009 | H3N8 | 8/3/09 | CY142625 | North American |
| A_northern_pintail_Interior_Alaska_9BM6494R0_2009 | H4N6 | 8/3/09 | CY142641 | North American |
| A_northern_pintail_Interior_Alaska_9BM6902R0_2009 | H3N8 | 8/5/09 | CY142657 | North American |
| A_northern_pintail_Interior_Alaska_9BM7044R0_2009 | H3N8 | 8/6/09 | CY142665 | North American |
| A_northern_pintail_Interior_Alaska_9BM7050R0_2009 | H3N8 | 8/6/09 | CY142673 | North American |
| A_northern_pintail_Interior_Alaska_9BM7052R0_2009 | H3N8 | 8/6/09 | CY142905 | North American |
| A_northern_pintail_Interior_Alaska_9BM7055R0_2009 | H3N8 | 8/6/09 | CY143379 | North American |
| A_northern_pintail_Interior_Alaska_9BM7069R0_2009 | H3N8 | 8/6/09 | CY143387 | North American |
| A_northern_pintail_Interior_Alaska_9BM7118R1_2009 | H3N8 | 8/6/09 | CY142681 | North American |
| A_northern_pintail_Interior_Alaska_9BM7232R0_2009 | H4N6 | 8/6/09 | CY142689 | North American |
| A_northern_pintail_Interior_Alaska_9BM7252R0_2009 | H3N8 | 8/6/09 | CY142697 | North American |
| A_northern_pintail_Interior_Alaska_9BM7254R0_2009 | H3N8 | 8/6/09 | CY142705 | North American |
| A_northern_pintail_Interior_Alaska_9BM7258R0_2009 | H3N8 | 8/6/09 | CY142713 | North American |
| A_northern_pintail_Interior_Alaska_9BM7302R0_2009 | H4N6 | 8/7/09 | CY142721 | North American |
| A_northern_pintail_Interior_Alaska_9BM7368R0_2009 | H3N8 | 8/7/09 | CY142913 | North American |
| A_northern_pintail_Interior_Alaska_9BM7496R0_2009 | H4N6 | 8/8/09 | CY142921 | North American |
| A_northern_pintail_Interior_Alaska_9BM7620R0_2009 | H4N6 | 8/8/09 | CY142929 | North American |
| A_northern_pintail_Interior_Alaska_9BM7632R0_2009 | H4N6 | 8/8/09 | CY142937 | North American |
| A_northern_pintail_Interior_Alaska_9BM7836R0_2009 | H3N8 | 8/8/09 | CY142945 | North American |
| A_northern_pintail_Interior_Alaska_9BM7934R0_2009 | H4N6 | 8/8/09 | CY142953 | North American |
| A_northern_pintail_Interior_Alaska_9BM7972R0_2009 | H4N6 | 8/9/09 | CY143395 | North American |
| A_northern_pintail_Interior_Alaska_9BM8022R0_2009 | H4N6 | 8/9/09 | CY142961 | North American |
| A_northern_pintail_Interior_Alaska_9BM8119R0_2009 | H4N6 | 8/9/09 | CY142969 | North American |
| A_northern_pintail_Interior_Alaska_9BM8415R2_2009 | H4N6 | 8/9/09 | CY142985 | North American |
| A_northern_pintail_Interior_Alaska_9BM8553R0_2009 | H3N8 | 8/10/09 | CY143001 | North American |
| A_northern_pintail_Interior_Alaska_9BM8657R1_2009 | H3N8 | 8/10/09 | CY143355 | North American |
| A_northern_pintail_Interior_Alaska_9BM8803R0_2009 | H4N6 | 8/11/09 | CY143009 | North American |
| A_northern_pintail_Interior_Alaska_9BM8815R0_2009 | H4N6 | 8/11/09 | CY143017 | North American |
| A_northern_pintail_Interior_Alaska_9BM8907R0_2009 | H3N8 | 8/11/09 | CY143033 | North American |
| A_northern_pintail_Interior_Alaska_9BM9447R0_2009 | H3N8 | 8/13/09 | CY143249 | North American |
| A_northern_pintail_Interior_Alaska_9BM9611R0_2009 | H3N8 | 8/13/09 | CY143257 | North American |
| A_northern_pintail_Interior_Alaska_9BM9975R0_2009 | H4N6 | 8/15/09 | CY143281 | North American |
| A_northern_pintail_Interior_Alaska_9BM9989R0_2009 | H4N6 | 8/15/09 | CY143305 | North American |
| A_northern_pintail_Louisiana_Sg-01033_2008 | H4N8 | 11/9/08 | CY140558 | North American |
| A_northern_pintail_Minnesota_AI09-3254_2009 | H6N2 | 9/8/09 | CY140819 | North American |
| A_northern_pintail_Minnesota_AI09-4322_2009 | H3N8 | 9/12/09 | CY140942 | North American |
| A_northern_pintail_Missouri_319_2009 | H12N5 | 12/6/09 | CY097645 | North American |
| A_northern_pintail_New_Brunswick_03547_2009 | H3N8 | 9/11/09 | CY125364 | North American |
| A_northern_pintail_Oregon_44249-559_2006 | H3N8 | 8/22/06 | CY076244 | North American |
| A_northern_pintail_Saskatchewan_22910_2007 | H3N2 | 8/10/07 | CY045326 | North American |
| A_northern_pintail_Wisconsin_10OS2857_2010 | H5N2 | 10/2/10 | CY133100 | North American |
| A_northern_pintail_Wisconsin_2737_2009 | H3N2 | 10/17/09 | CY097501 | North American |
| A_northern_pintail_Wisconsin_4198_2009 | H11N9 | 12/3/09 | CY097421 | North American |
| A_northern_shoveler_Alaska_7MP0954_2007 | H4N6 | 9/6/07 | CY044023 | North American |
| A_northern_shoveler_Alaska_7MP1026_2007 | H3N8 | 9/7/07 | CY043967 | North American |
| A_northern_shoveler_Alaska_7MP1606_2007 | H3N8 | 9/4/07 | CY044015 | North American |
| A_northern_shoveler_Alaska_7MP1668_2007 | H3N8 | 9/7/07 | CY044031 | North American |
| A_northern_shoveler_Alaska_7MP1669B_2007 | H3N8 | 9/2/07 | CY077348 | North American |
| A_northern_shoveler_Alaska_7MP1708_2007 | H3N8 | 9/3/07 | CY045446 | North American |
| A_northern_shoveler_Arkansas_AI09-6012_2009 | H10N3 | 12/10/09 | CY141048 | North American |
| A_northern_shoveler_California_10024_2008 | H4N4 | 12/21/08 | CY094348 | North American |
| A_northern_shoveler_California_27820_2007 | H7N3 | 1/24/07 | CY075988 | North American |
| A_northern_shoveler_California_27943_2007 | H10N7 | 1/24/07 | CY076372 | North American |
| A_northern_shoveler_California_27985_2007 | H7N6 | 1/24/07 | CY076396 | North American |
| A_northern_shoveler_California_28327_2007 | H7N3 | 1/27/07 | CY076428 | North American |
| A_northern_shoveler_California_3046_2010 | H4N3 | 11/24/10 | CY120610 | North American |
| A_northern_shoveler_California_3183_2010 | H11N2 | 11/27/10 | CY120642 | North American |
| A_northern_shoveler_California_3483_2010 | H12N5 | 12/8/10 | CY120674 | North American |
| A_northern_shoveler_California_3676_2010 | H8N2 | 12/15/10 | CY134302 | North American |
| A_northern_shoveler_California_44241-862_2006 | H11N9 | 12/2/06 | CY075964 | North American |
| A_northern_shoveler_California_44287-088_2007 | H10N7 | 1/24/07 | CY075996 | North American |
| A_northern_shoveler_California_44287-162_2007 | H10N7 | 1/24/07 | CY076380 | North American |
| A_northern_shoveler_California_44287-164_2007 | H7N7 | 1/24/07 | CY076388 | North American |
| A_northern_shoveler_California_44287-179_2007 | H7N6 | 1/24/07 | CY076404 | North American |
| A_northern_shoveler_California_44287-364_2007 | H7N3 | 1/27/07 | CY076436 | North American |
| A_northern_shoveler_California_44363-082_2007 | H11N9 | 11/4/07 | CY076700 | North American |
| A_northern_shoveler_California_6535_2009 | H9N2 | 10/24/09 | CY157509 | North American |
| A_northern_shoveler_California_6535_2009 | H9N2 | 10/24/09 | CY157509 | North American |
| A_northern_shoveler_California_8355_2008 | H6N1 | 10/19/08 | CY093733 | North American |
| A_northern_shoveler_California_8528_2008 | H6N1 | 10/25/08 | CY094276 | North American |
| A_northern_shoveler_California_8536_2008 | H6N1 | 10/25/08 | CY094284 | North American |
| A_northern_shoveler_California_8629_2008 | H6N1 | 10/29/08 | CY094292 | North American |
| A_northern_shoveler_California_8673_2008 | H6N1 | 10/29/08 | CY093741 | North American |
| A_northern_shoveler_California_8675_2008 | H11N9 | 10/29/08 | CY094300 | North American |
| A_northern_shoveler_California_9017_2008 | H11N2 | 11/12/08 | CY094308 | North American |
| A_northern_shoveler_California_9140_2008 | H1N9 | 11/19/08 | CY093765 | North American |
| A_northern_shoveler_California_9187_2008 | H6N2 | 11/22/08 | CY094316 | North American |
| A_northern_shoveler_California_9228_2008 | H4N6 | 11/22/08 | CY094324 | North American |
| A_northern_shoveler_California_9235_2008 | H10N8 | 11/22/08 | CY093773 | North American |
| A_northern_shoveler_California_9267_2008 | H4N6 | 11/23/08 | CY094332 | North American |
| A_northern_shoveler_California_9680_2008 | H6N2 | 12/7/08 | CY093781 | North American |
| A_northern_shoveler_California_9710_2008 | H10N7 | 12/7/08 | CY093789 | North American |
| A_northern_shoveler_California_9781_2008 | H1N3 | 12/14/08 | CY094340 | North American |
| A_northern_shoveler_California_AKS273_2007 | H8N4 | 12/1/07 | CY039545 | North American |
| A_northern_shoveler_California_HKWF1005_2007 | H10N3 | 11/2/07 | CY032653 | North American |
| A_northern_shoveler_California_HKWF1021_2007 | H3N7 | 12/2/07 | CY033321 | North American |
| A_northern_shoveler_California_HKWF1026_2007 | H7N3 | 12/2/07 | CY039577 | North American |
| A_northern_shoveler_California_HKWF1046C_2007 | H3N5 | 12/2/07 | CY094364 | North American |
| A_northern_shoveler_California_HKWF1128C_2007 | H2N7 | 12/2/07 | CY094372 | North American |
| A_northern_shoveler_California_HKWF1131_2007 | H3N5 | 12/2/07 | CY033345 | North American |
| A_northern_shoveler_California_HKWF115_2007 | H6N1 | 10/24/07 | CY032881 | North American |
| A_northern_shoveler_California_HKWF1199_2007 | H3N5 | 12/5/07 | CY033361 | North American |
| A_northern_shoveler_California_HKWF1201_2007 | H3N5 | 12/5/07 | CY032669 | North American |
| A_northern_shoveler_California_HKWF1203_2007 | H8N4 | 12/5/07 | CY034148 | North American |
| A_northern_shoveler_California_HKWF1204_2007 | H8N4 | 12/5/07 | CY039585 | North American |
| A_northern_shoveler_California_HKWF1325_2007 | H8N4 | 12/9/07 | CY034156 | North American |
| A_northern_shoveler_California_HKWF1370_2007 | H10N3 | 12/12/07 | CY032685 | North American |
| A_northern_shoveler_California_HKWF1372C_2007 | H7N3 | 12/12/07 | CY094380 | North American |
| A_northern_shoveler_California_HKWF2031_2008 | H7N3 | 1/23/08 | CY039593 | North American |
| A_northern_shoveler_California_HKWF216_2007 | H6N1 | 10/28/07 | CY035854 | North American |
| A_northern_shoveler_California_HKWF268_2007 | H6N2 | 10/28/07 | CY032693 | North American |
| A_northern_shoveler_California_HKWF383_2007 | H6N1 | 11/3/07 | CY032709 | North American |
| A_northern_shoveler_California_HKWF392sm_2007 | H10N7 | 11/3/07 | CY039601 | North American |
| A_northern_shoveler_California_HKWF569_2007 | H3N1 | 11/10/07 | CY039617 | North American |
| A_northern_shoveler_California_HKWF592C_2007 | H10N7 | 11/10/07 | CY094356 | North American |
| A_northern_shoveler_California_HKWF608_2007 | H10N7 | 11/10/07 | CY039625 | North American |
| A_northern_shoveler_California_HKWF611_2007 | H11N9 | 11/10/07 | CY032913 | North American |
| A_northern_shoveler_California_HKWF96_2007 | H10N7 | 10/24/07 | CY039569 | North American |
| A_northern_shoveler_California_HKWF979_2007 | H3N3 | 12/2/07 | CY032725 | North American |
| A_northern_shoveler_California_JN1447_2007 | H7N2 | 2/3/07 | CY076879 | North American |
| A_northern_shoveler_California_JN587_2006 | H10N3 | 12/6/06 | CY053796 | North American |
| A_northern_shoveler_California_JN950_2006 | H10N7 | 12/6/06 | CY076839 | North American |
| A_northern_shoveler_Illinois_08OS3331_2008 | H4N8 | 11/15/08 | CY079515 | North American |
| A_northern_shoveler_Illinois_10OS3619_2010 | H11N9 | 10/30/10 | CY133036 | North American |
| A_northern_shoveler_Interior_Alaska_1_2007 | H4N6 | 9/1/07 | CY039831 | North American |
| A_northern_shoveler_Interior_Alaska_1_2007 | H3N8 | 9/6/07 | CY035812 | North American |
| A_northern_shoveler_Interior_Alaska_1_2007 | H12N5 | 11/6/07 | CY038351 | North American |
| A_northern_shoveler_Interior_Alaska_10BM02593R0_2010 | H4N6 | 6/23/10 | CY130436 | North American |
| A_northern_shoveler_Interior_Alaska_10BM02892R0_2010 | H4N6 | 6/30/10 | CY130412 | North American |
| A_northern_shoveler_Interior_Alaska_10BM05382R0_2010 | H3N8 | 7/25/10 | CY135744 | North American |
| A_northern_shoveler_Interior_Alaska_10BM05408R0_2010 | H4N6 | 7/25/10 | CY135752 | North American |
| A_northern_shoveler_Interior_Alaska_10BM05487R0_2010 | H3N8 | 7/26/10 | CY135770 | North American |
| A_northern_shoveler_Interior_Alaska_10BM05491R0_2010 | H3N8 | 7/26/10 | CY135778 | North American |
| A_northern_shoveler_Interior_Alaska_10BM05638R0_2010 | H3N8 | 7/26/10 | CY135794 | North American |
| A_northern_shoveler_Interior_Alaska_10BM05649R0_2010 | H3N8 | 7/26/10 | CY135802 | North American |
| A_northern_shoveler_Interior_Alaska_10BM16764R0_2010 | H9N2 | 9/8/10 | CY125765 | North American |
| A_northern_shoveler_Interior_Alaska_2_2007 | H3N8 | 9/6/07 | CY039760 | North American |
| A_northern_shoveler_Interior_Alaska_3_2007 | H3N8 | 9/7/07 | CY039791 | North American |
| A_northern_shoveler_Interior_Alaska_4_2007 | H3N8 | 9/5/07 | CY039823 | North American |
| A_northern_shoveler_Interior_Alaska_6MP1283_2006 | H3N6 | 9/2/06 | CY079723 | North American |
| A_northern_shoveler_Interior_Alaska_6MP1287_2006 | H3N8 | 9/2/06 | CY078522 | North American |
| A_northern_shoveler_Interior_Alaska_6MP1339_2006 | H3N8 | 9/9/06 | CY078562 | North American |
| A_northern_shoveler_Interior_Alaska_7MP0944_2007 | H3N8 | 9/7/07 | CY077285 | North American |
| A_northern_shoveler_Interior_Alaska_7MP0953_2007 | H3N8 | 9/7/07 | CY078970 | North American |
| A_northern_shoveler_Interior_Alaska_7MP1033_2007 | H3N8 | 9/12/07 | CY077395 | North American |
| A_northern_shoveler_Interior_Alaska_7MP1077_2007 | H1N1 | 9/10/07 | CY077301 | North American |
| A_northern_shoveler_Interior_Alaska_7MP1080_2007 | H3N8 | 9/10/07 | CY077309 | North American |
| A_northern_shoveler_Interior_Alaska_7MP1081_2007 | H1N1 | 9/10/07 | CY077229 | North American |
| A_northern_shoveler_Interior_Alaska_7MP1649_2007 | H3N8 | 9/2/07 | CY077340 | North American |
| A_northern_shoveler_Interior_Alaska_7MP1670_2007 | H3N8 | 9/2/07 | CY077355 | North American |
| A_northern_shoveler_Interior_Alaska_7MP1765_2007 | H3N8 | 9/4/07 | CY077277 | North American |
| A_northern_shoveler_Interior_Alaska_7MP1766_2007 | H3N8 | 9/4/07 | CY080206 | North American |
| A_northern_shoveler_Interior_Alaska_8BM3470_2008 | H9N2 | 9/1/08 | CY079653 | North American |
| A_northern_shoveler_Interior_Alaska_9BM12298R0_2009 | H3N8 | 9/1/09 | CY143161 | North American |
| A_northern_shoveler_Interior_Alaska_9BM12314R0_2009 | H4N6 | 9/2/09 | CY143169 | North American |
| A_northern_shoveler_Interior_Alaska_9BM12332R0_2009 | H3N8 | 9/2/09 | CY143193 | North American |
| A_northern_shoveler_Interior_Alaska_9BM12519R0_2009 | H4N8 | 9/3/09 | CY143233 | North American |
| A_northern_shoveler_Interior_Alaska_9BM1447R2_2009 | H8N4 | 6/20/09 | CY130331 | North American |
| A_northern_shoveler_Interior_Alaska_9BM2925R0_2009 | H8N4 | 7/16/09 | CY135326 | North American |
| A_northern_shoveler_Interior_Alaska_9BM2943R1_2009 | H4N6 | 7/16/09 | CY135334 | North American |
| A_northern_shoveler_Interior_Alaska_9BM4787R0_2009 | H8N4 | 7/29/09 | CY135502 | North American |
| A_northern_shoveler_Interior_Alaska_9BM4905R0_2009 | H4N6 | 7/29/09 | CY135510 | North American |
| A_northern_shoveler_Minnesota_Sg-00612_2008 | H10N7 | 7/29/08 | CY145484 | North American |
| A_northern_shoveler_Minnesota_Sg-00645_2008 | H3N8 | 7/31/08 | CY042735 | North American |
| A_northern_shoveler_Minnesota_Sg-00645_2008 | H3N8 | 7/31/08 | CY140060 | North American |
| A_northern_shoveler_Minnesota_Sg-00648_2008 | H8N4 | 8/1/08 | CY042745 | North American |
| A_northern_shoveler_Minnesota_Sg-00648_2008 | H8N4 | 8/1/08 | CY140292 | North American |
| A_northern_shoveler_Mississippi_09OS025_2009 | H12N5 | 1/9/09 | CY079395 | North American |
| A_northern_shoveler_Mississippi_09OS168_2009 | H10N6 | 1/11/09 | CY079403 | North American |
| A_northern_shoveler_Mississippi_09OS643_2009 | H7N7 | 1/15/09 | CY079419 | North American |
| A_northern_shoveler_Mississippi_10OS4526_2010 | H6N2 | 12/10/10 | CY133313 | North American |
| A_northern_shoveler_Mississippi_236_2010 | H10N7 | 1/12/10 | CY097150 | North American |
| A_northern_shoveler_Mississippi_252_2010 | H10N7 | 1/10/10 | CY097677 | North American |
| A_northern_shoveler_Mississippi_397_2010 | H1N3 | 1/17/10 | CY097741 | North American |
| A_northern_shoveler_Missouri_10MO0294_2010 | H11N9 | 11/16/10 | CY133824 | North American |
| A_northern_shoveler_Missouri_10OS4632_2010 | H7N7 | 12/12/10 | CY133372 | North American |
| A_northern_shoveler_Missouri_10OS4673_2010 | H14N6 | 12/12/10 | CY133388 | North American |
| A_northern_shoveler_Missouri_10OS4718_2010 | H10N7 | 12/12/10 | CY133407 | North American |
| A_northern_shoveler_Missouri_10OS4750_2010 | H7N3 | 12/12/10 | CY133423 | North American |
| A_northern_shoveler_Missouri_196_2009 | H10N3 | 12/7/09 | CY097621 | North American |
| A_northern_shoveler_Missouri_298_2009 | H9N2 | 12/6/09 | CY097637 | North American |
| A_northern_shoveler_Oregon_44336-179_2007 | H4N6 | 9/10/07 | CY076476 | North American |
| A_northern_shoveler_Washington_44249-603_2006 | H6N1 | 10/26/06 | CY076252 | North American |
| A_northern_shoveler_Washington_44249-645_2006 | H5N2 | 11/4/06 | CY076260 | North American |
| A_northern_shoveler_Washington_44249-664_2006 | H7N3 | 11/7/06 | CY076268 | North American |
| A_northern_shoveler_Washington_44249-675_2006 | H10N2 | 11/7/06 | CY076276 | North American |
| A_northern_shoveler_Washington_44249-700_2006 | H10N1 | 11/11/06 | CY076284 | North American |
| A_northern_shoveler_Washington_44249-731_2006 | H10N7 | 11/30/06 | CY076292 | North American |
| A_northern_shoveler_Washington_44249-749_2006 | H7N3 | 12/7/06 | CY076300 | North American |
| A_northern_shoveler_Washington_44249-752_2006 | H7N3 | 12/7/06 | CY076308 | North American |
| A_northern_shoveler_Washington_44249-765_2006 | H10N2 | 12/9/06 | CY076316 | North American |
| A_northern_shoveler_Washington_44249-783_2006 | H7N3 | 12/14/06 | CY076324 | North American |
| A_northern_shoveler_Wisconsin_10OS3226_2010 | H7N3 | 11/10/10 | CY133184 | North American |
| A_northern_shoveler_Wisconsin_2508_2009 | H4N2 | 10/3/09 | CY097437 | North American |
| A_pekin_duck_California_P30_2006 | H4N2 | 3/6/06 | CY053828 | North American |
| A_pintail_Alberta_21_2006 | H1N1 | 7/24/06 | CY045398 | North American |
| A_red_knot_New_Jersey_AI06-096_2006 | H7N3 | 5/17/06 | CY144682 | North American |
| A_red_knot_New_Jersey_Sg-00478_2008 | H12N5 | 5/16/08 | CY038053 | North American |
| A_red_knot_New_Jersey_Sg-00478_2008 | H12N5 | 5/16/08 | CY144956 | North American |
| A_red_knot_New_Jersey_Sg-00479_2008 | H4N6 | 5/16/08 | CY038058 | North American |
| A_red_knot_New_Jersey_Sg-00479_2008 | H4N6 | 5/16/08 | CY144964 | North American |
| A_red_knot_New_Jersey_Sg-00483_2008 | H12N5 | 5/20/08 | CY038073 | North American |
| A_red_knot_New_Jersey_Sg-00483_2008 | H12N5 | 5/20/08 | CY145548 | North American |
| A_red-necked_grebe_Minnesota_AI10-1948_2010 | H1N1 | 7/7/10 | CY141088 | North American |
| A_red-necked_grebe_Minnesota_AI10-1954_2010 | H1N1 | 7/7/10 | CY141096 | North American |
| A_red-necked_grebe_Minnesota_AI10-2528_2010 | H4N8 | 8/7/10 | CY141112 | North American |
| A_red-necked_stint_Australia_4_2004 | H4N8 | 2004 | CY039262 | North American |
| A_redhead_Alberta_458_2007 | H3N8 | 8/28/07 | CY103458 | North American |
| A_ring_necked_duck_California_HKWF402_2007 | H6N1 | 11/3/07 | CY033401 | North American |
| A_ring_necked_duck_California_HKWF515_2007 | H6N1 | 11/7/07 | CY033425 | North American |
| A_ring-billed_gull_Quebec_02434-1_2009 | H13N6 | 7/20/09 | CY125219 | North American |
| A_ring-billed_gull_Quebec_G018_2010 | H1N3 | 5/12/10 | CY138104 | North American |
| A_ring-billed_gull_Quebec_G066_2010 | H1N3 | 5/12/10 | CY138112 | North American |
| A_ring-billed_gull_Quebec_G068_2010 | H1N3 | 5/12/10 | CY138120 | North American |
| A_ring-billed_gull_Quebec_G139_2010 | H1N3 | 5/18/10 | CY138128 | North American |
| A_ring-billed_gull_Quebec_G192_2010 | H1N3 | 5/19/10 | CY138136 | North American |
| A_ring-necked_duck_California_HKWF662_2007 | H6N1 | 11/11/07 | CY032921 | North American |
| A_ring-necked_duck_Illinois_4125_2009 | H4N8 | 11/29/09 | CY097557 | North American |
| A_ring-necked_duck_Interior_Alaska_10BM05617R0_2010 | H4N6 | 7/26/10 | CY135786 | North American |
| A_ring-necked_duck_Interior_Alaska_10BM05792R0_2010 | H3N8 | 7/26/10 | CY135810 | North American |
| A_ring-necked_duck_Minnesota_Sg-00067_2007 | H4N6 | 8/3/07 | CY064168 | North American |
| A_ring-necked_duck_Minnesota_Sg-00068_2007 | H10N7 | 8/3/07 | CY064176 | North American |
| A_ring-necked_duck_Minnesota_Sg-01066_2008 | H12N8 | 10/12/08 | CY140622 | North American |
| A_ring-necked_duck_New_Brunswick_03400_2009 | H3N8 | 8/9/09 | CY125396 | North American |
| A_ring-necked_duck_New_Brunswick_03449_2009 | H11N9 | 8/12/09 | CY125356 | North American |
| A_ring-necked_duck_Nova_Scotia_03378_2009 | H3N8 | 8/8/09 | CY125340 | North American |
| A_rock_dove_Oregon_20547-003_2007 | H1N2 | 11/5/07 | CY076148 | North American |
| A_rock_dove_Oregon_20547-004_2007 | H1N2 | 11/5/07 | CY076156 | North American |
| A_ruddy_duck_Illinois_3471_2009 | H3N8 | 11/1/09 | CY097065 | North American |
| A_ruddy_turnstone_Delaware_103_2007 | H5N1 | 5/22/07 | CY077117 | North American |
| A_ruddy_turnstone_Delaware_291_2006 | H6N1 | 5/22/06 | CY047026 | North American |
| A_ruddy_turnstone_Delaware_293_2006 | H6N2 | 5/22/06 | CY077133 | North American |
| A_ruddy_turnstone_Delaware_AI06-778_2006 | H7N3 | 5/22/06 | CY144778 | North American |
| A_ruddy_turnstone_Delaware_AI09-1221_2009 | H1N1 | 6/2/09 | CY146326 | North American |
| A_ruddy_turnstone_Delaware_AI09-1268_2009 | H10N7 | 6/2/09 | CY146367 | North American |
| A_ruddy_turnstone_Delaware_AI09-467_2009 | H10N7 | 5/28/09 | CY146027 | North American |
| A_ruddy_turnstone_Delaware_AI09-666_2009 | H6N1 | 5/28/09 | CY146116 | North American |
| A_ruddy_turnstone_Delaware_AI09-769_2009 | H1N7 | 5/28/09 | CY146140 | North American |
| A_ruddy_turnstone_Delaware_AI09-879_2009 | H10N7 | 5/28/09 | CY146196 | North American |
| A_ruddy_turnstone_Delaware_AI09-960_2009 | H1N7 | 5/28/09 | CY146237 | North American |
| A_ruddy_turnstone_Delaware_Bay_108_2007 | H7N3 | 5/22/07 | CY036782 | North American |
| A_ruddy_turnstone_Delaware_Bay_121_2007 | H7N3 | 5/22/07 | CY036790 | North American |
| A_ruddy_turnstone_Delaware_Bay_123_2007 | H7N3 | 5/22/07 | CY036798 | North American |
| A_ruddy_turnstone_Delaware_Bay_124_2007 | H7N3 | 5/22/07 | CY039366 | North American |
| A_ruddy_turnstone_Delaware_Bay_124_2007 | H7N3 | 5/22/07 | CY103185 | North American |
| A_ruddy_turnstone_Delaware_Bay_136_2007 | H6N1 | 5/22/07 | CY127790 | North American |
| A_ruddy_turnstone_Delaware_Bay_173_2007 | H6N2 | 5/23/07 | CY127798 | North American |
| A_ruddy_turnstone_Delaware_Bay_262_2006 | H7N3 | 5/22/06 | CY039334 | North American |
| A_ruddy_turnstone_Delaware_Bay_279_2006 | H7N3 | 5/22/06 | CY041321 | North American |
| A_ruddy_turnstone_Delaware_Bay_281_2006 | H7N3 | 5/22/06 | CY102982 | North American |
| A_ruddy_turnstone_Delaware_Bay_282_2006 | H7N3 | 5/22/06 | CY102990 | North American |
| A_ruddy_turnstone_Delaware_Bay_283_2006 | H7N3 | 5/22/06 | CY037110 | North American |
| A_ruddy_turnstone_Delaware_Sg-00468_2008 | H10N7 | 5/21/08 | CY042404 | North American |
| A_ruddy_turnstone_Delaware_Sg-00468_2008 | H10N7 | 5/21/08 | CY144916 | North American |
| A_ruddy_turnstone_Delaware_Sg-00469_2008 | H3N2 | 5/21/08 | CY038028 | North American |
| A_ruddy_turnstone_Delaware_Sg-00469_2008 | H3N2 | 5/21/08 | CY145500 | North American |
| A_ruddy_turnstone_Delaware_Sg-00470_2008 | H4N6 | 5/21/08 | CY038033 | North American |
| A_ruddy_turnstone_Delaware_Sg-00470_2008 | H4N6 | 5/21/08 | CY144924 | North American |
| A_ruddy_turnstone_Delaware_Sg-00472_2008 | H10N7 | 5/21/08 | CY042412 | North American |
| A_ruddy_turnstone_Delaware_Sg-00472_2008 | H10N7 | 5/21/08 | CY144932 | North American |
| A_ruddy_turnstone_Delaware_Sg-00473_2008 | H4N6 | 5/21/08 | CY145516 | North American |
| A_ruddy_turnstone_Delaware_Sg-00474_2008 | H10N7 | 5/21/08 | CY145524 | North American |
| A_ruddy_turnstone_Delaware_Sg-00538_2008 | H1N5 | 5/31/08 | CY042540 | North American |
| A_ruddy_turnstone_Delaware_Sg-00538_2008 | H1N5 | 5/31/08 | CY145328 | North American |
| A_ruddy_turnstone_Delaware_Sg-00539_2008 | H4N6 | 5/31/08 | CY038193 | North American |
| A_ruddy_turnstone_Delaware_Sg-00539_2008 | H4N6 | 5/31/08 | CY145336 | North American |
| A_ruddy_turnstone_Delaware_Sg-00540_2008 | H12N4 | 5/31/08 | CY145654 | North American |
| A_ruddy_turnstone_Delaware_Sg-00541_2008 | H4N6 | 5/31/08 | CY042546 | North American |
| A_ruddy_turnstone_Delaware_Sg-00541_2008 | H4N6 | 5/31/08 | CY145344 | North American |
| A_ruddy_turnstone_New_Jersey_AI06-179_2006 | H7N3 | 5/17/06 | CY144698 | North American |
| A_ruddy_turnstone_New_Jersey_AI06-539_2006 | H9N2 | 5/24/06 | CY144706 | North American |
| A_ruddy_turnstone_New_Jersey_AI06-582_2006 | H6N7 | 5/24/06 | CY144674 | North American |
| A_ruddy_turnstone_New_Jersey_AI07-296_2007 | H6N1 | 7/27/07 | CY144762 | North American |
| A_ruddy_turnstone_New_Jersey_AI07-69_2007 | H5N1 | 7/26/07 | CY144746 | North American |
| A_ruddy_turnstone_New_Jersey_AI07-697_2007 | H12N5 | 8/10/07 | CY144722 | North American |
| A_ruddy_turnstone_New_Jersey_AI07-699_2007 | H5N1 | 8/10/07 | CY144754 | North American |
| A_ruddy_turnstone_New_Jersey_AI07-72_2007 | H9N1 | 3/8/07 | CY144770 | North American |
| A_ruddy_turnstone_New_Jersey_AI07-796_2007 | H12N5 | 8/17/07 | CY144730 | North American |
| A_ruddy_turnstone_New_Jersey_AI07-803_2007 | H12N5 | 8/16/07 | CY144738 | North American |
| A_ruddy_turnstone_New_Jersey_AI07-839_2007 | H12N1 | 8/16/07 | CY144714 | North American |
| A_ruddy_turnstone_New_Jersey_AI09-027_2009 | H11N1 | 5/13/09 | CY145865 | North American |
| A_ruddy_turnstone_New_Jersey_AI09-1008_2009 | H11N9 | 6/3/09 | CY146261 | North American |
| A_ruddy_turnstone_New_Jersey_AI09-1033_2009 | H10N7 | 5/30/09 | CY146269 | North American |
| A_ruddy_turnstone_New_Jersey_AI09-1082_2009 | H1N9 | 5/30/09 | CY146286 | North American |
| A_ruddy_turnstone_New_Jersey_AI09-1144_2009 | H11N9 | 5/30/09 | CY146294 | North American |
| A_ruddy_turnstone_New_Jersey_AI09-1149_2009 | H10N7 | 6/3/09 | CY146302 | North American |
| A_ruddy_turnstone_New_Jersey_AI09-1164_2009 | H11N9 | 5/30/09 | CY146310 | North American |
| A_ruddy_turnstone_New_Jersey_AI09-119_2009 | H1N8 | 5/20/09 | CY145882 | North American |
| A_ruddy_turnstone_New_Jersey_AI09-1211_2009 | H1N9 | 6/3/09 | CY146318 | North American |
| A_ruddy_turnstone_New_Jersey_AI09-1228_2009 | H10N7 | 6/3/09 | CY146334 | North American |
| A_ruddy_turnstone_New_Jersey_AI09-1233_2009 | H1N7 | 6/3/09 | CY146342 | North American |
| A_ruddy_turnstone_New_Jersey_AI09-1251_2009 | H10N7 | 6/3/09 | CY146359 | North American |
| A_ruddy_turnstone_New_Jersey_AI09-1292_2009 | H1N1 | 6/3/09 | CY146383 | North American |
| A_ruddy_turnstone_New_Jersey_AI09-1299_2009 | H1N1 | 5/30/09 | CY146391 | North American |
| A_ruddy_turnstone_New_Jersey_AI09-1325_2009 | H10N7 | 5/30/09 | CY146399 | North American |
| A_ruddy_turnstone_New_Jersey_AI09-1326_2009 | H10N7 | 5/30/09 | CY146407 | North American |
| A_ruddy_turnstone_New_Jersey_AI09-1336_2009 | H10N7 | 5/30/09 | CY146415 | North American |
| A_ruddy_turnstone_New_Jersey_AI09-1352_2009 | H10N7 | 5/30/09 | CY146423 | North American |
| A_ruddy_turnstone_New_Jersey_AI09-1381_2009 | H1N9 | 5/30/09 | CY146431 | North American |
| A_ruddy_turnstone_New_Jersey_AI09-151_2009 | H11N1 | 5/11/09 | CY145890 | North American |
| A_ruddy_turnstone_New_Jersey_AI09-169_2009 | H11N8 | 5/8/09 | CY145906 | North American |
| A_ruddy_turnstone_New_Jersey_AI09-194_2009 | H11N8 | 5/13/09 | CY145914 | North American |
| A_ruddy_turnstone_New_Jersey_AI09-256_2009 | H1N1 | 5/14/09 | CY145930 | North American |
| A_ruddy_turnstone_New_Jersey_AI09-294_2009 | H13N6 | 5/11/09 | CY145946 | North American |
| A_ruddy_turnstone_New_Jersey_AI09-305_2009 | H10N7 | 5/11/09 | CY145954 | North American |
| A_ruddy_turnstone_New_Jersey_AI09-342_2009 | H10N7 | 5/20/09 | CY145970 | North American |
| A_ruddy_turnstone_New_Jersey_AI09-372_2009 | H6N1 | 5/8/09 | CY145978 | North American |
| A_ruddy_turnstone_New_Jersey_AI09-377_2009 | H6N1 | 5/14/09 | CY145986 | North American |
| A_ruddy_turnstone_New_Jersey_AI09-464_2009 | H1N7 | 5/26/09 | CY146019 | North American |
| A_ruddy_turnstone_New_Jersey_AI09-474_2009 | H10N1 | 5/29/09 | CY146035 | North American |
| A_ruddy_turnstone_New_Jersey_AI09-525_2009 | H1N7 | 5/26/09 | CY146043 | North American |
| A_ruddy_turnstone_New_Jersey_AI09-549_2009 | H10N7 | 5/26/09 | CY146051 | North American |
| A_ruddy_turnstone_New_Jersey_AI09-554_2009 | H10N7 | 5/26/09 | CY146059 | North American |
| A_ruddy_turnstone_New_Jersey_AI09-573_2009 | H1N1 | 5/29/09 | CY146067 | North American |
| A_ruddy_turnstone_New_Jersey_AI09-587_2009 | H10N7 | 5/24/09 | CY146075 | North American |
| A_ruddy_turnstone_New_Jersey_AI09-618_2009 | H1N9 | 5/26/09 | CY146092 | North American |
| A_ruddy_turnstone_New_Jersey_AI09-620_2009 | H11N1 | 5/22/09 | CY146100 | North American |
| A_ruddy_turnstone_New_Jersey_AI09-626_2009 | H1N1 | 5/26/09 | CY146108 | North American |
| A_ruddy_turnstone_New_Jersey_AI09-710_2009 | H1N8 | 5/22/09 | CY146124 | North American |
| A_ruddy_turnstone_New_Jersey_AI09-727_2009 | H1N9 | 5/26/09 | CY146132 | North American |
| A_ruddy_turnstone_New_Jersey_AI09-775_2009 | H1N1 | 5/29/09 | CY146148 | North American |
| A_ruddy_turnstone_New_Jersey_AI09-827_2009 | H10N1 | 5/29/09 | CY146156 | North American |
| A_ruddy_turnstone_New_Jersey_AI09-837_2009 | H1N9 | 5/29/09 | CY146164 | North American |
| A_ruddy_turnstone_New_Jersey_AI09-841_2009 | H1N1 | 5/22/09 | CY146172 | North American |
| A_ruddy_turnstone_New_Jersey_AI09-846_2009 | H1N1 | 5/26/09 | CY146180 | North American |
| A_ruddy_turnstone_New_Jersey_AI09-874_2009 | H1N8 | 5/22/09 | CY146188 | North American |
| A_ruddy_turnstone_New_Jersey_AI09-907_2009 | H10N7 | 5/29/09 | CY146204 | North American |
| A_ruddy_turnstone_New_Jersey_AI09-937_2009 | H6N1 | 5/26/09 | CY146221 | North American |
| A_ruddy_turnstone_New_Jersey_AI09-944_2009 | H10N7 | 5/22/09 | CY146229 | North American |
| A_ruddy_turnstone_New_Jersey_AI09-961_2009 | H10N7 | 5/22/09 | CY146245 | North American |
| A_ruddy_turnstone_New_Jersey_Sg-00471_2008 | H6N8 | 5/13/08 | CY145508 | North American |
| A_ruddy_turnstone_New_Jersey_Sg-00475_2008 | H4N6 | 5/17/08 | CY042421 | North American |
| A_ruddy_turnstone_New_Jersey_Sg-00475_2008 | H4N6 | 5/17/08 | CY145532 | North American |
| A_ruddy_turnstone_New_Jersey_Sg-00476_2008 | H12N5 | 5/16/08 | CY038043 | North American |
| A_ruddy_turnstone_New_Jersey_Sg-00476_2008 | H12N5 | 5/16/08 | CY144940 | North American |
| A_ruddy_turnstone_New_Jersey_Sg-00477_2008 | H4N6 | 5/17/08 | CY038048 | North American |
| A_ruddy_turnstone_New_Jersey_Sg-00477_2008 | H4N6 | 5/17/08 | CY144948 | North American |
| A_ruddy_turnstone_New_Jersey_Sg-00480_2008 | H4N6 | 5/18/08 | CY038063 | North American |
| A_ruddy_turnstone_New_Jersey_Sg-00480_2008 | H4N6 | 5/18/08 | CY144972 | North American |
| A_ruddy_turnstone_New_Jersey_Sg-00481_2008 | H12N5 | 5/18/08 | CY038068 | North American |
| A_ruddy_turnstone_New_Jersey_Sg-00481_2008 | H12N5 | 5/18/08 | CY145540 | North American |
| A_ruddy_turnstone_New_Jersey_Sg-00482_2008 | H6N8 | 5/10/08 | CY144980 | North American |
| A_ruddy_turnstone_New_Jersey_Sg-00484_2008 | H12N5 | 5/16/08 | CY038078 | North American |
| A_ruddy_turnstone_New_Jersey_Sg-00484_2008 | H12N5 | 5/16/08 | CY145556 | North American |
| A_ruddy_turnstone_New_Jersey_Sg-00487_2008 | H10N7 | 5/18/08 | CY042435 | North American |
| A_ruddy_turnstone_New_Jersey_Sg-00487_2008 | H10N7 | 5/18/08 | CY145004 | North American |
| A_ruddy_turnstone_New_Jersey_Sg-00488_2008 | H12N5 | 5/18/08 | CY038083 | North American |
| A_ruddy_turnstone_New_Jersey_Sg-00488_2008 | H12N5 | 5/18/08 | CY145012 | North American |
| A_ruddy_turnstone_New_Jersey_Sg-00489_2008 | H4N6 | 5/18/08 | CY038088 | North American |
| A_ruddy_turnstone_New_Jersey_Sg-00489_2008 | H4N6 | 5/18/08 | CY145020 | North American |
| A_ruddy_turnstone_New_Jersey_Sg-00490_2008 | H10N7 | 5/16/08 | CY038093 | North American |
| A_ruddy_turnstone_New_Jersey_Sg-00490_2008 | H10N7 | 5/16/08 | CY145028 | North American |
| A_ruddy_turnstone_New_Jersey_Sg-00492_2008 | H4N6 | 5/18/08 | CY042440 | North American |
| A_ruddy_turnstone_New_Jersey_Sg-00492_2008 | H4N6 | 5/18/08 | CY145045 | North American |
| A_ruddy_turnstone_New_Jersey_Sg-00493_2008 | H12N5 | 5/18/08 | CY038098 | North American |
| A_ruddy_turnstone_New_Jersey_Sg-00493_2008 | H12N5 | 5/18/08 | CY145053 | North American |
| A_ruddy_turnstone_New_Jersey_Sg-00494_2008 | H4N6 | 5/18/08 | CY038103 | North American |
| A_ruddy_turnstone_New_Jersey_Sg-00494_2008 | H4N6 | 5/18/08 | CY145061 | North American |
| A_ruddy_turnstone_New_Jersey_Sg-00495_2008 | H12N5 | 5/17/08 | CY038108 | North American |
| A_ruddy_turnstone_New_Jersey_Sg-00495_2008 | H12N5 | 5/17/08 | CY145564 | North American |
| A_ruddy_turnstone_New_Jersey_Sg-00496_2008 | H4N6 | 5/10/08 | CY038113 | North American |
| A_ruddy_turnstone_New_Jersey_Sg-00496_2008 | H4N6 | 5/10/08 | CY145069 | North American |
| A_ruddy_turnstone_New_Jersey_Sg-00497_2008 | H12N5 | 5/17/08 | CY038118 | North American |
| A_ruddy_turnstone_New_Jersey_Sg-00497_2008 | H12N5 | 5/17/08 | CY145077 | North American |
| A_ruddy_turnstone_New_Jersey_Sg-00498_2008 | H10N7 | 5/17/08 | CY042444 | North American |
| A_ruddy_turnstone_New_Jersey_Sg-00498_2008 | H10N7 | 5/17/08 | CY145572 | North American |
| A_ruddy_turnstone_New_Jersey_Sg-00500_2008 | H12N5 | 5/16/08 | CY038123 | North American |
| A_ruddy_turnstone_New_Jersey_Sg-00500_2008 | H12N5 | 5/16/08 | CY145590 | North American |
| A_ruddy_turnstone_New_Jersey_Sg-00501_2008 | H12N5 | 5/16/08 | CY038128 | North American |
| A_ruddy_turnstone_New_Jersey_Sg-00501_2008 | H12N5 | 5/16/08 | CY145085 | North American |
| A_ruddy_turnstone_New_Jersey_Sg-00502_2008 | H4N6 | 5/17/08 | CY042452 | North American |
| A_ruddy_turnstone_New_Jersey_Sg-00502_2008 | H4N6 | 5/17/08 | CY145598 | North American |
| A_ruddy_turnstone_New_Jersey_Sg-00504_2008 | H10N7 | 5/18/08 | CY042459 | North American |
| A_ruddy_turnstone_New_Jersey_Sg-00504_2008 | H10N7 | 5/18/08 | CY145103 | North American |
| A_ruddy_turnstone_New_Jersey_Sg-00506_2008 | H4N6 | 5/17/08 | CY038138 | North American |
| A_ruddy_turnstone_New_Jersey_Sg-00506_2008 | H4N6 | 5/17/08 | CY145111 | North American |
| A_ruddy_turnstone_New_Jersey_Sg-00507_2008 | H4N6 | 5/16/08 | CY038143 | North American |
| A_ruddy_turnstone_New_Jersey_Sg-00507_2008 | H4N6 | 5/16/08 | CY145119 | North American |
| A_ruddy_turnstone_New_Jersey_Sg-00508_2008 | H10N7 | 5/23/08 | CY042463 | North American |
| A_ruddy_turnstone_New_Jersey_Sg-00508_2008 | H10N7 | 5/23/08 | CY145614 | North American |
| A_ruddy_turnstone_New_Jersey_Sg-00509_2008 | H12N5 | 5/22/08 | CY038148 | North American |
| A_ruddy_turnstone_New_Jersey_Sg-00509_2008 | H12N5 | 5/22/08 | CY145127 | North American |
| A_ruddy_turnstone_New_Jersey_Sg-00510_2008 | H4N6 | 5/23/08 | CY042466 | North American |
| A_ruddy_turnstone_New_Jersey_Sg-00510_2008 | H4N6 | 5/23/08 | CY145135 | North American |
| A_ruddy_turnstone_New_Jersey_Sg-00511_2008 | H10N7 | 5/22/08 | CY042470 | North American |
| A_ruddy_turnstone_New_Jersey_Sg-00511_2008 | H10N7 | 5/22/08 | CY145143 | North American |
| A_ruddy_turnstone_New_Jersey_Sg-00512_2008 | H10N7 | 5/23/08 | CY042474 | North American |
| A_ruddy_turnstone_New_Jersey_Sg-00512_2008 | H10N7 | 5/23/08 | CY145151 | North American |
| A_ruddy_turnstone_New_Jersey_Sg-00513_2008 | H3N2 | 5/28/08 | CY038153 | North American |
| A_ruddy_turnstone_New_Jersey_Sg-00513_2008 | H3N2 | 5/28/08 | CY145159 | North American |
| A_ruddy_turnstone_New_Jersey_Sg-00514_2008 | H10N7 | 5/23/08 | CY042478 | North American |
| A_ruddy_turnstone_New_Jersey_Sg-00514_2008 | H10N7 | 5/23/08 | CY145167 | North American |
| A_ruddy_turnstone_New_Jersey_Sg-00516_2008 | H10N7 | 5/28/08 | CY042485 | North American |
| A_ruddy_turnstone_New_Jersey_Sg-00516_2008 | H10N7 | 5/28/08 | CY145184 | North American |
| A_ruddy_turnstone_New_Jersey_Sg-00517_2008 | H10N7 | 5/22/08 | CY042489 | North American |
| A_ruddy_turnstone_New_Jersey_Sg-00517_2008 | H10N7 | 5/22/08 | CY145622 | North American |
| A_ruddy_turnstone_New_Jersey_Sg-00518_2008 | H3N2 | 5/28/08 | CY038158 | North American |
| A_ruddy_turnstone_New_Jersey_Sg-00518_2008 | H3N2 | 5/28/08 | CY145630 | North American |
| A_ruddy_turnstone_New_Jersey_Sg-00519_2008 | H3N2 | 5/28/08 | CY145192 | North American |
| A_ruddy_turnstone_New_Jersey_Sg-00520_2008 | H3N2 | 5/23/08 | CY145200 | North American |
| A_ruddy_turnstone_New_Jersey_Sg-00521_2008 | H10N7 | 5/22/08 | CY145208 | North American |
| A_ruddy_turnstone_New_Jersey_Sg-00522_2008 | H3N7 | 5/22/08 | CY038163 | North American |
| A_ruddy_turnstone_New_Jersey_Sg-00522_2008 | H3N7 | 5/22/08 | CY145216 | North American |
| A_ruddy_turnstone_New_Jersey_Sg-00523_2008 | H3N2 | 5/28/08 | CY042500 | North American |
| A_ruddy_turnstone_New_Jersey_Sg-00523_2008 | H3N2 | 5/28/08 | CY145224 | North American |
| A_ruddy_turnstone_New_Jersey_Sg-00524_2008 | H4N6 | 5/22/08 | CY042503 | North American |
| A_ruddy_turnstone_New_Jersey_Sg-00524_2008 | H4N6 | 5/22/08 | CY145232 | North American |
| A_ruddy_turnstone_New_Jersey_Sg-00525_2008 | H3N8 | 5/22/08 | CY038168 | North American |
| A_ruddy_turnstone_New_Jersey_Sg-00525_2008 | H3N8 | 5/22/08 | CY145638 | North American |
| A_ruddy_turnstone_New_Jersey_Sg-00526_2008 | H10N7 | 5/23/08 | CY038173 | North American |
| A_ruddy_turnstone_New_Jersey_Sg-00526_2008 | H10N7 | 5/23/08 | CY145240 | North American |
| A_ruddy_turnstone_New_Jersey_Sg-00527_2008 | H4N6 | 5/23/08 | CY038178 | North American |
| A_ruddy_turnstone_New_Jersey_Sg-00527_2008 | H4N6 | 5/23/08 | CY145248 | North American |
| A_ruddy_turnstone_New_Jersey_Sg-00528_2008 | H10N7 | 5/28/08 | CY042508 | North American |
| A_ruddy_turnstone_New_Jersey_Sg-00528_2008 | H10N7 | 5/28/08 | CY145256 | North American |
| A_ruddy_turnstone_New_Jersey_Sg-00529_2008 | H3N2 | 5/28/08 | CY042512 | North American |
| A_ruddy_turnstone_New_Jersey_Sg-00529_2008 | H3N2 | 5/28/08 | CY145264 | North American |
| A_ruddy_turnstone_New_Jersey_Sg-00530_2008 | H12N5 | 5/22/08 | CY042515 | North American |
| A_ruddy_turnstone_New_Jersey_Sg-00530_2008 | H12N5 | 5/22/08 | CY145272 | North American |
| A_ruddy_turnstone_New_Jersey_Sg-00531_2008 | H4N6 | 5/22/08 | CY042519 | North American |
| A_ruddy_turnstone_New_Jersey_Sg-00531_2008 | H4N6 | 5/22/08 | CY145280 | North American |
| A_ruddy_turnstone_New_Jersey_Sg-00532_2008 | H1N5 | 6/4/08 | CY042524 | North American |
| A_ruddy_turnstone_New_Jersey_Sg-00532_2008 | H1N5 | 6/4/08 | CY145646 | North American |
| A_ruddy_turnstone_New_Jersey_Sg-00533_2008 | H4N6 | 5/30/08 | CY038183 | North American |
| A_ruddy_turnstone_New_Jersey_Sg-00533_2008 | H4N6 | 5/30/08 | CY145288 | North American |
| A_ruddy_turnstone_New_Jersey_Sg-00534_2008 | H4N6 | 5/22/08 | CY038188 | North American |
| A_ruddy_turnstone_New_Jersey_Sg-00534_2008 | H4N6 | 5/22/08 | CY145296 | North American |
| A_ruddy_turnstone_New_Jersey_Sg-00535_2008 | H10N7 | 5/22/08 | CY042528 | North American |
| A_ruddy_turnstone_New_Jersey_Sg-00535_2008 | H10N7 | 5/22/08 | CY145304 | North American |
| A_ruddy_turnstone_New_Jersey_Sg-00536_2008 | H10N7 | 5/28/08 | CY042532 | North American |
| A_ruddy_turnstone_New_Jersey_Sg-00536_2008 | H10N7 | 5/28/08 | CY145312 | North American |
| A_ruddy_turnstone_New_Jersey_Sg-00537_2008 | H10N7 | 5/22/08 | CY042536 | North American |
| A_ruddy_turnstone_New_Jersey_Sg-00537_2008 | H10N7 | 5/22/08 | CY145320 | North American |
| A_ruddy_turnstone_New_Jersey_Sg-00542_2008 | H4N6 | 6/1/08 | CY038198 | North American |
| A_ruddy_turnstone_New_Jersey_Sg-00542_2008 | H4N6 | 6/1/08 | CY145352 | North American |
| A_ruddy_turnstone_New_Jersey_Sg-00543_2008 | H3N7 | 6/1/08 | CY042551 | North American |
| A_ruddy_turnstone_New_Jersey_Sg-00543_2008 | H3N7 | 6/1/08 | CY145360 | North American |
| A_ruddy_turnstone_New_Jersey_Sg-00544_2008 | H1N5 | 6/1/08 | CY145368 | North American |
| A_ruddy_turnstone_New_Jersey_Sg-00546_2008 | H4N6 | 6/1/08 | CY145385 | North American |
| A_ruddy_turnstone_New_Jersey_Sg-00548_2008 | H10N2 | 6/1/08 | CY042565 | North American |
| A_ruddy_turnstone_New_Jersey_Sg-00548_2008 | H10N2 | 6/1/08 | CY145662 | North American |
| A_ruddy_turnstone_New_Jersey_Sg-00550_2008 | H4N6 | 5/28/08 | CY038203 | North American |
| A_ruddy_turnstone_New_Jersey_Sg-00550_2008 | H4N6 | 5/28/08 | CY145411 | North American |
| A_ruddy_turnstone_New_Jersey_Sg-00551_2008 | H6N6 | 5/22/08 | CY038208 | North American |
| A_ruddy_turnstone_New_Jersey_Sg-00551_2008 | H6N6 | 5/22/08 | CY145670 | North American |
| A_ruddy_turnstone_New_Jersey_Sg-00552_2008 | H4N6 | 6/4/08 | CY038213 | North American |
| A_ruddy_turnstone_New_Jersey_Sg-00552_2008 | H4N6 | 6/4/08 | CY145419 | North American |
| A_ruddy_turnstone_New_Jersey_Sg-00554_2008 | H10N7 | 5/22/08 | CY042576 | North American |
| A_ruddy_turnstone_New_Jersey_Sg-00554_2008 | H10N7 | 5/22/08 | CY145436 | North American |
| A_ruddy_turnstone_New_Jersey_Sg-00555_2008 | H3N2 | 5/22/08 | CY145678 | North American |
| A_ruddy_turnstone_New_Jersey_Sg-00556_2008 | H4N6 | 6/4/08 | CY038218 | North American |
| A_ruddy_turnstone_New_Jersey_Sg-00556_2008 | H4N6 | 6/4/08 | CY145686 | North American |
| A_ruddy_turnstone_New_Jersey_Sg-00557_2008 | H11N5 | 6/4/08 | CY042585 | North American |
| A_ruddy_turnstone_New_Jersey_Sg-00557_2008 | H11N5 | 6/4/08 | CY145444 | North American |
| A_ruddy_turnstone_New_Jersey_Sg-00558_2008 | H4N6 | 6/4/08 | CY038223 | North American |
| A_ruddy_turnstone_New_Jersey_Sg-00558_2008 | H4N6 | 6/4/08 | CY145452 | North American |
| A_ruddy_turnstone_New_Jersey_Sg-00560_2008 | H12N5 | 5/16/08 | CY038228 | North American |
| A_ruddy_turnstone_New_Jersey_Sg-00560_2008 | H12N5 | 5/16/08 | CY145468 | North American |
| A_ruddy_turnstone_New_Jersey_Sg-00561_2008 | H11N9 | 5/18/08 | CY038233 | North American |
| A_ruddy_turnstone_New_Jersey_Sg-00561_2008 | H11N9 | 5/18/08 | CY145694 | North American |
| A_ruddy_turnstone_New_Jersey_Sg-00562_2008 | H4N6 | 5/18/08 | CY042595 | North American |
| A_ruddy_turnstone_New_Jersey_Sg-00562_2008 | H4N6 | 5/18/08 | CY145702 | North American |
| A_ruddy_turnstone_New_Jersey_Sg-00563_2008 | H10N7 | 5/10/08 | CY038238 | North American |
| A_ruddy_turnstone_New_Jersey_Sg-00563_2008 | H10N7 | 5/10/08 | CY145710 | North American |
| A_ruddy_turnstone_New_Jersey_Sg-00564_2008 | H11N9 | 5/22/08 | CY038243 | North American |
| A_ruddy_turnstone_New_Jersey_Sg-00564_2008 | H11N9 | 5/22/08 | CY145718 | North American |
| A_ruddy_turnstone_New_Jersey_Sg-00565_2008 | H4N6 | 6/4/08 | CY038248 | North American |
| A_ruddy_turnstone_New_Jersey_Sg-00565_2008 | H4N6 | 6/4/08 | CY145476 | North American |
| A_ruddy_turnstone_New_Jersey_Sg-00567_2008 | H11N2 | 6/4/08 | CY145726 | North American |
| A_ruddy_turnstone_New_Jersey_Sg-0505_2008 | H12N5 | 5/18/08 | CY038133 | North American |
| A_ruddy_turnstone_New_Jersey_Sg-0505_2008 | H12N5 | 5/18/08 | CY145606 | North American |
| A_sanderling_Delaware_Bay_449_2006 | H9N2 | 5/23/06 | CY077149 | North American |
| A_sanderling_Delaware_Bay_449_2006 | H9N2 | 5/23/06 | CY103006 | North American |
| A_sanderling_New_Jersey_AI06-125_2006 | H7N3 | 5/17/06 | CY144690 | North American |
| A_sanderling_New_Jersey_Sg-00486_2008 | H10N7 | 5/18/08 | CY042431 | North American |
| A_sanderling_New_Jersey_Sg-00486_2008 | H10N7 | 5/18/08 | CY144996 | North American |
| A_shorebird_Delaware_133_2006 | H6N8 | 5/22/06 | CY041289 | North American |
| A_shorebird_Delaware_189_2007 | H5N1 | 5/23/07 | CY045406 | North American |
| A_shorebird_Delaware_195_2006 | H16N3 | 5/22/06 | CY045390 | North American |
| A_shorebird_Delaware_221_2006 | H13N9 | 5/22/06 | CY043895 | North American |
| A_shorebird_Delaware_224_2006 | H13N9 | 5/22/06 | CY043903 | North American |
| A_shorebird_Delaware_246_2006 | H1N1 | 5/22/06 | CY043911 | North American |
| A_shorebird_Delaware_249_2006 | H9N2 | 5/22/06 | CY043919 | North American |
| A_shorebird_Delaware_350_2006 | H1N1 | 5/23/06 | CY077141 | North American |
| A_shorebird_Delaware_472_2007 | H5N1 | 5/24/07 | CY043935 | North American |
| A_shorebird_Delaware_554_2007 | H9N1 | 5/24/07 | CY077181 | North American |
| A_shorebird_Delaware_Bay_107_2009 | H10N7 | 5/20/09 | CY137777 | North American |
| A_shorebird_Delaware_Bay_111_2009 | H10N7 | 5/20/09 | CY137785 | North American |
| A_shorebird_Delaware_Bay_132_2009 | H10N7 | 5/20/09 | CY137793 | North American |
| A_shorebird_Delaware_Bay_139_2009 | H10N7 | 5/20/09 | CY137801 | North American |
| A_shorebird_Delaware_Bay_167_2009 | H6N1 | 5/20/09 | CY131975 | North American |
| A_shorebird_Delaware_Bay_170_2009 | H1N1 | 5/20/09 | CY137809 | North American |
| A_shorebird_Delaware_Bay_181_2009 | H6N8 | 5/20/09 | CY127846 | North American |
| A_shorebird_Delaware_Bay_226_2009 | H1N7 | 5/20/09 | CY137817 | North American |
| A_shorebird_Delaware_Bay_230_2009 | H6N1 | 5/20/09 | CY127854 | North American |
| A_shorebird_Delaware_Bay_231_2009 | H6N1 | 5/20/09 | CY127862 | North American |
| A_shorebird_Delaware_Bay_236_2009 | H11N1 | 5/20/09 | CY127870 | North American |
| A_shorebird_Delaware_Bay_240_2009 | H10N7 | 5/20/09 | CY137825 | North American |
| A_shorebird_Delaware_Bay_250_2009 | H10N7 | 5/20/09 | CY137833 | North American |
| A_shorebird_Delaware_Bay_255_2006 | H6N1 | 5/22/06 | CY077125 | North American |
| A_shorebird_Delaware_Bay_255_2006 | H6N1 | 5/22/06 | CY127773 | North American |
| A_shorebird_Delaware_Bay_255_2009 | H10N7 | 5/20/09 | CY137841 | North American |
| A_shorebird_Delaware_Bay_257_2009 | H11N8 | 5/20/09 | CY127878 | North American |
| A_shorebird_Delaware_Bay_271_2009 | H10N7 | 5/20/09 | CY137849 | North American |
| A_shorebird_Delaware_Bay_281_2009 | H10N7 | 5/20/09 | CY137857 | North American |
| A_shorebird_Delaware_Bay_293_2008 | H4N6 | 5/16/08 | CY127936 | North American |
| A_shorebird_Delaware_Bay_296_2008 | H4N6 | 5/16/08 | CY126597 | North American |
| A_shorebird_Delaware_Bay_300_2009 | H1N1 | 5/20/09 | CY137865 | North American |
| A_shorebird_Delaware_Bay_309_2008 | H4N6 | 5/16/08 | CY126605 | North American |
| A_shorebird_Delaware_Bay_312_2008 | H4N6 | 5/16/08 | CY127944 | North American |
| A_shorebird_Delaware_Bay_315_2008 | H4N6 | 5/16/08 | CY127952 | North American |
| A_shorebird_Delaware_Bay_322_2009 | H10N7 | 5/20/09 | CY137873 | North American |
| A_shorebird_Delaware_Bay_324_2009 | H1N1 | 5/20/09 | CY137881 | North American |
| A_shorebird_Delaware_Bay_329_2008 | H4N6 | 5/17/08 | CY127960 | North American |
| A_shorebird_Delaware_Bay_332_2006 | H7N3 | 5/23/06 | CY102998 | North American |
| A_shorebird_Delaware_Bay_338_2009 | H10N1 | 5/20/09 | CY137889 | North American |
| A_shorebird_Delaware_Bay_340_2008 | H6N8 | 5/17/08 | CY127814 | North American |
| A_shorebird_Delaware_Bay_340_2009 | H10N1 | 5/20/09 | CY137897 | North American |
| A_shorebird_Delaware_Bay_342_2009 | H10N1 | 5/20/09 | CY137905 | North American |
| A_shorebird_Delaware_Bay_343_2009 | H10N7 | 5/20/09 | CY137913 | North American |
| A_shorebird_Delaware_Bay_351_2009 | H1N9 | 5/20/09 | CY137921 | North American |
| A_shorebird_Delaware_Bay_369_2008 | H10N7 | 5/17/08 | CY136541 | North American |
| A_shorebird_Delaware_Bay_375_2008 | H6N8 | 5/17/08 | CY127822 | North American |
| A_shorebird_Delaware_Bay_379_2008 | H10N8 | 5/17/08 | CY137729 | North American |
| A_shorebird_Delaware_Bay_380_2010 | H6N1 | 5/20/10 | CY127928 | North American |
| A_shorebird_Delaware_Bay_382_2008 | H4N6 | 5/17/08 | CY127968 | North American |
| A_shorebird_Delaware_Bay_395_2008 | H4N6 | 5/17/08 | CY126613 | North American |
| A_shorebird_Delaware_Bay_424_2007 | H13N9 | 5/24/07 | CY127806 | North American |
| A_shorebird_Delaware_Bay_438_2008 | H4N6 | 5/17/08 | CY126621 | North American |
| A_shorebird_Delaware_Bay_446_2008 | H4N6 | 5/17/08 | CY127998 | North American |
| A_shorebird_Delaware_Bay_478_2009 | H6N1 | 5/21/09 | CY127886 | North American |
| A_shorebird_Delaware_Bay_483_2008 | H6N8 | 5/17/08 | CY127830 | North American |
| A_shorebird_Delaware_Bay_485_2008 | H3N2 | 5/17/08 | CY103466 | North American |
| A_shorebird_Delaware_Bay_486_2008 | H4N6 | 5/17/08 | CY128006 | North American |
| A_shorebird_Delaware_Bay_495_2008 | H10N7 | 5/17/08 | CY137737 | North American |
| A_shorebird_Delaware_Bay_497_2008 | H4N6 | 5/17/08 | CY128014 | North American |
| A_shorebird_Delaware_Bay_500_2009 | H10N7 | 5/21/09 | CY137941 | North American |
| A_shorebird_Delaware_Bay_512_2009 | H10N7 | 5/21/09 | CY137949 | North American |
| A_shorebird_Delaware_Bay_513_2006 | H7N3 | 5/23/06 | CY039342 | North American |
| A_shorebird_Delaware_Bay_513_2006 | H7N3 | 5/23/06 | CY103014 | North American |
| A_shorebird_Delaware_Bay_545_2009 | H10N7 | 5/21/09 | CY137957 | North American |
| A_shorebird_Delaware_Bay_549_2009 | H11N1 | 5/21/09 | CY127894 | North American |
| A_shorebird_Delaware_Bay_55_2009 | H10N7 | 5/15/09 | CY137769 | North American |
| A_shorebird_Delaware_Bay_552_2006 | H7N3 | 5/23/06 | CY103022 | North American |
| A_shorebird_Delaware_Bay_555_2006 | H7N3 | 5/23/06 | CY039350 | North American |
| A_shorebird_Delaware_Bay_558_2006 | H1N1 | 5/23/06 | CY137649 | North American |
| A_shorebird_Delaware_Bay_560_2006 | H7N3 | 5/23/06 | CY036774 | North American |
| A_shorebird_Delaware_Bay_586_2008 | H10N7 | 5/18/08 | CY137745 | North American |
| A_shorebird_Delaware_Bay_588_2008 | H4N6 | 5/18/08 | CY128022 | North American |
| A_shorebird_Delaware_Bay_589_2008 | H4N6 | 5/18/08 | CY126629 | North American |
| A_shorebird_Delaware_Bay_604_2008 | H6N2 | 5/18/08 | CY127838 | North American |
| A_shorebird_Delaware_Bay_655_2009 | H1N8 | 6/1/09 | CY137965 | North American |
| A_shorebird_Delaware_Bay_707_2009 | H6N2 | 6/4/09 | CY127902 | North American |
| A_shorebird_Delaware_Bay_756_2009 | H10N7 | 6/4/09 | CY137973 | North American |
| A_shorebird_Delaware_Bay_771_2009 | H1N7 | 6/4/09 | CY137981 | North American |
| A_shorebird_Delaware_Bay_792_2009 | H10N7 | 6/4/09 | CY137989 | North American |
| A_slaty-backed_gull_Japan_6KS0185_2006 | H4N8 | 7/26/06 | CY079298 | North American |
| A_slaty-backed_gull_Japan_6KS0191_2006 | H4N8 | 7/26/06 | CY080238 | North American |
| A_snow_goose_Nunavet_03438_2010 | H3N8 | 7/20/10 | CY138160 | North American |
| A_teal_Chany_444_2009 | H8N8 | 8/29/09 | CY098521 | North American |
| A_teal_Chany_7119_2008 | H15N4 | 8/30/08 | CY098537 | North American |
| A_teal_Oregon_44336-122_2007 | H3N8 | 8/16/07 | CY076036 | North American |
| A_teal_Oregon_44336-130_2007 | H3N8 | 8/16/07 | CY076052 | North American |
| A_western_grebe_Washington_20569-004_2007 | H1N2 | 11/26/07 | CY076164 | North American |
| A_white-fronted_goose_Texas_AI09-6225_2009 | H6N1 | 12/17/09 | CY141080 | North American |
| A_wood_duck_Nova_Scotia_00738_2010 | H3N8 | 8/12/10 | CY139254 | North American |
| A_wood_duck_Wisconsin_10OS2778_2010 | H3N8 | 9/26/10 | CY132964 | North American |
| A_black_bellied_whistling_duck_Colombia_1_2011 | H5N2 | 4/13/11 | AGI02472 | South American |
| A_cinnamon_teal_Bolivia_4537_2001 | H7N3 | 2001 | ABF69265 | South American |
| A_red-winged_tinamou_Argentina_MP1_2008 | H1N1 | 2008/04/ | ACT36601 | South American |
| A_rosy-billed_pochard_Argentina_CIP051-1977_2010 | H6N2 | 6/29/10 | AEL22833 | South American |
| A_rosy-billed_pochard_Argentina_CIP051-269_2007 | H6N8 | 5/13/07 | AEL22844 | South American |
| A_rosy-billed_pochard_Argentina_CIP051-557_2007 | H6N2 | 2007/05/ | ADK78181 | South American |
| A_rosy-billed_pochard_Argentina_CIP051-559_2007 | H9N2 | 2007 | AFF60772 | South American |
| A_rosy-billed_pochard_Argentina_CIP051-575_2007 | H6N8 | 2007/05/ | ADK78192 | South American |
| A_rosy-billed_pochard_Argentina_CIP051-925_2008 | H6N2 | 2008/02/ | ADK78203 | South American |
| A_seagull_Chile_5775_2009 | H13N9 | 11/3/09 | AHA57185 | South American |
| A_semi-palmated_sandpiper_Brazil_43_1990 | H2N1 | 4/18/90 | ABB20151 | South American |
| A_white_faced_whistling_duck_Colombia_1_2011 | H5N2 | 4/11/11 | AGI02461 | South American |
| A_wild_bird_Chile_1805_2008 | H5N9 | 1/21/08 | AHA57197 | South American |
