# Supplementary material for: The Genetic Diversity of Influenza A Viruses in Wild Birds in Peru
Source: PLoS One. 2016 Jan 19;11(1):e0146059. doi: 10.1371/journal.pone.0146059 (PMC4718589; doi:10.1371/journal.pone.0146059)
Supplement: S4 Table — (DOCX) [file pone.0146059.s018.docx]

**Table S4. H13 sequences used in this study.**

| A_American_white_pelican_Minnesota_AI-07-1819_2007 | H13N9 | 2007 |
| --- | --- | --- |
| A_American_white_pelican_Minnesota_Sg-0611_2008 | H13N9 | 7/29/08 |
| A_Larus_argentatus_Astrakhan_458_1985 | H13N6 | 1985 |
| A_Larus_ichthyaetus_Astrakhan_75_1983 | H13N2 | 1983/05/ |
| A_Larus_minutus_Astrakhan_3357_2002 | H13N6 | 9/2/02 |
| A_black_Headed_gull_Astrakhan_65_1983 | H13N6 | 1983 |
| A_black_Headed_gull_Mongolia_1766_2006 | H13N6 | 9/8/06 |
| A_black-headed_gull_Astrakhan_227_84 | H13N6 | 1984 |
| A_black-headed_gull_Georgia_1_2011 | H13N8 | 4/19/11 |
| A_black-headed_gull_Georgia_3_2011 | H13N8 | 4/19/11 |
| A_black-headed_gull_Georgia_4_2011 | H13N8 | 4/19/11 |
| A_black-headed_gull_Georgia_6_2011 | H13N8 | 4/19/11 |
| A_black-headed_gull_Netherlands_1_00 | H13N8 | 2000 |
| A_black-headed_gull_Sweden_1_1999 | H13N6 | 1999 |
| A_black-headed_gull_Sweden_1_2005 | H13N8 | 2005 |
| A_glaucous_gull_Alaska_44199-006_2006 | H13N9 | 9/23/06 |
| A_glaucous_gull_Alaska_44199-097_2006 | H13N3 | 9/25/06 |
| A_glaucous_gull_Alaska_44199-104_2006 | H13N9 | 9/25/06 |
| A_great_black-headed_gull_Astrakhan_1420_79 | H13N2 | 1979 |
| A_great_black-headed_gull_Astrakhan_1421_79 | H13N2 | 1979 |
| A_great_black-headed_gull_Astrakhan_591_82 | H13N2 | 1982 |
| A_Larus_ichthyaetus_Astrakhan_10_1988 | H13N6 | 5/21/88 |
| A_Mongolian_gull_Mongolia_405_2007 | H13N6 | 8/28/07 |
| A_black-legged_kittiwake_Quebec_02838-1_2009 | H13N6 | 8/18/09 |
| A_blackhead_gull_Astrakhan_44_1988 | H13N6 | 5/21/88 |
| A_common_gull_Norway_10_1313_2009 | H13N2 | 2009 |
| A_duck_Hokkaido_WZ68_2012 | H13N2 | 11/1/12 |
| A_Mongolian_gull_Mongolia_401_2007 | H13N6 | 8/28/07 |
| A_glaucous-winged_gull_Southcentral_Alaska_9JR0691R0_2009 | H13N6 | 8/18/09 |
| A_glaucous-winged_gull_Southcentral_Alaska_9JR0738R0_2009 | H13N6 | 8/18/09 |
| A_glaucous-winged_gull_Southcentral_Alaska_9JR0747R0_2009 | H13N6 | 8/18/09 |
| A_glaucous-winged_gull_Southcentral_Alaska_9JR0769R0_2009 | H13N6 | 8/18/09 |
| A_glaucous-winged_gull_Southcentral_Alaska_9JR0781R0_2009 | H13N6 | 8/18/09 |
| A_glaucous-winged_gull_Southeastern_Alaska_9JR0822R0_2009 | H13N6 | 8/19/09 |
| A_great_black_Headed_gull_Atyrau_2966_2008 | H13N6 | 7/13/08 |
| A_great_black-backed_gull_Newfoundland_296_2008 | H13N2 | 10/21/08 |
| A_gull_Astrakhan_226_1984 | H13N6 | 1984/05/ |
| A_gull_Astrakhan_3483_2002 | H13N6 | 9/9/02 |
| A_gull_Astrakhan_998_1990 | H13N6 | 1990/05/ |
| A_great_black-headed_gull_Gurjev_76_83 | H13N2 | 1983 |
| A_herring_gull_Mongolia_454_2008 | H13N8 | 2008/09/ |
| A_gull_Astrakhan_1314_1979 | H13N2 | 1979/05/ |
| A_gull_Astrakhan_176_1986 | H13N2 | 1986/05/ |
| A_gull_Astrakhan_1818_1998 | H13N6 | 1998/05/ |
| A_gull_Astrakhan_1846_1998 | H13N6 | 1998 |
| A_herring_gull_Astrakhan_479_85 | H13N6 | 1985 |
| A_gull_Maryland_704_1977 | H13N6 | 1977 |
| A_duck_Siberia_272_1998 | H13N6 | 1998 |
| A_duck_Siberia_272PF_1998 | H13N6 | 1998 |
| A_black-headed_gull_Georgia_7_2011 | H13N6 | 11/14/11 |
| A_great_black-headed_gull_Atyrau_743_2004 | H13N6 | 7/19/04 |
| A_great_black-headed_gull_Atyrau_744_2004 | H13N6 | 7/19/04 |
| A_great_black-headed_gull_Atyrau_767_2004 | H13N6 | 7/19/04 |
| A_great_black-headed_gull_Atyrau_773_2004 | H13N6 | 7/19/04 |
| A_gull_Maryland_704_1977 | H13N6 | 9/22/77 |
| A_gull_Delaware_AI09-435_2009 | mixed | |
| A_ring-billed_gull_Georgia_AI00-2658_2000 | H13N6 | 12/11/00 |
| A_ring-billed_gull_Quebec_02434-1_2009 | H13N6 | 7/20/09 |
| A_ring-billed_gull_Quebec_02622-1_2009 | mixed | |
| A_ruddy_turnstone_Delaware_Bay_520_1988 | H13N9 | 5/15/88 |
| A_mallard-black_duck_Hybrid_New_brunswick_03736_2009 | H13N6 | 9/14/09 |
| A_silver_gull_Tasmania_062_2006 | H13N6 | 2006 |
| A_yellow-legged_gull_Georgia_1_2010 | H13N2 | 10/18/10 |
| A_yellow-legged_gull_Georgia_1_2011 | H13N6 | 10/28/11 |
| A_ruddy_turnstone_New_Jersey_AI09-294_2009 | H13N6 | 5/11/09 |
| A_shorebird_Delaware_Bay_424_2007 | H13N9 | 5/24/07 |
| A_gull_Maryland_704_1977 | H13N6 | 1977 |
| A_gull_Minnesota_945_1980 | H13N6 | 8/25/80 |
| A_herring_gull_DE_475_1986 | H13N2 | 6/1/86 |
| A_herring_gull_Delaware_660_1988 | H13N6 | 5/16/88 |
| A_herring_gull_NJ_782_1986 | H13N2 | 5/12/86 |
| A_herring_gull_Norway_10_2336_2006 | H13N6 | 2006 |
| A_hooded_merganser_New_brunswick_03750_2009 | H13N6 | 9/14/09 |
| A_laughing_gull_Delaware_Bay_2838_1987 | H13N2 | 5/19/87 |
| A_laughing_gull_Delaware_Bay_2838_1987 | H13N2 | 5/19/87 |
| A_laughing_gull_New_Jersey_Sg-00485_2008 | H13N9 | 5/15/08 |
| A_laughing_gull_New_Jersey_Sg-00559_2008 | H13N9 | 5/28/08 |
| A_laughing_gull_New_Jersey_Sg-00568_2008 | H13N9 | 5/28/08 |
| A_mallard_Korea_SH38-45_2010 | H13N2 | 3/22/10 |
| A_shorebird_Delaware_221_2006 | H13N9 | 5/22/06 |
| A_seagull_Chile_5775_2009 | H13N9 | 11/3/09 |
| A_shorebird_DE_68_2004 | H13N9 | 5/17/04 |
| A_shorebird_Delaware_224_2006 | H13N9 | 5/22/06 |
| A_lesser_snow_goose_Alaska_44199-115_2006 | H13N9 | 9/24/06 |
| A_kelp_gull_Argentina_LDC4_2006 | H13N9 | 2006 |
