# Supplementary material for: The Genetic Diversity of Influenza A Viruses in Wild Birds in Peru
Source: PLoS One. 2016 Jan 19;11(1):e0146059. doi: 10.1371/journal.pone.0146059 (PMC4718589; doi:10.1371/journal.pone.0146059)
Supplement: S5 Table — (DOCX) [file pone.0146059.s019.docx]

**Table S5**. **Viral introductions observed during each year from 2006-2010**

| **YEAR** | **INTRO** | **VIRUS NAME** | **DATE** | **LINEAGE^a^** |
| --- | --- | --- | --- | --- |
| 2006 | intro 1 | A/duck/Peru/32/2006/H3N8 | 11/16/06 | North American (b) |
| 2007 | intro 1 | A/duck/Peru/MM17/2007/H4N5 | 2/6/07 | North American (b) |
|  |  | A/duck/Peru/MM23/2007/H4N5 | 2/13/07 |  |
|  | intro 2 | A/whimbrel/Peru/P41/2007/H13N2 | 11/20/07 | Eurasian |
|  |  | A/gull/Peru/P43/2007/H13N2 | 11/27/07 |  |
| 2008 | intro 1 | A/duck/Peru/PV80/2008/H2N9 | 7/22/08 | North American (b) |
|  | intro 2 | A/duck/Peru/PV72/2008/H7N3 | 7/15/08 | North American (other) |
|  | intro 3 | A/ruddy turnstone/Peru/MM149/2008/H10N7 | 10/29/08 | North American (a) |
|  |  | A/oystercatcher/Peru/MM152/2008/H10N7 | 11/5/08 |  |
|  |  | A/duck/Peru/P114/2009/H3N2 | 5/12/09 |  |
|  | intro 4 | A/ruddy turnstone/Peru/PuV51/2008/H12N5 | 4/22/08 | North American (b) |
|  |  | A/ruddy turnstone/Peru/PuV52/2008/H12N5 | 4/22/08 |  |
| 2009 | intro 1 | A/curlew/Peru/PuV178/2009/H3N1 | 11/24/09 | North American (b) |
|  |  | A/gull/Peru/PuV172/2009/H1N1 | 11/10/09 |  |
|  |  | A/whimbrel/Peru/CH27/2009/H1N1 | 11/18/09 |  |
|  | intro 2 | A/ruddy turnstone/Peru/PuV182/2009/H3N1 | 11/24/09 | North American (b) |
|  |  | A/ruddy turnstone/Peru/PuV181/2009/H3N8 | 11/24/09 |  |
|  | intro 3 | A/duck/Peru/PuV196/2009/H10N2 | 12/14/09 | North American (a) |
|  | intro 4 | A/moorhen/Peru/120/2009/H1N9 | 5/19/09 | North American (a) |
|  | intro 5 | A/gull/Peru/CH02/2009/H13N2 | 9/13/09 | North American (b) |
| 2010 | intro 1 | A/gull/Peru/CH134/2010/N6 | 12/9/10 | North American (b) |
|  | intro 2 | A/egret/Peru/CH50/2010/H6N8 | 3/25/10 | North American (b) |
|  |  | A/willet/Peru/CH49/2010/H6N8 | 3/25/10 |  |
|  | intro 3 | A/duck/Peru/CH36/2010/H11N9 | 3/9/10 | North American (b) |
|  | intro 4 | A/black skimmer/Peru/CH55/2010/H13N2 | 4/6/10 | Global |
|  | intro 5 | A/gull/Peru/CH121/2010/H13N2 | 12/2/10 | North American (b) |
|  | intro 6 | A/gull/Peru/CH98/2010/H13 | 10/27/10 | Argentinian |

^a^Lineage as shown in PA segment phylogeny (Fig 2)
