# Supplementary material for: The Genetic Diversity of Influenza A Viruses in Wild Birds in Peru
Source: PLoS One. 2016 Jan 19;11(1):e0146059. doi: 10.1371/journal.pone.0146059 (PMC4718589; doi:10.1371/journal.pone.0146059)

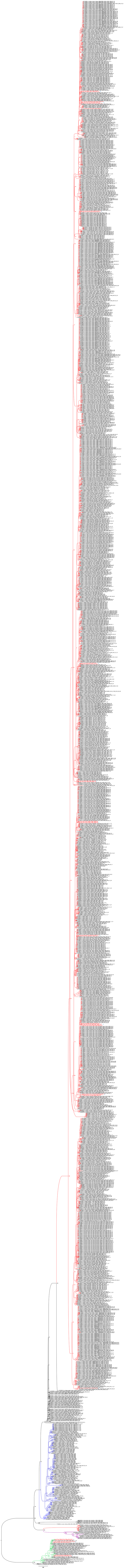

Supplement: S10 Fig — Identical tree as S4 Fig, only tip labels have been indicated and clades are expanded in full. Lineages are colored as per Figs 1 and 2, S2–S6 Figs. Peruvian sequence tips are indicated in red. Viral introduction events into Peru are indicated with a blue circle. All bootstrap values are shown. Scale bar indicates number of nucleotide substitutions per site. (PDF) [file pone.0146059.s010.pdf]
